# Brain Signal for Secure EEG Biometric Authentication: A Comprehensive Survey

**DOI:** 10.3390/s26134045

**Published:** 2026-06-25

**Authors:** Marissa L. de Ataide, Narayan Vetrekar, Krishna Patel, Rajendra Gad, Raghavendra Ramachandra

**Affiliations:** 1 School of Physical and Applied Sciences, Goa University, Taleigao 403206, Goa, India; marissaataide@unigoa.ac.in (M.L.d.A.); vetrekarnarayan@unigoa.ac.in (N.V.); elect.krishna@unigoa.ac.in (K.P.); rsgad@unigoa.ac.in (R.G.); 2 SAFE Center, Norwegian University of Science and Technology (NTNU), 7491 Gjøvik, Norway

**Keywords:** brain signals, biometric, authentication, verification, identification, EEG devices, acquisition protocol, database, preprocessing, feature extraction, classification, challenges

## Abstract

Electroencephalography (EEG) has emerged as a promising modality for biometric user authentication due to its inherent uniqueness and resistance to spoofing attacks. Significant advances in brain wave signal analysis over recent years have reinforced its potential as a distinctive and reliable biometric trait. However, a comprehensive evaluation of the overall progress in this field remains limited. To address this gap, this paper presents an in-depth survey of EEG-based user authentication systems. The survey begins with a comprehensive overview of the human brain’s structure and functional organization, followed by a discussion of EEG signal acquisition principles and commonly used recording devices. It provides a detailed review of data acquisition protocols, publicly and proprietary available EEG databases, and essential preprocessing techniques required for effective signal refinement. The paper further examines feature extraction strategies and classification algorithms employed in EEG-based biometric authentication. In addition to reviewing existing methodologies, the survey identifies key challenges and future considerations in EEG biometrics, such as signal variability, age, mental health conditions, inter-session and inter-subject variability, etc, to establish stable and robust algorithms. This work serves as a foundational reference for researchers, outlining current progress and presenting a structured roadmap for future advancements in EEG-based biometric systems.

## 1. Introduction

A reliable user authentication system is essential for many digital applications that require secure access to protect sensitive personal and organizational data [1]. User authentication functions as a core fundamental security mechanism that authenticates an individual’s identity based on the provided credentials, thereby ensuring that only authorized users are granted access to protected resources [2]. With the rapid advancement of technology and the increasing reliance on digital platforms, secure user authentication has become a critical requirement across sectors, including national security, banking, healthcare, and e-commerce [3]. However, traditional and weak authentication mechanisms, often make it easier for attackers to gain easy unauthorized access to individuals’ accounts, leading to the exfiltration of sensitive information. Consequently, the development of robust authentication methodologies has been a key focus in the field of information security over many decades [4].

Historically, authentication has relied on knowledge-based and possession-based approaches, including passwords, Personal Identification Numbers (PINs), usernames, and physical tokens such as identity cards and ATM cards [5]. Despite their widespread adoption, these methods have demonstrated significant vulnerabilities, including susceptibility to phishing, brute-force attacks, credential leakage, card cloning, and large-scale data breaches, proving them to be a highly unreliable mechanism for user authentication [6,7,8].

The growing security concerns have led to the exploration of biometric authentication, where human-intrinsic features such as face, fingerprints, iris, voice, ear, hand geometry, keystroke dynamics, signature, and DNA are used for identity authentication [9]. Compared to password and token-based mechanisms, biometric systems provide a stronger and more reliable means of authentication, as these traits are unique and inherent to individuals. Despite having merit over traditional approaches, biometric authentication systems are not entirely foolproof and secure. In recent years, several security vulnerabilities have emerged, particularly in the form of presentation attacks, where impostors use biometric artifacts to go past authentication systems [10,11]. Many biometric traits are susceptible to falsification because they are externally visible [12]. For example, fingerprints [11] can be lifted from objects that individuals touch, facial images can be extracted from social media and used to fool Facial Recognition Systems (FRS) [10], and voice authentication [13] can be spoofed using machine learning-based text-to-speech models [14]. These weaknesses pose serious security concerns, as attackers can exploit them to gain unauthorized access to sensitive systems.

To address these limitations of biometric-based authentication, researchers have increasingly explored the use of biopotential signals such as Electroencephalogram (EEG), Electromyogram (EMG), and Electrocardiogram (ECG) for user authentication. Among these, EEG-based brain wave signals [15,16] have emerged as one of the most promising biometric modalities due to their uniqueness, inherent difficulty in replication, and resistance to traditional spoofing techniques [17,18].

EEG signals, a type of behavioral biometric trait, measure the brain’s ionic potentials through scalp electrodes that are in direct physical contact with the scalp for signal acquisition. This significantly reduces the possibility of remote acquisition, unauthorized access, or biometric data theft. These EEG signals are unique to each individual, and are strongly influenced by an individual’s cognitive state, emotions, and mental activity, making it considerably more difficult for attackers to fabricate or replicate valid EEG patterns. They can only be obtained from a live person [4]. No two individuals possess identical EEG patterns [19,20], and even the same individual’s signals may vary under different acquisition conditions. These intrinsic characteristics make EEG-based user authentication methods more resistant to presentation attacks [21], as EEG signals are generally harder to fake compared to conventional biometric modalities. These EEG signals offer high temporal resolution [22] but relatively poor spatial resolution, meaning that the timing of brain activity can be accurately detected while the precise source location remains difficult to determine. Additionally, EEG signals are influenced by factors such as health and age [23].

**Figure 1:**
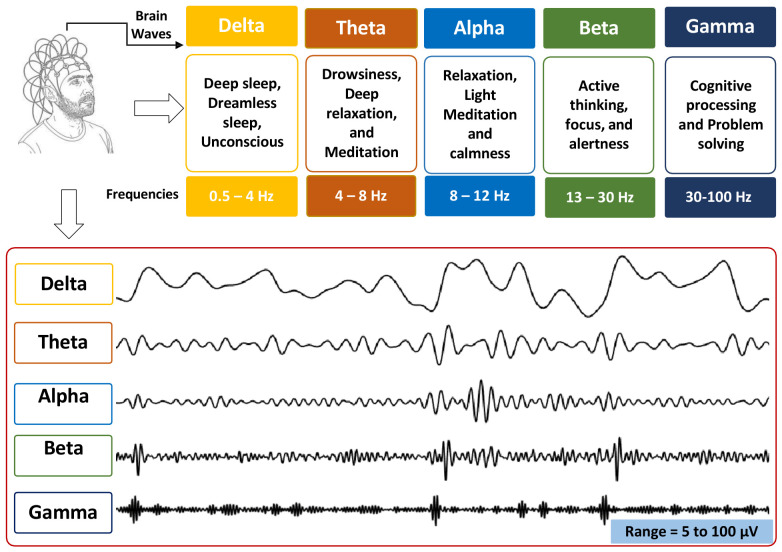
Illustrating the dominant bands of frequencies (Delta, Theta, Alpha, Beta, and Gamma) and amplitudes (5–100 µV) of EEG waveforms in the different regions of the brain, presenting the mental behavior of the person.

Due to these inherent security advantages, EEG-based authentication has attracted increasing research attention in recent years [2,24]. In addition to security-related aspects, recent studies have also investigated challenges associated with cross-session variability, robustness, and practical real-world deployment of EEG-based authentication systems. A typical illustration of EEG signals acquired for an individual user across different frequency bands is shown in Figure 1.

### Evolution in EEG for Biometrics

Employment of brain wave signals for biometric authentication has progressed steadily since the early 20th century, evolving from basic neurological research into a viable method for secure identity authentication. The foundation was laid in 1924 when Hans Berger recorded and identified distinct brain waveforms, such as alpha and beta rhythms, marking the birth of electroencephalography (EEG) as a tool for studying human brain patterns [25]. During these early years, EEG was primarily used for clinical and neurological studies, with no consideration given to employing it as a biometric trait. However, theoretical findings about the uniqueness of EEG patterns gradually encouraged researchers to explore brain signals as potential biometric modalities by the 1980s and 1990s [26,27,28].

In the early 2000s, EEG-based user authentication was first demonstrated using mental tasks, and researchers began extracting features such as power spectral density (PSD) and autoregressive (AR) coefficients [29,30], opening broader possibilities for EEG-based identity authentication. Between 2007 and 2010, various studies began leveraging Event-related Potentials (ERPs), particularly Visual Evoked Potentials (VEPs) and mental imagery tasks, to enhance the reliability of authentication [20,30].

From 2010 to 2015, advances in signal processing enabled more accurate user differentiation, with the adoption of wavelet transforms, autoregressive models, and classification algorithms such as Support Vector Machines (SVM) and Linear Discriminant Analysis (LDA). With the promising results obtained using ERPs, researchers increasingly expanded their focus to ERP-based biometric systems, even exploring EEG recordings during sleep to identify stable and distinctive biometric patterns [22,31].

**Figure 2:**
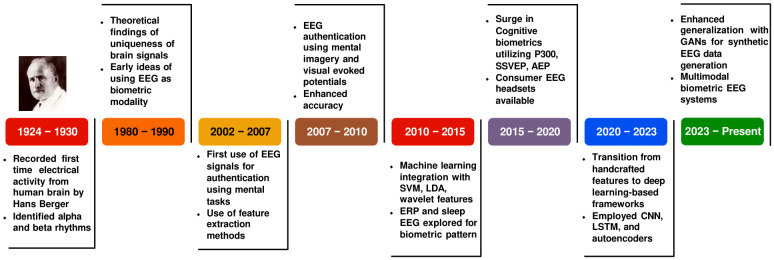
The progress of EEG from its inception in 1924 to date and the emergence of EEG for biometrics.

The period from 2015 to 2020 marked a significant surge in cognitive biometrics, where task-induced EEG responses such as P300, SSVEP, and Auditory Evoked Potentials were increasingly leveraged to enhance system robustness [32]. This phase also coincided with the rise of portable consumer-grade EEG headsets, which broadened experimental accessibility and practical deployment. Post-2020, research shifted toward deep learning approaches, employing Convolutional Neural Networks (CNNs), Recurrent Neural Networks (RNNs), and autoencoders to learn subject-specific representations directly from raw EEG signals [33]. Recent studies have demonstrated the effectiveness of Generative Adversarial Network (GAN)-based frameworks in generating realistic EEG patterns that preserve class discriminative features, facilitating the generation of synthetic EEG data to enhance generalization of the model [34]. Furthermore, multimodal EEG biometric systems are gaining increasing attention in very recent years for their ability to enhance authentication accuracy by combining EEG signals with other physiological or behavioral traits [34]. The research progress of EEG from its clinical inception in 1924 to its current role in biometric systems is illustrated in Figure 2.

**Figure 3:**
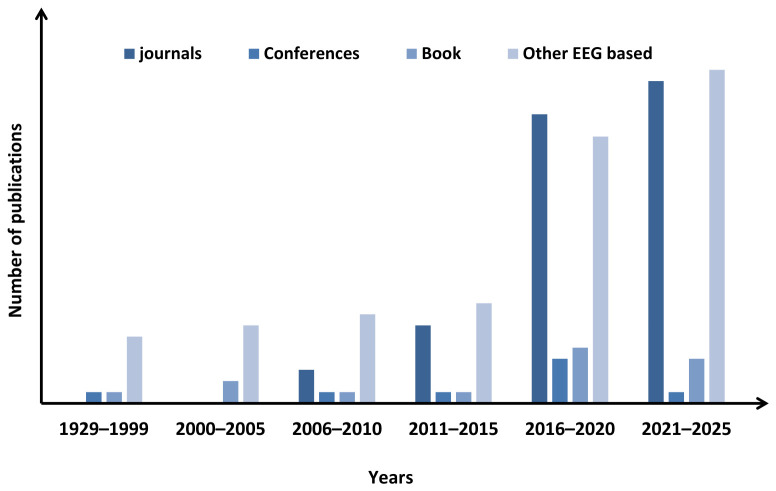
Distribution of the EEG biometrics publications included in this survey, categorized by type (journals, conferences, books, and other EEG-based works) over different time periods.

To provide an overview of the research findings published in EEG biometrics, we categorized the selected papers by publication type and grouped them across different time spans. The resulting distribution is presented in Figure 3. As seen in Figure 3, the early phase of EEG biometrics research (before 2010) was characterized by a relatively small number of contributions, primarily in book chapters and other EEG-related studies. From 2016 onwards, there has been a notable rise in journal publications, along with a parallel increase in other EEG-based works. This trend within the surveyed papers highlights a growing research interest and reflects the consolidation of EEG biometrics as a recognized field in the broader domain of biometric authentication.

Although EEG holds significant promise for user authentication, its widespread adoption remains limited due to technological constraints, high costs, and inherent signal variability. Consequently, this behavioral biometric modality still lacks extensive research, underscoring the need for comprehensive and up-to-date survey studies. Existing survey papers generally discuss the core components of EEG-based biometric systems, including data acquisition practices, publicly available datasets, preprocessing techniques, feature extraction methods, and classification approaches. They further address experimental protocols, EEG-based cryptographic frameworks, authentication methodologies, and challenges such as noise sensitivity and signal variability. However, earlier reviews often do not examine these aspects with sufficient depth and breadth [15,16,29,32,35]. A comparative study of previous survey papers, presented in Table 1, highlights their coverage relative to the present survey.

Motivated by these gaps, we present a comprehensive survey of EEG biometrics, encompassing electrode systems, EEG acquisition devices, public and proprietary datasets, and data acquisition protocols. The survey critically discusses the strengths, limitations, robustness, and interpretability of different preprocessing, feature extraction, and classifier techniques with their analytical comparison. Furthermore, the evaluation metrics and issues related to EEG biometric security, cross-session variability, benchmarking inconsistencies across datasets and evaluation protocols, and practical deployment challenges are comprehensively discussed to provide deeper scientific insight into the current state of EEG-based authentication systems.

To ensure a systematic and transparent review process, a PRISMA-inspired methodology was adopted for the identification, screening, eligibility assessment, and inclusion of relevant studies related to EEG-based biometric authentication. Research articles (total 1685) were collected from three scholarly databases: Google Scholar (497 papers), IEEE Xplore (1099 papers), and ScienceDirect (89 papers), using predefined search keywords such as “EEG”, “biometrics”, “Electroencephalography”, “Authentication”, “Recognition”, “Verification”, “Identification”, “EEG authentication”, and “EEG-based user authentication”.

**Figure 4:**
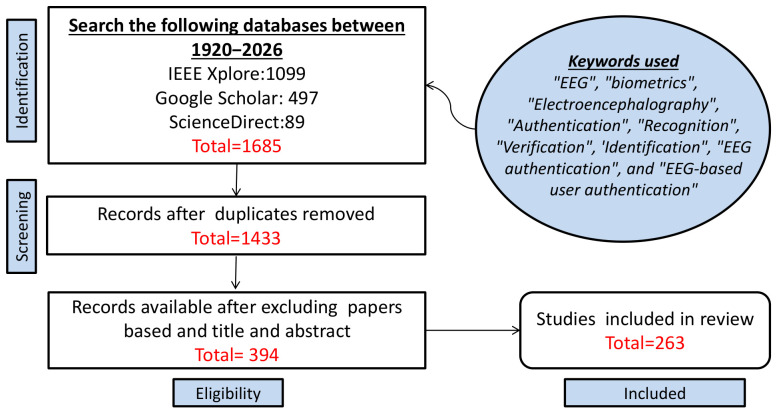
PRISMA flow diagram showing the literature selection and screening process employed in this survey.

Duplicate records retrieved from multiple databases were removed during the screening process, resulting in 1433 unique papers. Subsequently, studies unrelated to biometric authentication were excluded through title and abstract screening, resulting in a final set of 394 papers for further analysis. After full-text screening, a final set of 263 papers on EEG biometrics was available. The overall literature selection and filtering procedure employed in this survey is illustrated in Figure 4.

In this work, EEG-based biometric authentication is addressed in both verification (1:1 matching) and identification (1:N matching) settings, where a claimed identity is either accepted or rejected in verification, and the most likely identity is determined in identification. We summarize the major contributions of this survey article as follows:

- It provides a comprehensive and analytical review of EEG biometric systems, covering EEG devices, electrode technologies, data acquisition protocols, preprocessing techniques, feature extraction methods, and classification approaches, together with a critical comparison of their strengths, limitations, robustness, interpretability, and deployment feasibility.
- It presents a comprehensive survey of state-of-the-art EEG biometric authentication research published from 1924 to 2026, including a historical analysis of the evolution from early EEG recording technology to modern EEG-based biometric systems.
- It provides an extensive review of publicly available and proprietary EEG datasets, together with discussions on experimental design considerations and data acquisition methodologies used in biometric authentication studies.
- It offers a comprehensive analysis of evaluation protocols and performance assessment methodologies, including identification and verification frameworks, performance metrics, benchmarking challenges, and the need for standardized evaluation practices for fair comparison across studies.
- It examines emerging challenges and future research directions in EEG biometrics, including biometric security, cross-session robustness, demographic and physiological variability, practical deployment constraints, privacy and ethical concerns, explainable AI, multimodal biometrics, and BCI integration.

The rest of the paper is organized as follows: Section 2 presents the details on the EEG biometric system with a brief description of the enrollment and probe process employed in user authentication. Section 3 details the characteristic properties of brain wave signals and different bands of frequencies generated due to the brain activity. Section 4 categorizes electrode systems based on types of electrodes, and discusses the electrode placement standards that vary from low-density configurations to ultra-high-density layouts. Section 5 discusses the different types of EEG data acquisition paradigms employed for collecting brain signals under different stimulation conditions. Section 6 summarizes the publicly available and proprietary EEG database for biometric research. Section 7 presents the detailed preprocessing methods employed so far in the literature. Section 8 introduces the time-domain, frequency-domain, time-frequency, and deep features extraction methodologies. Further, Section 9 describe the different classification methods, such as similarity, handcrafted, shallow networks, and deep learning networks for EEG-based user authentications. Towards the end, Section 10, Section 11, Section 12 and Section 13 present the experimental evaluation protocols, performance metrics, recent challenges, future works, and concluding remarks.

## 2. EEG Biometric System

**Figure 5:**
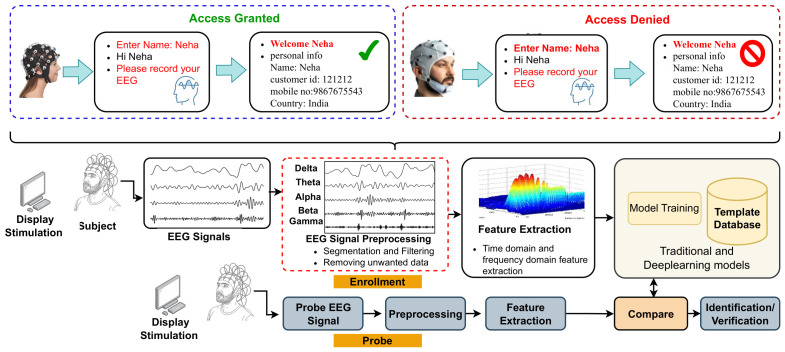
A generalized EEG-based biometric system operates through two fundamental phases: an Enrollment phase, where individual EEG signatures are captured and modeled, and a Probe phase, where incoming EEG data are verified against the stored templates.

EEG biometrics uses electrical signals recorded based on the mental activity of the brain for authentication. Like any other biometric system, the EEG biometric system consists of four basic steps, which are EEG signal acquisition, preprocessing, feature extraction, and classification, as shown in Figure 5. A brief description about each step in the EEG biometric system is provided in this section.

*EEG Data Acquisition* is the first step in which the subjects perform the task or view a stimulation using a computer display, and the acquisition is performed using an EEG device (Table 5 shows different EEG devices and their details are discussed in Section 4). Normally, data acquisition is carried out in a controlled room with few disturbances, and the subjects are asked to sit in the resting position and avoid unnecessary movements. After the subject is in a relaxed position, the electrode cap is placed on the subject’s head, and brain signals are acquired using suitable EEG recorders. The stimulation shown to the subjects to generate cognitive brain wave signals is mainly based on the resting state [4,36], Event-Related Potential [17,37,38,39], and mental activities [24,40]. To acquire proper brain activity and to attain optimal EEG signals, the subjects are recommended to get adequate sleep, avoid stress, and avoid strenuous physical activity prior to the data acquisition.

*Preprocessing* is the second step after data acquisition, which converts EEG signals into a suitable format for further analysis. The EEG signals acquired from the brain are not very accurate; a lot of spatial information gets lost, and these signals might contain artifacts due to eye blinking [41], muscle movement [40], etc. Preprocessing involves cleaning the data, removing unwanted spikes, filtering the data by applying suitable spatio-temporal filters [39,42], reducing the size of the data [43], etc. There is no universally adopted EEG preprocessing pipeline, which gives researchers the freedom to choose how they want to visualize and transform their acquired raw EEG signals. One of the notable tools available to preprocess the brain signals is the EEGlab [17,38,40].

*Feature Extraction* is applied on the preprocessed signals to leverage the most discriminating characteristic features of brain signals. Feature extraction helps transform these raw signals into a simplified and structured form that captures the most relevant information about brain activity and enables effective classification. Feature extraction can be performed using various methods such as time domain [4], frequency domain [44], time-frequency domain [45], and deep features [46]. Some of the commonly used feature extraction techniques on EEG signals are Power Spectral Density(PSD) [47], Fast Fourier Transforms (FFT) [48,49], and Wavelet Transform [45].

*Classification* is the final stage of the EEG-based authentication system. Once the EEG signals are preprocessed and the desired features are extracted, these signals are then processed to train the classifier model. The classifiers, which are based on conventional methods such as Support Vector Machine (SVM) [17], Decision Tree, and Random Forest [15], were employed in the earlier studies. Further, deep learning methods such as CNN [50] and LSTM [51] have gained prominence for their ability to automatically extract and learn hierarchical features from raw EEG signals, leading to enhanced authentication performance.

These four basic steps of biometric systems are employed in two phases: Enrollment and Probe. During the *Enrollment* phase, the EEG signals acquired from multiple subjects undergo preprocessing, followed by feature extraction. A Machine Learning (ML) model or a Deep Learning (DL) classifier model is then trained to learn unique patterns from the extracted EEG features to ensure that the system recognizes and differentiates between the individuals. The trained model then generates a fixed-length feature vector for each subject, which is saved in a secure database template. During the *Probe* phase, the new EEG signal of the subjects is processed through the same pipeline and then compared with the database template to verify the user’s identity. An illustration of the *Enrollment* and *Probe* phases is provided in Figure 5.

## 3. Characteristic Properties of Brain Waves

The human brain is a highly complex and dynamic organ that generates brain wave signals, reflecting various cognitive abilities due to its unique neural connectivity across different functional regions [52,53]. As a result, the neuronal signals produced in response to external stimuli vary across individuals, making EEG signals distinctive for each person. However, for the same individual under identical external stimulations, brain wave signals tend to remain relatively stable over a time [54].

**Figure 6:**
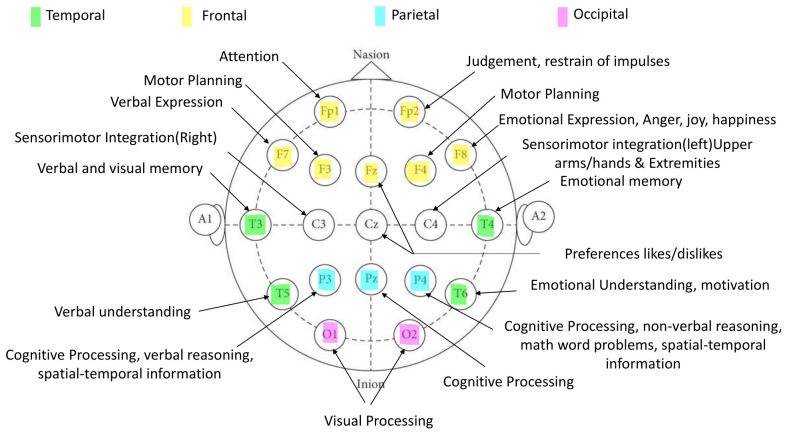
Anatomical placement and functions of each electrode in different regions of the brain in the EEG system based on the international 10–20 system.

The brain wave signals are generated from three different parts of the brain [55]: cerebrum, cerebellum, and brainstem. The *cerebrum (or cerebral cortex)* is responsible for initiating and coordinating movements, regulating temperature, enabling speech, judgment, thinking, reasoning, vision, hearing, touch, and other senses [56]. It comprises four main regions, namely, the frontal, temporal, parietal, and occipital lobes, which perform its responsibilities that are highlighted in Table 2. In EEG terminology, an additional central region (e.g., electrodes C1, Cz, and C2) is often distinguished functionally between the frontal and parietal lobes, corresponding to the sensorimotor cortex responsible for movement and touch perception, which is also summarized in the Table 2. The *cerebellum*, which is the size of a fist, is located at the back of the head, below the temporal and occipital lobes, and above the brainstem. Its functions include the coordination of voluntary muscle movements, posture, balance, and equilibrium maintenance [57,58,59]. The *brainstem* connects the cerebrum with the spinal cord, and facilitates functions such as hearing and movement in calculating responses and environmental changes, tear production, chewing, blinking, facial expression, and bodily activities [60]. Although the *cerebellum* and *brainstem* are responsible for numerous essential neural functions, they do not meaningfully contribute to scalp EEG recordings. This is because EEG primarily captures synchronous postsynaptic activity of large populations of cortical pyramidal neurons near the cortical surface; deep structures such as the *cerebellum* and *brainstem* are farther from the electrodes, tend to produce less synchronous activity, and their electrical fields are strongly attenuated and spatially smeared by the cerebrospinal fluid, skull, and scalp [61]. A summary of all the parts of the brain discussed above is provided in Table 2, with the electrodes placed to acquire these signals displayed in Figure 6. The electrode placement shown in the figure is based on the 10–20 international system which will be discussed in detail in Section 4.2.

The characteristic signals generated by different regions of the brain are primarily dependent on neural activity, which is influenced by various factors such as external stimuli, mental state, emotional states, and stress levels [62]. Electrodes used in electroencephalography (EEG) measure the electrical activity resulting from the combined excitatory and inhibitory postsynaptic potentials of large groups of neurons firing synchronously. It is important to note that EEG does not capture the activity of single neurons, but rather reflects the aggregate neural activity, which manifests as oscillating electrical voltages in the microvolt range [63]. These neural oscillations give rise to voltage fluctuations across different points on the scalp, producing varying electrical potentials. The amplitude of these EEG signals typically ranges from 5 to 100 µV, and the frequency spectrum extends from 0.5 Hz to 40 Hz (Figure 1). This broad frequency band, corresponding to distinct physiological and cognitive states ranging from deep sleep to high-level cognitive processing, is conventionally categorized into five distinct types of brain waves:

- Gamma (30 Hz and above)—Associated with cognitive processing and problem solving.
- Beta (13–30 Hz)—Linked to active thinking, focus, and alertness.
- Alpha (8–12 Hz)—Present during relaxation and calmness, eyes-closed resting condition.
- Theta (4–8 Hz)—Related to drowsiness, deep relaxation, and meditation.
- Delta (0.5–4 Hz)—Dominant during deep sleep, unconscious state, and deep restorative processes

Each of these waveforms is associated with specific cognitive and behavioral states. For instance, beta waves are dominant during active thinking and focus, while alpha waves emerge during relaxed wakefulness. These frequency bands form the foundation for EEG signal analysis in neuroscience, cognitive science, and EEG-based biometric authentication systems. The dominance of these waves varies depending on the task being performed and the corresponding active brain regions, as summarized in Table 3.

## 4. Electrode Systems

The brain wave signals are acquired by placing low-impedance electrode systems on the scalp of the brain to acquire the electrical signals generated due to brain activity [64]. Electrodes are small metal discs having thin wires attached to acquire the ionic current flow due to the activities in the brain cells. There are different types of electrodes, and according to the standards, the placement of electrodes is done on the scalp to acquire the signals.

### 4.1. Categories of Electrodes

There are mainly three different categories of electrodes used in the EEG system, and they are based on hydration, material, and functionality. Table 4 presents a summary of the different categories of EEG electrodes, providing information on their types, lifespan, signal quality, impedance levels, and skin preparation.

#### 4.1.1. Based on Hydration Mechanism

EEG electrodes can be classified based on their hydration method, adapted to ensure adequate ionic conductivity through the skin–electrode interface [65,66,67]. They are grouped into wet [24,68], dry [50], and semi-dry electrodes [69]. Each type has its merits and demerits.

*Wet electrodes* use the electrolyte as a medium, typically based on conductive gel, paste or saline to reduce skin–electrode impedance. They offer excellent signal quality, low contact impedance and are considered to be a gold standard for stable signal recording. However, their preparation is time-intensive and the electrolytic gel dries over time requiring reapplication of gel, which causes discomfort for long-term use [24,68].

*Dry electrodes*, as the name suggests, do not require any conductive gel or liquid and rely on direct contact between conductive materials and the scalp. Their performance depends heavily on mechanical design. Usually, they are available in two designs: rigid pin-type and flexible polymer electrodes. Rigid pin-type electrodes penetrate the hair better, while flexible polymer electrodes are soft and enhance comfort. However, they generally exhibit higher impedance, motion sensitivity, and lower Signal-to-Noise Ratio (SNR) than wet electrodes [50].

*Semi-dry electrodes* offer a balance between wet and dry electrodes. They use a very small amount of liquid or electrolyte stored within the absorbent sponges, that slowly release moisture. They are designed to reduce impedance without needing reapplication of gel. However, managing consistent hydration levels and ensuring re-usability remains a technical challenges [69].

From a biometric authentication perspective, wet electrodes generally achieve the highest recognition accuracy due to their superior signal stability and low impedance, particularly in controlled laboratory environments. However, their long preparation time and poor user convenience significantly limit scalability and real-world adoption. Dry electrodes, although more practical for wearable and mobile authentication systems, often experience performance degradation under motion artifacts, hair interference, and prolonged recordings. Semi-dry electrodes have recently emerged as a promising compromise by balancing signal quality and usability, making them increasingly attractive for practical EEG biometric systems. These trends suggest that electrode selection in EEG biometrics involves a trade-off between acquisition quality, user comfort, and deployment feasibility rather than a universally optimal solution.

#### 4.1.2. Based on Electrode Materials

EEG electrodes are classified based on the material used in their construction, which affects signal quality, durability, and biocompatibility. Among metallic electrodes, silver/silver chloride (Ag/AgCl) [17] electrodes are the most commonly used in EEG biometrics due to their low impedance, stable signal transmission, and corrosion resistance [70]. They are suitable for wet electrodes in long-term recordings. Gold electrodes, on the other hand, offer excellent conductivity and good signal quality but are expensive to use for data acquisition [71]. Tin electrodes provide a low-cost alternative but are more prone to oxidation and exhibit comparatively higher impedance, which may affect long-term reliability. Further, polymer-based electrodes provide flexibility and biocompatibility, while carbon-based electrodes, due to the material property, provide skin-friendly electrodes with improved comfort and reduced motion artifacts. Although, the choice of material depends on factors such as signal clarity, cost, and comfort, tailored to specific EEG applications, Ag/AgCl electrodes remain the preferred choice for high-accuracy compared to other electrodes [72] because of their stable electrochemical properties and reliable long-term recordings. Although gold and platinum electrodes provide excellent conductivity and durability, their high cost limits widespread deployment in consumer-oriented systems. In contrast, conductive polymer and carbon-based electrodes improve flexibility and comfort, particularly for wearable EEG systems, but their long-term signal consistency and robustness under varying environmental conditions remain active research challenges. These observations highlight the growing emphasis on balancing signal fidelity with comfort and portability in modern EEG biometric acquisition systems.

#### 4.1.3. Based on Functionality

Electrodes in EEG-based biometric systems can be classified according to their functionality into active, passive, hybrid, reference, and ground electrodes. Among these, active, passive, and hybrid electrodes are most relevant and are therefore included in the comparison Table 4. *Active electrodes* contain a built-in preamplifier near the electrode site and require a power source for the amplification circuit. By reducing noise and interference at the point of acquisition, they enhance the Signal-to-Noise Ratio and provide more stable recordings even under variable skin–electrode contact. Since they can be implemented with dry, semi-dry, or minimal gel designs, active electrodes are particularly well suited for biometric-related applications, where reduced setup time, user comfort, and robustness against motion artifacts are crucial [73].

*Passive electrodes*, in contrast, do not include built-in amplification and instead transmit raw signals to an external amplifier. They typically require conductive gel or paste to lower skin–electrode impedance and, although more affordable and widely used in clinical EEG, diagnostics, and sleep studies, their higher susceptibility to noise and greater preparation time limit their direct applicability in real-world biometric systems [74,75].

*Hybrid electrodes* represent a functional integration of both active and passive or wet and dry designs, aiming to combine their advantages while mitigating individual drawbacks. For example, semi-dry electrodes that incorporate small electrolyte reservoirs or conductive polymer-based layers can deliver signal quality close to wet electrodes while offering the usability of dry electrodes. Such hybrid solutions are gaining attention in EEG biometrics, as they provide a balance between accuracy, comfort, and long-term wearability, making them well suited for portable and user-friendly authentication devices [76,77,78].

Reference and ground electrodes [17] are also integral parts of an EEG system, serving as baseline and stabilization points, respectively; however, they are not included in the comparison table since their role is supportive rather than directly influencing performance factors such as lifespan, signal quality, or user comfort.

Generally, active and hybrid electrode systems are increasingly favored in EEG biometrics because they provide improved robustness against motion artifacts and impedance variability, which are critical for real-world authentication scenarios. Passive electrodes remain effective in controlled laboratory studies but often require extensive preparation and stable acquisition conditions. Consequently, recent EEG biometric research increasingly focuses on portable active and hybrid electrode configurations that can maintain acceptable authentication performance while improving usability and reducing setup complexity.

A summary of all the different types of electrodes based on hydration, material, and functionality is presented in Table 4. A comparison of signal type, impedence, skin preparation, and lifespan is provided in the table.

### 4.2. Electrode Placement Standards

EEG-based biometric authentication identifies individuals using unique brain wave patterns, with electrode placement playing a key role in signal quality and resolution. While early neurophysiological recordings began in 1875 on animals [79], human EEG was first recorded in 1924 using silver foil electrodes [25]. To standardize acquisition, the 10–20 system was first introduced in 1947 [80], involving placing electrodes proportionally based on four anatomical landmarks: the nasion, inion, and the left and right pre-auricular points, ensuring standardized spatial mapping of EEG sensors. This standardized layout is crucial in biometric systems for ensuring reproducible and reliable signal capture. Since then, different systems have developed that vary from low-density configurations (10–20 systems) to ultra-high-density layouts (5% system) [81].

The International 10–20 System is the most commonly used EEG electrode placement system, comprising around 21 to 32 electrodes spaced at 10% or 20% intervals. A typical international 10–20 system for the placement of electrodes is shown in Figure 6. Electrodes are labeled based on brain lobes, ‘F’ for frontal, ‘C’ for central, ‘P’ for parietal, ‘O’ for occipital and numbered according to their position on the left (odd), right (even), or midline (z) of the scalp. Due to its simplicity and standardized layout, it is commonly used in early-stage EEG biometric studies [81]. However, it offers limited spatial resolution and may fail to capture finer-grained neural activity patterns required for more sophisticated authentication systems.

The 10–10 system extends the 10–20 layout by placing electrodes at 10% intervals, resulting in around 64 electrode for enhanced spatial resolution. This denser configuration is well suited for EEG biometric studies employing deep learning approaches, as it enables the extraction of rich spatio-temporal features. Tasks like motor imagery and Rapid Serial Visual Presentation (RSVP) particularly benefit from the increased coverage. However, the system’s longer setup time limits its practicality for real-time or consumer-grade applications [82].

The 10–5 system provides high spatial resolution with up to 128 electrodes placed at 5% intervals. This dense configuration enables the capture of detailed neural patterns, enhancing classification accuracy and supporting deep learning models for biometrics. However, its complex setup, hardware demands, and high computational cost limit its use to laboratory-based studies rather than real-world deployments [83,84].

The 5% system uses ultra-high-density arrays with 256 or more electrodes, offering exceptional spatial resolution utilized in high-precision biometric analysis [85]. It enables detailed feature extraction and supports deep neural networks capable of identifying subtle neural patterns. However, its high cost, complex setup, and sensitivity to noise make it impractical for real-world authentication. Instead, it is primarily used in research to explore the upper performance limits of EEG-based biometric systems [86].

### 4.3. EEG Acquisition Devices

A wide range of EEG devices is available for recording brain activity, and selecting an appropriate device is particularly crucial when designing EEG-based biometric authentication systems. Since the electrical potentials generated by neuronal activity are extremely weak, typically in the range of microvolts, an ideal EEG system must not only amplify these low-amplitude signals effectively but also minimize noise, motion artifacts, and environmental interference. The overall reliability, stability, and quality of the acquired EEG data depend heavily on the hardware configuration, electrode quality, number of channels, sampling rate, and onboard signal processing capabilities of the chosen device. Broadly, EEG systems can be categorized into research-grade and consumer-grade devices based on their intended use, channel density, hardware precision, flexibility, portability, and cost [87].

*Research-grade* EEG devices, such as those manufactured by Brain Products [17], Neuroscan [38], ANT Neuro [38], and g.tec [71], are engineered for high-fidelity neurophysiological recording. They typically provide 32 to 256 channels with high sampling frequencies, low-noise amplifiers, stable impedance management, and full compatibility with standardized electrode montages (10–20, 10–10, and 5–5 systems). Such devices support configuration of reference schemes, real-time impedance monitoring, multimodal integrations (e.g., EMG, ECG, eye-tracking), and are versatile enough to support clinical diagnostics, cognitive neuroscience experiments, BCI research, and high-precision biometric investigations [88]. Their ability to capture subtle spatio-temporal neural signatures makes them particularly suitable for exploring the upper performance limits of EEG biometrics, especially in controlled laboratory environments. Despite their superior signal quality and spatial resolution, research-grade EEG systems present several practical limitations for biometric deployment. High equipment cost, lengthy setup procedures, the need for trained operators, and reduced portability can limit their applicability in everyday authentication scenarios. Furthermore, studies have shown that increasing channel density does not necessarily result in proportional improvements in biometric performance, suggesting that carefully selected electrode subsets may provide a more favorable balance between accuracy and usability.

In contrast, *consumer-grade* EEG devices, including Emotiv systems [5,89], NeuroSky headsets [38], and OpenBCI platforms [50], are designed with priorities such as portability, low cost, minimal setup time, and user comfort. These devices generally provide fewer channels (1–32 channels) and may exhibit higher levels of noise and lower sampling rates compared to research-grade systems. Despite these limitations, their increasing signal quality, wireless connectivity, dry or semi-dry electrode options, and open-source software support have made them highly appealing for real-world and practical EEG biometric authentication applications. Recent studies indicate that consumer-grade EEG systems, when combined with robust preprocessing and deep learning techniques, can deliver competitive authentication performance in practical, mobile, and short-duration usage scenarios [90]. Another important consideration is electrode technology. Research-grade systems predominantly employ wet electrodes, which generally provide lower electrode–skin impedance and higher signal quality but require conductive gel and longer preparation times. In contrast, many consumer-grade devices utilize dry or semi-dry electrodes to improve user comfort and reduce setup complexity. While these electrode technologies enhance practicality, they may be more susceptible to motion artifacts and variations in contact quality. Consequently, selecting an EEG acquisition device for biometric authentication involves balancing signal fidelity, user convenience, acquisition time, portability, and deployment cost.

**Figure 7:**
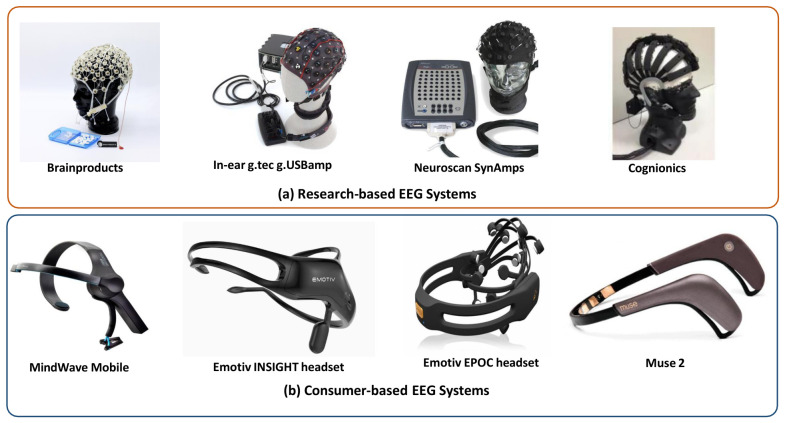
An illustration of examples of different EEG-based devices: (**a**) Research-based EEG system; (**b**) Consumer-based EEG system.

Table 5 presents a comparison of representative EEG devices based on research-grade and consumer-grade, summarizing their specifications, advantages, and limitations, while Figure 7 illustrates visual examples of both consumer and research-grade EEG hardware. This comparison highlights the trade-offs between signal quality, usability, and applicability, providing insight into selecting the most suitable device for EEG-based biometric authentication research.

## 5. Data Acquisition Protocols

For EEG acquisition, the design of the data acquisition protocol plays a pivotal role in determining the quality, reliability, and reproducibility of the recorded brain signals. Carefully structured data acquisition protocols are necessary to enhance the Signal-to-Noise Ratio (SNR) and also to minimize artifacts arising from subject movement, fatigue, or inattention. Maintaining the participant’s engagement throughout the recording session is critical to elicit stable and repeatable neural responses, which are fundamental for achieving consistent performance in biometric authentication.

**Figure 8:**
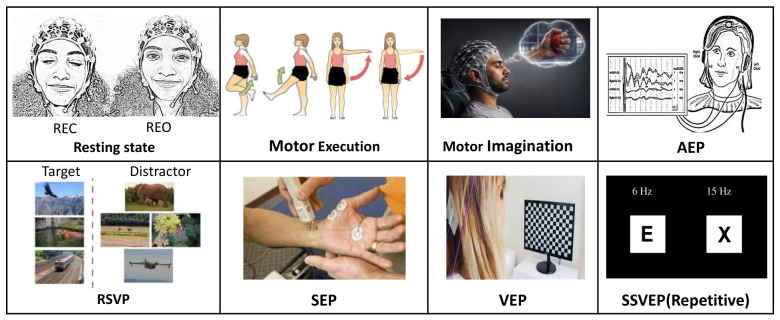
Visual representation of some data acquisition tasks employed in EEG biometric work: Resting state, motor execution, motor imagination, Auditory Evoked Potential (AEP), Rapid Serial Visual Presentation (RSVP), Somatosensory Evoked Potential (SEP), Visual Evoked Potential (VEP), and Steady-State Visual Evoked Potential (SSVEP).

**Figure 9:**
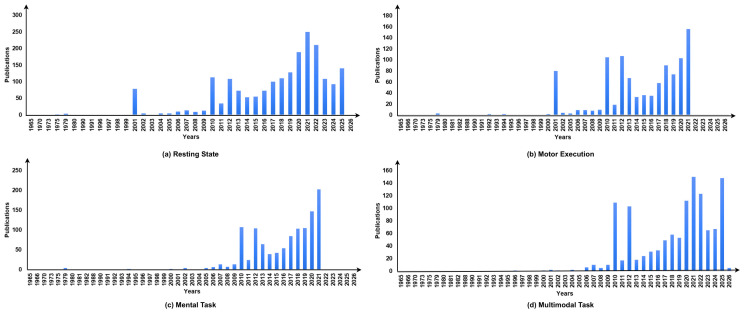
Shows the number of research publications in the area of EEG biometrics employing four different data acquisition tasks in the previous works, starting from the year 1965 to present: (**a**) Resting state; (**b**) Motor execution; (**c**) Mental task; and (**d**) Multimodal task.

**Figure 10:**
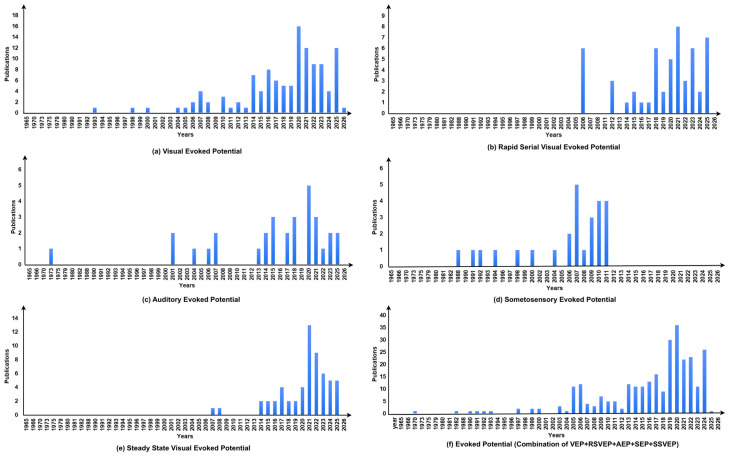
Shows the number of research publications in the area of EEG biometrics employing five different data acquisition tasks based on evoked potential in the previous works, starting from the year 1965 to present: (**a**) Visual Evoked Potential (VEP); (**b**) Rapid Serial Visual Evoked Potential (RSVEP); (**c**) Auditory Evoked Potential (AEP); (**d**) Somatosensory Evoked Potential (SEP); (**e**) Steady State Visual Evoked Potential (SSVEP); and (**f**) Evoked Potential (Combination of VEP + RSVEP + AEP + SEP + SSVEP).

This Section 5 presents key considerations for designing EEG data acquisition protocols tailored to biometric authentication. It discusses the various types of experimental paradigms employed to evoke discriminative brain responses and evaluates their relative strengths and limitations within the context of EEG biometrics. The overall objective is to provide researchers with methodological guidance for developing EEG experiments that reliably capture distinctive and individual-specific neural patterns suitable for authentication. Commonly adopted EEG acquisition paradigms for biometric applications include Resting State, Evoked Potential, Mental Task, Motor Execution, and Multimodal or Hybrid paradigms. Each of these approaches is discussed in detail in the following subsections with some illustration images shown in Figure 8. We have also shown the number of publications for each stimulus from 1965 till the present in Figure 9 and Figure 10.

### 5.1. Resting State

The resting-state data acquisition protocol captures spontaneous brain activity when the subject is not performing any task, typically under Resting-state Eyes Open (REO) or Resting-State Eyes Closed (REC) conditions. It is widely used in EEG biometrics owing to its simplicity, non-intrusiveness, and minimal setup requirements [4,36,47,54,93,94]. The recorded signals, which predominantly comprise alpha, theta, beta, and gamma oscillatory components, encode individual-specific brain dynamics valuable for user authentication. Features such as power spectral density, entropy, and functional connectivity have proven effectiveness in distinguishing individuals with high accuracy and low subject involvement [29,90,95]. While its performance may vary with mental states and external noise, the resting-state data acquisition protocol remains ideal for passive or continuous biometric systems due to its ease of deployment and temporal stability.

### 5.2. Evoked Potential

Evoked Potential (EP) protocols are the brain responses recorded in response to specific external stimuli, including visual, auditory, and somatosensory inputs. The brain’s reaction to these stimuli is captured in the form of Event-Related Potentials (ERPs), which are time-locked neural responses embedded in the EEG signal. These ERPs reflect sensory, cognitive, and motor processing, and exhibit individual-specific variations in terms of latency, amplitude, and topographical distribution, making them suitable for biometric applications [20,96]. Based on the type of stimulus used, EP paradigms are categorized into: Visual Evoked Potentials (VEPs), Auditory Evoked Potentials (AEPs), Steady-State Visual Evoked Potentials (SSVEPs), Somatosensory Evoked Potentials (SEPs) [20,96,97]. These modalities offer varied cognitive load and biometric discriminability, which have been significantly exploited for biometric user authentication.

*Visual Evoked Potentials (VEPs)* are a class of Event-Related Potentials (ERPs) elicited by the brain in response to visual stimuli such as flashing lights, checkerboard patterns, or images [17,41,46,50,98,99]. In VEP-based EEG biometric systems, subjects are presented with controlled visual cues while their EEG is recorded [29]. These stimuli generate time-locked, stimulus-specific responses originating primarily from the occipital and parietal regions of the brain [99]. The resulting waveforms are highly structured and reproducible, exhibiting individual-specific variations in parameters such as latency, amplitude, and morphology [29]. The effectiveness and robustness of VEP-based systems have been well demonstrated in earlier studies, showing stability across sessions and resilience to spoofing attacks in EEG biometrics [29].

*Steady-State Visual Evoked Potentials (SSVEPs)* are a widely used type of VEP, produced by repetitive visual stimuli flickering at constant frequencies, typically in the range of 5 Hz to 60 Hz [100]. SSVEPs generate sinusoidal EEG responses at the stimulus frequency and its harmonics, with individual-specific variability in amplitude, phase, and spectral content, and these attributes are widely used for identity recognition [1,17,38]. These signals are known for their high Signal-to-Noise Ratio (SNR) and temporal stability, contributing to the robustness of SSVEP-based biometric systems [101].

Unlike transient VEP paradigms that are generated by isolated visual events, SSVEP protocols rely on continuous periodic visual stimulation and sustained user attention toward flickering stimuli. During SSVEP recording, subjects are required to continuously focus on the flickering stimulus, which helps standardize brain activity and reduce the influence of environmental or internal cognitive fluctuations [102]. However, SSVEP systems require strict synchronization between the visual stimulation hardware and EEG acquisition system to accurately capture frequency-specific neural responses and harmonics.

However, prolonged exposure to such stimuli may lead to visual fatigue or discomfort, particularly in sensitive users [46]. Further, dependence on specialized stimulation hardware and controlled illumination conditions may limit portability and large-scale practical deployment of SSVEP-based authentication systems.

*Rapid Serial Visual Presentation (RSVP)* is again a VEP-based EEG acquisition protocol where a rapid stream of low-probability target stimuli (e.g., self or imposter face [3]) is interspersed with high-probability non-target stimuli such as letters, famous face, or neutral images [39]. RSVP commonly elicits the P300 (P3) ERP component, associated with decision-making and cognitive evaluation [32]. The P300 is an endogenous potential with a latency of 250–500 ms, typically maximal at the Cz electrode. Peaks within 300–400 ms are considered early P3, while 380–440 ms are considered late P3. This component is typically evoked using the oddball paradigm, which shares the same design as RSVP.

Unlike SSVEP paradigms, which typically require users to fixate their gaze on a flickering visual stimulus at a specific frequency, RSVP presents stimuli sequentially at a single spatial location. Because all stimuli in RSVP are shown at the same location, the user does not need to shift gaze or perform intentional eye movements to select a target. This makes RSVP particularly suitable for individuals with motor impairments. However, it demands sustained attention, which can lead to mental fatigue and reduced accuracy over time. Further, the rapid pace may cause users to miss targets, and its visual nature limits usability for low-vision or blind individuals.

*Auditory Evoked Potential (AEP)* is another ERP generated when individuals respond to emotionally varied auditory stimuli, such as happy, sad, and neutral tones [40]. AEPs are commonly used to study the neural transmission of auditory signals from the acoustic nerve to the auditory cortex. Earlier research has shown that theta and alpha oscillations in the central and occipital scalp regions are correlated with the processing of auditory stimuli [103]. The characteristics of AEPs can vary significantly based on age, hearing ability, neurological conditions, and cognitive state [40]. Individuals with hearing impairments or auditory processing disorders often exhibit weak or inconsistent AEP features, which may lead to variations in responses across sessions and reduce the reliability of the AEP-based data acquisition protocol for biometric systems.

*Somatosensory Evoked Potentials (SEPs)* [46] are brain responses obtained by stimulating peripheral sensory nerves through electrical, mechanical, or vibrotactile stimuli. These responses are recorded via EEG electrodes placed over the somatosensory cortex. SEPs have been extensively used in neurological diagnostics, Brain–Computer Interface (BCI) systems, and more recently, in biometric authentication [32]. The primary strength of SEP-based biometrics lies in their individual-specific neural signatures, which are difficult to forge, offering greater security [104]. Further, SEP patterns are relatively stable over time, minimizing the impact of age-related biometric changes [32]. However, SEPs suffer from low Signal-to-Noise Ratio (SNR) and are prone to artifacts from eye blinks, muscle movement, and environmental noise. SEP acquisition is also time-consuming, often requiring multiple trials, and electrical stimulation may cause user discomfort, limiting real-world usability [105].

### 5.3. Motor Execution

Motor-based EEG biometrics utilizes neural signals generated during actual physical movement to verify identity. These systems primarily rely on Motor-Related Cortical Potentials (MRCPs) and Movement-Related Evoked Potentials (MREPs). MRCPs are slow brain waves that appear before voluntary movement, while MREPs reflect EEG changes during motion execution. Particularly, those originating from the motor cortex exhibit subject-specific characteristics that can be distinguished across individuals. These characteristics are reflected in three primary signal properties in terms of amplitude, latency, and waveform shape [106,107].

Motor tasks such as hand and foot movements provide rich EEG patterns for recognition and offer higher robustness in real-world settings [29]. However, movement introduces signal artifacts and the approach may not be suitable for individuals with motor impairments. Repeated physical actions can also cause fatigue, reducing user comfort and system usability [108,109]. Despite these limitations, motor-based EEG offers a secure and active authentication method.

### 5.4. Mental Imagery Task

Mental task-based EEG biometrics involve subjects performing imagined or cognitive activities such as speech imagination [110], mental counting [110], arithmetic operations [5,111], figure rotation [112], and motor imagery [36,113]. Among these, motor imagery, where the subject imagines moving a limb or tongue without actual movement, is the most commonly used. Studies have shown that imagined movements activate similar brain regions as physical execution, including the premotor cortex, supplementary motor area, parietal regions, basal ganglia, and cerebellum [114].

In comparison to motor execution paradigms, motor imagery protocols eliminate overt physical movement and therefore reduce movement-related artifacts and muscular interference in EEG recordings. This makes motor imagery particularly attractive for users with physical disabilities and for applications requiring minimal physical interaction. However, motor imagery generally demands higher cognitive effort, longer user training, and sustained concentration, which may increase mental fatigue and session-to-session variability. In contrast, motor execution often produces stronger and more stable neural responses due to actual movement generation, but it introduces additional motion artifacts and may reduce practicality for users with motor impairments. Therefore, both paradigms present distinct trade-offs in terms of signal quality, user comfort, robustness, and real-world deployment feasibility in EEG biometric systems.

This approach is particularly promising for secure authentication, including for users with physical impairments. Compared to other acquisition protocols, mental tasks offer higher inter-subject variability and cognitive engagement, leading to more distinctive neural patterns and improved Signal-to-Noise Ratio (SNR) [115]. However, the sustained cognitive effort required may cause user fatigue, affecting consistency across sessions.

### 5.5. Multimodal or Composite Tasks

Multimodal or Composite EEG biometric approaches leverage signals generated from multiple evoked potential paradigms, such as visual, auditory, motor, and cognitive tasks, to improve the robustness, accuracy, and security of user authentication systems. Instead of relying on a single EEG modality, these systems combine diverse neural responses evoked by different tasks, such as Visual Evoked Potentials (VEPs), Auditory Evoked Potentials (AEPs), Motor Imagery (MI), and mental arithmetic, thereby capturing a more comprehensive and discriminative representation of an individual’s brain activity. This multimodal fusion has been shown to significantly increase resistance to spoofing attacks, reduce intra-subject variability, and improve inter-subject classification performance [29]. Some studies have also integrated behavioral or emotional EEG responses with task-evoked potentials, further boosting biometric reliability [116]. Feature-level or decision-level fusion strategies are commonly employed to combine these signals effectively in EEG biometrics [116].

Despite their advantages, composite EEG systems face challenges such as increased computational demands due to complex signal processing and classification models. Multi-step tasks can cause user fatigue, affecting signal quality and consistency. Environmental sensitivity and session-to-session variability further impact system reliability, necessitating robust calibration [117]. Additionally, the requirement for high-density EEG setups and extended recordings limits their practical deployment.

Although significant progress has been achieved in EEG biometric authentication using diverse acquisition protocols, each paradigm presents inherent trade-offs in terms of usability, robustness, computational complexity, and authentication reliability. Resting-state protocols are simple to deploy and require minimal user effort; however, they are highly sensitive to variations in mental state, drowsiness, stress, and environmental noise, which may reduce permanence across sessions. In contrast, evoked-potential-based paradigms such as VEP and SSVEP generally provide higher Signal-to-Noise Ratio (SNR) and more reproducible neural responses due to controlled external stimulation. Nevertheless, these systems require precise stimulus presentation hardware and may introduce user discomfort or visual fatigue during prolonged usage.

Motor execution and motor imagery paradigms provide richer subject-specific neural patterns and stronger cognitive engagement, improving discriminative capability in many studies. However, these approaches are often associated with higher cognitive workload, increased training requirements, and reduced practicality for users with motor impairments or limited attention span. Similarly, mental task paradigms can improve inter-subject separability but frequently suffer from session-to-session variability caused by fluctuating cognitive and emotional states.

Multimodal EEG biometric systems attempt to overcome the limitations of single-paradigm approaches by combining complementary neural responses. While such systems often report improved robustness and classification performance, these gains are achieved at the cost of increased computational complexity, longer acquisition time, more sophisticated preprocessing pipelines, and higher user burden. In practical real-world deployments, these additional requirements may limit scalability, portability, and user acceptance.

Furthermore, many existing studies report high authentication accuracies under controlled laboratory conditions using limited datasets and short-term recordings. However, the long-term permanence, cross-session generalization, and robustness of EEG biometrics under real-world operational environments remain open research challenges. Variability introduced by electrode placement, physiological conditions, emotional state, fatigue, and hardware differences continues to affect reproducibility and large-scale deployment feasibility.

Therefore, despite the promising security and uniqueness offered by EEG biometrics, future research must focus not only on improving classification accuracy but also on enhancing usability, interpretability, long-term stability, computational efficiency, and practical deployment adaptability.

Figure 9 shows the number of publications in earlier works, starting from the year 1965 to the present, based on four EEG data acquisition tasks, which include resting state, motor execution, mental task, and multimodal task. Figure 10 shows the number of publications in earlier works, starting from the year 1965 to the present, based on five EEG data acquisition tasks related to evoked potentials. These include Visual Evoked Potential, Rapid Serial Visual Presentation, Auditory Evoked Potential, Somatosensory Evoked Potential, and Steady-state Visual Evoked Potential.

**Figure 11:**
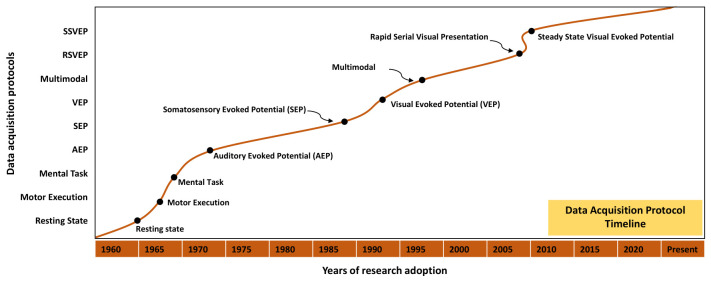
Timeline illustrating the adoption of data acquisition protocols in EEG biometric research work.

**Figure 12:**
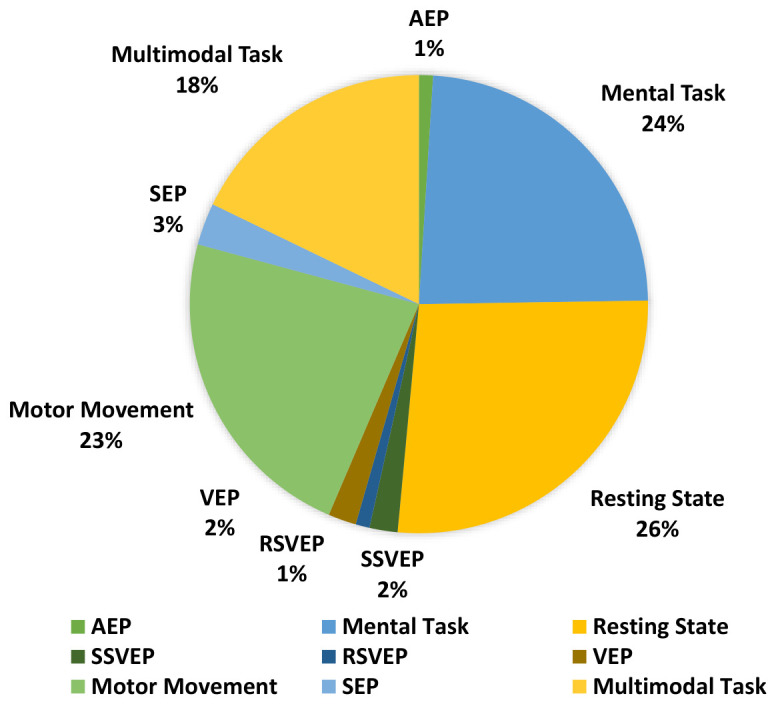
This figure represents the percentage of the number of research papers published for different EEG data acquisition protocols that are available from 1965 to present for EEG biometrics.

**Figure 13:**
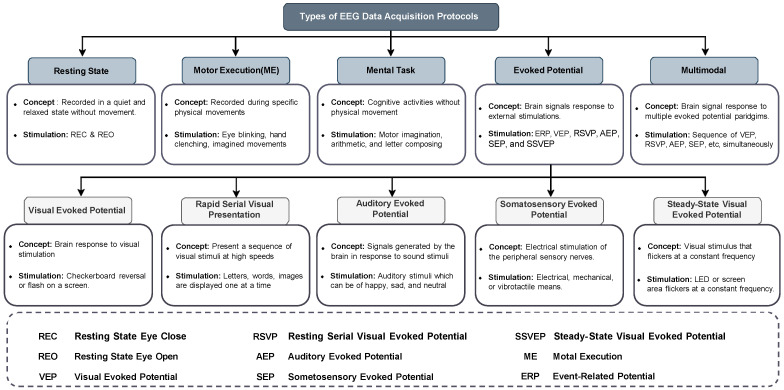
Illustration of different data acquisition protocols summarizing their basic concepts and stimulations.

Figure 11 illustrates the timeline showing the adoption and evolution of various EEG acquisition protocols in EEG biometric research. The dashed lines indicate periods of significant advancement in specific EEG biometric acquisition paradigms. Figure 12 shows the percentage distribution of research papers published under different EEG biometric acquisition protocols from the emergence of EEG biometric authentication research to the present. Although EEG recording technology originated in the early twentieth century following the pioneering work of Hans Berger, EEG-based biometric authentication emerged much later with advances in digital signal processing, pattern recognition, and machine learning techniques. Figure 13 presents a comparison of different EEG data acquisition protocols, summarizing their fundamental concepts and stimulation mechanisms.

## 6. EEG Database

This section outlines several publicly available and proprietary EEG repositories relevant to biometric research. These datasets provide access to recent developments and serve as a valuable foundation for advancing EEG-based biometric authentication systems. Table 6 presents a detailed description of publicly and proprietary EEG databases employed in biometric research.

### 6.1. Publicly Available Database

#### 6.1.1. Physionet EEG Motor Movement Imagery (MMI) Dataset

The physionet MMI [118] is the most popular database used in many biometric research works. The database was collected using the 64-channel BCI2000 acquisition system (http://www.bci2000.org (accessed on 15 June 2026)) configured according to the international 10-10 electrode montage. EEG signals were recorded from 109 subjects while they performed a series of motor movements and imagery tasks, sampled at 160 Hz. Each participant performed 14 experimental tasks; two tasks were baseline runs, consisting of one-minute recordings each for eye-open and eye-closed conditions. The remaining 12 tasks were divided into three repetitions of four different tasks. Those four tasks include: (1) Left or right fist movement (subjects opened and closed the left or right fist when a target appeared on the corresponding side of the screen); (2) Left or right fist imagery (subjects imagined opening and closing the left or right fist according to target position); (3) Both fists movement; (4) Both fists imagery. This dataset was originally designed for Brain–Computer Interface (BCI) research but due to its comprehensive motor and imagery components, this dataset is now widely used for EEG biometric studies [24,36,119,120]. The details of the dataset, including subject information, experimental setup, and raw EEG files, are publicly available at: https://physionet.org/content/eegmmidb/1.0.0/ (accessed on 15 June 2026).

#### 6.1.2. The Swartz Center for Computational Neuroscience Foundation (SCCN) Dataset

SCCN [121] hosts a range of publicly accessible EEG data repositories to promote reproducible research in Electrophysiology. One of the early and widely used publicly shared SCCN datasets includes 32-channel EEG recordings collected from 14 healthy subjects performing rapid visual categorization tasks in a Go/No-Go paradigm. The EEG signals were recorded using a SynAmps acquisition system (https://compumedicsneuroscan.com/product/synamps-rt-64-channel-eeg-erp-ep-amplifier/ accessed on 15 June 2026) with electrodes positioned according to the standard International 10–20 configuration and sampled at 1000 Hz, thereby providing high temporal resolution suitable for detailed time–frequency analysis and Event-Related Potential (ERP) investigations. The dataset consists of four experimental series, each containing 100 images. During each series, participants were required to execute a Go response by lifting their finger from a response button within 1000 ms whenever a target stimulus (an animal image) appeared. A response latency exceeding 1000 ms was categorized as a No-Go response. In contrast, when non-target images (such as landscapes, cities, fruits, vegetables, or trees) were presented, participants were instructed to maintain the button press for at least 1000 ms, thereby suppressing motor response. This design enables clear differentiation between motor execution (Go) and response inhibition (No-Go) conditions, facilitating analysis of Event-Related Potentials and cognitive control mechanisms. Like the Physionet MMI dataset, SCCN EEG recordings were primarily created for neuroscience research, BCI studies, cognitive and motor task analysis, and EEG modeling research but because the EEG data collected in this dataset still contain person-specific patterns, even though unintended, it is still used for biometrics research [122,123,124]. The dataset and additional documentation can be publicly accessible at: https://sccn.ucsd.edu/~arno/fam2data/publicly_available_EEG_data.asv (accessed on 15 June 2026).

#### 6.1.3. BCI Competition III Dataset

The BCI Competition III Dataset [125] is one of a series of benchmark EEG datasets released as part of the Brain–Computer Interface (BCI) Competitions, particularly for evaluating signal processing algorithms in motor imagery and Event-Related Potential (ERP) paradigms and later exploited for EEG biometrics. The BCI Competition III collection consists of multiple datasets contributed by different research laboratories, each representing distinct experimental paradigms and signal characteristics. Among the subsets of datasets, the most commonly utilized dataset is Dataset IIIa, which was recorded using 60 channels arranged according to the international 10–20 system, at a sampling rate of 250 Hz. The signals were band-pass filtered between 1 Hz and 50 Hz, and a notch filter was applied to eliminate power line interference. This dataset includes recordings from three subjects performing motor imagery tasks involving the left hand, right hand, foot, and tongue, with each subject completing 60 trials per task. Another notable sub-dataset, Dataset V, was collected using a Biosemi EEG acquisition system with 32 electrodes according to the international 10–20 system and a sampling rate of 512 Hz. It comprises four non-feedback sessions conducted on the same day, where participants performed three distinct mental tasks that include left-hand motor imagery, right-hand motor imagery, and word generation. The diversity of the BCI Competition III datasets makes them a valuable resource for testing various signal processing methods and classification algorithms. For EEG biometrics research, these datasets are particularly utilized for evaluating subject-specific neural patterns, thereby supporting the development of reliable authentication systems [20,29,126,127]. The datasets are publicly available at: http://www.bbci.de/competition/iii/ (accessed on 15 June 2026).

#### 6.1.4. BCI Competition IV Datasets

The BCI Competition IV datasets [128], released in 2008, serve as a benchmark for evaluating EEG signal under realistic and challenging conditions in motor imagery for BCI applications compared to previous BCI Competition III dataset. The competition contains multiple independent datasets (1, 2a, 2b, 3, and 4) collected using different paradigms, subjects, electrode configurations, and recording systems. Dataset 2a is frequently utilized and contains EEG recordings from nine subjects performing four-class motor imagery tasks (left hand, right hand, foot, and tongue movements). Data were recorded using a g.USBamp biosignal amplifier equipped with 22 EEG channels and 3 monopolar Electrooculography (EOG) channels placed according to the international 10–20 standard and with a sampling rate of 250 Hz. Each subject completed two sessions of recording, with each session comprising six runs interspersed with short breaks. Another subset, Dataset 2b of the BCI Competition IV, includes recordings from nine subjects acquired using a g.USBamp biosignal amplifier with 22 EEG channels, 3 EOG channel sampled at 250 Hz, and participants performing motor imagery tasks (left hand and right hand) in five sessions. This experiment employs 120 runs per session for each individual subject. The BCI Competition IV Dataset is particularly valuable for research focused on minimal electrode setups and computational efficiency in EEG biometrics [20,29,126,127,129,130]. The datasets are publicly available at: https://www.bbci.de/competition/iv/ (accessed on 15 June 2026).

#### 6.1.5. UCI EEG Database Dataset

This database contains EEG recordings from 122 subjects, comprising 45 healthy control participants and 77 individuals diagnosed with alcoholism [127]. This dataset was originally generated to study the differences in brain activity patterns associated with alcoholism and healthy controls and repurposed for use in biometrics. The data were collected using a 64-channel NeuroScan EEG recording system (SynAmps amplifier), with electrodes positioned according to the international 10–20 standards and sampled at 256 Hz. During data acquisition, subjects were presented with visual stimuli consisting of object images from the Snodgrass and Vanderwart picture set, with multiple repeated trials conducted per subject to capture event-related neural responses. This inherent variability allows researchers to evaluate the robustness of EEG signal processing algorithms under differing data availability conditions in biometrics [24,30,129]. The dataset has been extensively used in studies on Event-Related Potentials, and neural response variability. The dataset is publicly accessible at: https://archive.ics.uci.edu/dataset/121/eeg+database (accessed on 15 June 2026).

#### 6.1.6. DEAP Dataset

The DEAP Dataset [131] was originally developed for emotion analysis based on EEG, peripheral physiological signals, and video data. Although it was not designed for biometric applications, it has been widely adopted in EEG research for evaluating signal processing and machine learning methods. In some studies, it has also been used for EEG-based biometric analysis by exploiting inter-subject variability in neural responses [132].

The EEG and physiological signals were recorded from 32 healthy subjects using a 32-channel BioSemi ActiveTwo EEG system with active electrodes, sampled at 512 Hz. Electrode placement followed the international 10–20 system, providing adequate scalp coverage for emotion-related neural activity.

During the experiment, participants viewed 40 one-minute music video clips selected to elicit a broad spectrum of emotional states. Each trial consisted of a short baseline period followed by a video presentation, allowing baseline correction and event-related analysis. After viewing each video, subjects provided self-assessment ratings on a discrete scale for arousal, valence, dominance, like/dislike, and familiarity, enabling both regression and classification-based emotion modeling. For 22 out of the 32 subjects, frontal face videos were also recorded using a standard video camera to capture facial expressions, facilitating multimodal emotion analysis by correlating EEG and physiological responses with visual affective cues. The dataset has been extensively used for research in affective computing, multimodal fusion, and EEG-based emotion recognition, and remains a standard benchmark for evaluating signal processing and machine learning techniques [15,133,134]. The dataset, along with comprehensive documentation and preprocessed versions, is publicly available at: https://www.kaggle.com/datasets/harshilgupta28/deap-dataset?resource=download(accessed on 15 June 2026).

#### 6.1.7. Zurich Cognitive Language Processing Corpus (ZuCo)

The Zurich Cognitive Language Processing Corpus (ZuCo) [135] is a publicly available multimodal dataset that was originally developed for studying cognitive and linguistic processing during natural reading using simultaneous EEG and eye-tracking recordings. Although not designed for biometric applications, it has been occasionally used in EEG research [127,134] to explore cross-subject variability and machine learning-based subject modeling in controlled experimental settings. The initial release, ZuCo 1.0, comprises data from 12 native English speakers, and later ZuCo 2.0 expands participation to 18 subjects, each contributing approximately 4–6 h of reading data. ZuCo 1.0 includes recordings of 21,629 words across 1,107 sentences, covering three main tasks: (1) Normal reading (sentiment analysis) using text from the Stanford Sentiment Treebank; (2) Relation extraction from Wikipedia-based datasets; and (3) Task-specific reading experiment where participants identified semantic relations. These EEG signals were recorded using a 64-channel Brain Products actiCHamp EEG system with active electrodes at a sampling rate of 500 Hz. Researchers can access ZuCo 1.0 through https://www.research-collection.ethz.ch/handle/20.500.11850/313239 (accessed on 15 June 2026) and ZuCo 2.0 from the https://osf.io/q3zws/ (accessed on 15 June 2026).

#### 6.1.8. Penn Electrophysiology of Encoding and Retrieval Study (PEERS) Dataset

The PEERS Dataset [136], provided by the University of Pennsylvania, is a large-scale EEG dataset collected between 2010 and 2020 to study the neural correlates of memory processes, particularly during word memorization tasks. Participants were presented with sequences of words as stimuli, and EEG recordings were synchronized with stimulus presentation using event markers, enabling precise segmentation into individual EEG samples. The dataset was recorded at a sampling rate of 500 Hz and contains EEG data from 345 subjects across 6007+ multi-session recordings, making it highly suitable for analyzing inter-session variability in EEG biometrics. The participants ranged from 17 to 85 years, with an average age of 26.98±15.86 years. Data acquisition was performed using three medical-grade high-density EEG systems, including 128- and 129-channel headsets, which enhances the dataset’s practical relevance by incorporating device-level variability. Owing to its large subject pool, multi-session structure, and heterogeneous acquisition devices, the PEERS Dataset is highly valuable for developing robust real-world EEG biometric systems. The dataset, along with comprehensive documentation, is publicly available at: https://openneuro.org/datasets/ds004395/versions/2.0.0 (accessed on 15 June 2026).

### 6.2. Proprietary Available Database

#### 6.2.1. SEED-IV Database

SEED Emotion Database Version IV [137] is an advanced extension of the SEED (SJTU Emotion EEG Dataset) series, developed by the BCI Laboratory at Shanghai Jiao Tong University (SJTU) for emotion recognition research. The database contains EEG recordings from 15 subjects, each participating in three experimental sessions conducted on different days to capture intra-subject variability and emotional stability over time. During each session, participants watched emotionally evocative movie clips designed to elicit positive, neutral, and negative emotional states. EEG data were collected using a NeuroScan EEG recording system (SynAmps amplifier) equipped with 64 channels placed according to the international 10–20 system and at a sampling rate of 1000 Hz. Although the dataset was originally designed for emotion recognition, it has also been explored in EEG research for studying cross-condition variability [138,139,140] and robustness of signal representations across different affective states which makes it relevant for assessing the robustness of EEG feature representations under non-stationary cognitive and affective states. More information about the dataset is available at the SJTU BCI Laboratory website: https://bcmi.sjtu.edu.cn/home/seed/ (accessed on 15 June 2026).

#### 6.2.2. M3CV Database

The Multi-subject, Multi-session, and Multi-task Cognitive and Visual EEG Database (M3CV) [46] is a comprehensive multi-subject, multi-session, and multi-task dataset specifically developed for EEG-based biometric research. It comprises recordings from 106 young adult participants carried out in two sessions. This longitudinal structure enables investigations into the temporal stability and cross-session robustness of EEG-based authentication systems. All EEG signals were recorded using a 64-channel Brain Products actiCHamp EEG system, arranged according to the international 10–20 electrode placement standard, and sampled at 250 Hz. The M3CV EEG database comprises multiple distinct experimental paradigms designed to comprehensively capture various aspects of brain activity. These tasks were based on visual categorization, target detection, attention-based tasks, and cognitive load experiments. The M3CV dataset provides a rich and diverse framework for studying neural distinctiveness, intra-subject variability, and task-dependent EEG dynamics, making it one of the most comprehensive publicly available resources for EEG-based biometric authentication and neuroscience research [46]. Detailed documentation of the dataset is accessible at: https://aistudio.baidu.com/aistudio/datasetdetail/151025/0 (accessed on 15 June 2026).

#### 6.2.3. Brain Wave-Based EEG Database (BED)

The BED [5] was developed to support biometric person authentication research. The dataset comprises recordings from 25 healthy adult participants, collected across three sessions on separate days to allow investigations of template ageing and intra-subject variability. EEG signals were recorded using a Muse 2 headband (InteraXon Inc., Toronto, Canada), a 4-channel frontal and temporal consumer EEG device, with a sampling rate of 256 Hz. Data acquisition incorporated twelve types of stimuli, including affective stimuli, resting-state conditions (eyes open and eyes closed), mathematical computation tasks, flashing Visual Evoked Potentials (VEPs) at 3, 5, 7, and 10 Hz, and checkerboard VEPs at the same frequency range. This multi-session, multi-stimulus dataset enables researchers to study the effects of emotional states on EEG signals, as well as EEG-based biometric template stability over time [5,123,141]. Detailed information on the BED dataset is available at: https://zenodo.org/records/4309472 (accessed on 15 June 2026).

#### 6.2.4. BIOMEX-DB

The Biometric Of Multi-modal Experiments DataBase (BIOMEX-DB) [133] is a multimodal dataset comprising synchronously recorded EEG, voice, and video signals from 51 participants (25 females and 26 males). The EEG data were acquired using a 14-channel Emotiv Epoc+ headset, a consumer-grade wireless EEG device with electrodes positioned according to the international 10–20 system, providing coverage of frontal, temporal, parietal, and occipital regions. The signals were recorded at a sampling rate of 128 Hz. During the experiment, participants pronounced single digits in Spanish, producing a total of 7140 EEG recordings, corresponding to 140 samples per subject. The simultaneous acquisition of EEG, voice, and video data enables the exploration of cross-modal correlations, fusion strategies, and multimodal biometric authentication [133,142,143]. The dataset is particularly valuable for investigating EEG-based user authentication, speech-related neural patterns, and affective or cognitive state decoding in controlled conditions. Detailed information and access to the BIOMEX-DB dataset can be found at: https://data.mendeley.com/datasets/s7chktmb6x/1 (accessed on 15 June 2026).

It is important to note from this section that the EEG datasets used in biometric research can be broadly categorized into two groups: (i) datasets originally designed for Brain–Computer Interface (BCI), clinical, cognitive, or affective neuroscience studies, and (ii) datasets specifically intended for identity-related or biometric evaluation, which are relatively rare in EEG research. Benchmark datasets such as the BCI Competition series were originally developed for evaluating EEG decoding algorithms in motor imagery and related BCI tasks, while datasets such as DEAP, SEED, PEERS, ZuCo, and UCI EEG were primarily designed for emotion recognition, cognitive processing, or clinical investigations. Although these datasets are widely adopted in EEG-based biometric studies due to their availability, multi-subject recordings, and controlled experimental settings, their use in authentication research is inherently exploratory. In particular, limitations such as small subject populations in some datasets, task-induced variability, lack of identity-centric acquisition protocols, and potential domain mismatch should be considered when interpreting biometric performance results. Therefore, conclusions drawn from such datasets should be viewed in the context of experimental evaluation rather than real-world biometric deployment.

## 7. EEG Data Preprocessing

Once EEG signals are acquired through specifically designed experimental tasks, the raw data often contain various forms of noise and artifacts [144]. These unwanted interferences may stem from ocular movements, muscle activity, power line interference, or limitations inherent to the recording hardware [145]. To ensure that subsequent feature extraction accurately captures the subject’s underlying neural activity, a critical preprocessing stage is employed. This step is essential for enhancing signal quality, suppressing artifacts, and standardizing data formats, thereby laying a robust foundation for reliable EEG-based biometric authentication [20].

Typical preprocessing procedures include the application of band-pass and notch filters to isolate relevant frequency bands and eliminate line noise, baseline drift correction to stabilize signal offsets, and advanced artifact removal techniques such as Independent Component Analysis (ICA) and wavelet-based denoising [146]. The choice of preprocessing pipeline can vary substantially across studies, influenced by factors such as the experimental paradigm, recording hardware specifications, signal quality, and the intended application domain [147]. A summary of commonly adopted preprocessing approaches is provided in Table 7 based on which we have functionally categorized these techniques into four main groups, which are signal conditioning, artifact handling, referencing, and segmentation [148].

### 7.1. Signal Conditioning

Signal conditioning enhances the quality of raw EEG data through several preprocessing techniques, such as baseline correction [3,4], downsampling, and filtering, to enhance the Signal-to-Noise Ratio (SNR) [3,4,47,71]. These preprocessing steps are critical to ensure the extraction of stable, subject-specific neural features while minimizing intra-subject variability caused by noise, drift, and physiological artifacts.

EEG signals often exhibit low-frequency drifts caused by head movements, cable shifts, or perspiration, which can obscure true neural responses. *Baseline correction* is a common method, where a stimulus-free segment is used to center the signal. Typically, the mean of the baseline period is subtracted from post-stimulus data to stabilize the signal. For more complex drift patterns, polynomial curve fitting methods such as sixth-degree fitting have also been employed to model and remove long-term drifts in the EEG signals [42].

Another essential step is *downsampling*, which reduces the EEG sampling rate to lower computational demands [47], making it particularly useful in real-time or portable biometric systems. To prevent aliasing, a low-pass filter is applied beforehand to remove frequencies beyond the new Nyquist limit [161].

To enhance SNR in EEG recordings, various filtering techniques are employed. *Laplacian filters* enhance spatial resolution by minimizing the influence of neighboring electrodes. *Notch filters* (typically at 50/60 Hz) are used to suppress power line interference, while band-pass filters (e.g., 1–40 Hz Butterworth filter) exclude irrelevant low- and high-frequency noise [36,71,82,162].

In addition, *Moving Average Filters (MAF)* are applied to smooth abrupt temporal fluctuations, whereas *Finite Impulse Response (FIR) filters*, especially those designed using Hamming windows, are often paired with Independent Component Analysis (ICA) to effectively remove ocular and muscular artifact [50]. *High-pass Filters (HPF)* [163] are utilized to eliminate slow drifts, while *Chebyshev filters* [3,150] offer sharper roll-offs for precise frequency isolation. Collectively, these signal conditioning techniques ensure that the neural signals used for biometric authentication are both clean and distinctive.

Although conventional filtering and conditioning methods remain widely adopted, their effectiveness depends strongly on the recording environment and biometric paradigm. Band-pass and notch filters are highly effective for suppressing environmental and physiological noise in controlled laboratory recordings; however, aggressive filtering [161] may also remove subject-specific neural characteristics, reducing discriminative information. Similarly, downsampling improves computational efficiency and supports real-time deployment, but excessive reduction in sampling frequency can lead to loss of high-frequency biometric patterns. Studies further suggest that spatial filtering approaches such as Laplacian filtering improve local feature separability in high-density EEG systems, whereas their benefit becomes limited in low-channel wearable devices. These observations indicate that preprocessing pipelines must balance noise suppression, computational complexity, and preservation of subject-specific neural information.

### 7.2. Artifact Handling

Artifact handling is an essential step in EEG biometric systems to preserve signal fidelity, especially when dealing with physiological or environmental artifacts such as eye blinks, muscle activity, cardiac signals, and ambient noise [164]. The choice of artifact removal technique is often influenced by the type of stimulus used for the experimental paradigm employed in the biometric data acquisition protocol.

Among the most widely adopted techniques, *Independent Component Analysis (ICA)* [17,100] is particularly effective in resting-state or task-based cognitive paradigms, where EEG signals are typically longer and unsynchronized to specific events. ICA decomposes EEG signals into statistically independent components, separating artifacts by minimizing mutual information or maximizing non-Gaussianity. The artifact-related components (e.g., eye blinks, EMG) can then be manually or automatically rejected before signal reconstruction [17,165]. ICA-based methods generally perform well in multichannel resting-state and cognitive EEG recordings, but their performance degrades in low-density or short-duration recordings where reliable component separation becomes difficult.

*Artifact Subspace Reconstruction (ASR)* is another robust approach, particularly suited for naturalistic and continuous stimuli, such as during multimedia exposure (e.g., music or video) or long emotional state monitoring, where sudden burst artifacts occur intermittently. ASR automatically detects and suppresses transient, high-amplitude, non-stationary artifacts by comparing signal deviations against a statistically derived reference covariance matrix from clean EEG segments [40]. However, this method generally requires multichannel recordings, making it less applicable to low-density or wearable EEG systems.

*Wavelet transform*-based denoising, particularly the Discrete Wavelet Transform (DWT) [133], has demonstrated strong performance across various paradigms, including Event-Related Potentials (ERPs), emotional EEG, and resting-state recordings. Wavelet-based methods decompose EEG into multi-scale time-frequency components, enabling selective removal of noise or transient distortions while preserving cognitive and emotional neural signatures [133]. However, their effectiveness depends heavily on wavelet selection and decomposition parameters.

*Adaptive filtering* techniques are commonly utilized in controlled experimental paradigms, such as Auditory or Visual Evoked Potential studies, where reference signals like EOG (for eye movements) or EMG (for muscle noise) are available and can be linearly regressed out from the EEG signal [166] but their applicability becomes limited in unconstrained or wearable acquisition environments. Similarly, regression-based artifact correction performs effectively when stimuli are structured and repeatable, allowing for the statistical modeling and subtraction of artifact contributions without distorting the underlying neural activity [167].

### 7.3. Referencing

Referencing [51,54] is a fundamental step in the EEG preprocessing stage that involves redefining the voltage baseline of each EEG channel by subtracting a common reference signal. Since EEG inherently measures potential differences between electrodes, the choice of reference profoundly influences signal interpretation, spatial resolution, and the quality of extracted features [168]. The selection of an appropriate reference often depends on the experimental paradigm and stimulus type. In stimulus-evoked paradigms, such as auditory (e.g., P300) or Visual Evoked Potentials (VEPs), references like linked mastoids or the Cz (central electrode) are typically employed. These configurations preserve event-related potentials localized near the midline, facilitating the detection of consistent, stimulus-locked neural responses [169] but may introduce spatial bias that limits generalization across sessions and recording setups. These observations indicate that no single referencing strategy is universally optimal, and the effectiveness of a reference scheme depends strongly on the acquisition paradigm, electrode density, and target feature domain.

Conversely, in resting-state or emotion-driven paradigms, where explicit event markers are absent, spatially neutral referencing methods such as the Common Average Reference (CAR) [151,170] or the Reference Electrode Standardization Technique (REST) [171] are preferred. However, CAR may become less effective in low-density electrode configurations where the average signal does not adequately represent overall brain activity. These techniques reduce reference bias and improve topographical representation, making them especially advantageous for frequency-domain and functional connectivity analyses, both commonly used in EEG-based biometrics.

However, referencing is not merely a routine preprocessing task but a strategic methodological choice informed by factors such as stimulus characteristics, electrode configuration, and the desired biometric feature domain. In applications like EEG biometrics, a consistent and well-chosen referencing enhances signal reproducibility, inter-session stability, and classification accuracy by preserving subtle inter-subject neural variations essential for reliable identity recognition [172].

### 7.4. Segmentation

Segmentation [91,99] plays a pivotal role in EEG-based biometric systems, facilitating temporal organization and improving the interpretability of neural signals across diverse stimuli and modeling frameworks. In cognitive task paradigms, such as mental arithmetic, motor imagery, or keystroke dynamics, segmentation is typically aligned with task onset to isolate event-specific neural activity associated with cognitive effort or motor intent [42]. For event-related paradigms, including Auditory and Visual Evoked Potentials (VEPs), EEG signals are divided into epochs time-locked to stimulus presentation, enabling precise extraction and averaging of evoked potentials [17].

Even in the absence of external stimuli, as in resting-state EEG, segmentation remains indispensable. Continuous EEG recordings are divided using fixed or sliding windows (typically 1–4 s) to account for the non-stationary nature of brain activity and to enhance training data diversity across sessions [71]. Likewise, in emotionally evocative or multimedia-driven paradigms, segmentation aligns EEG activity with changes in audiovisual content to ensure proper temporal correspondence with elicited neural responses [173].

Beyond stimulus-specific tasks, segmentation serves several core functional roles in EEG biometrics. It enables the generation of multiple training samples from limited recording sessions, mitigates the impact of signal non-stationarity by focusing on localized temporal features, and ensures consistency in feature extraction across sessions and subjects. Furthermore, deep learning models such as Convolutional Neural Networks (CNNs) and Long Short-Term Memory (LSTM) networks inherently require fixed-size input segments [174,175], making segmentation not only a preprocessing necessity but also a structural prerequisite for efficient model training and evaluation.

However, segmentation also introduces important trade-offs in EEG biometric systems. Shorter windows improve sample generation and support low-latency authentication, but they may fail to capture stable subject-specific neural dynamics. Conversely, longer segments generally improve discriminability and classification accuracy at the cost of increased computational complexity and reduced responsiveness. Sliding-window approaches enhance temporal continuity and data diversity, particularly for deep learning models, although overlapping windows may introduce redundancy and optimistic performance estimates if subject-independent evaluation is not carefully maintained. These limitations indicate that segmentation strategies should be selected according to the target application, dataset characteristics, and real-time deployment constraints.

**Figure 14:**
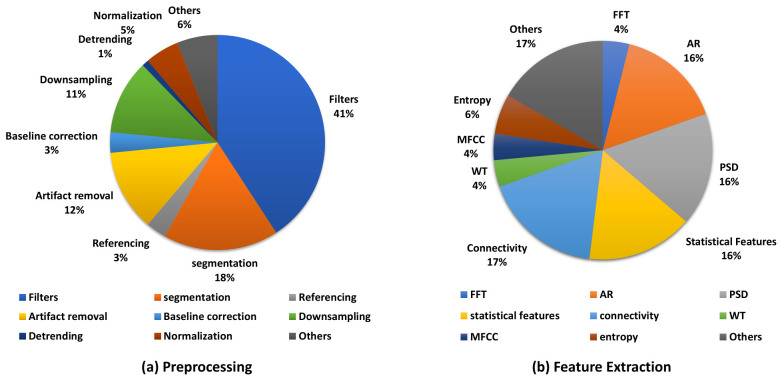
Overall percentage distribution of usage of methods in EEG-based biometric studies: (**a**) Preprocessing methods, and (**b**) Feature extraction methods.

The distribution of preprocessing techniques employed in EEG biometric studies is presented in Table 7 and illustrated in Figure 14a. As shown, filtering constitutes the most frequently applied method, underscoring its central role in eliminating noise and irrelevant frequency components. Segmentation is the next most common, primarily used to standardize trials and extract relevant epochs. Artifact removal techniques including approaches such as ICA, are also widely used to address ocular, muscular, and environmental interferences. Downsampling is often applied to reduce data dimensionality and computational load, while rereferencing and baseline correction serve to enhance signal consistency across electrodes. Less frequently, detrending and normalization are utilized to stabilize signals and prepare them for subsequent feature extraction. Overall, the analysis highlights a heavy reliance on traditional filtering, complemented by segmentation and artifact removal, reflecting a balance between noise suppression and data standardization in EEG biometrics preprocessing.

### 7.5. Preprocessing Tools

Several dedicated toolboxes have been developed to facilitate the preprocessing and analysis of EEG signals. Some of the widely used tools include PREP, EEGLAB, eConnectome, and FieldTrip.

The *Preprocessing Pipeline (PREP)* is an early-stage EEG signal processing pipeline that aims to identify bad channels and computation of robust average reference. The *PREP* pipeline function is based on the Matlab Signal Processing toolbox and the EEGLAB, freely available open-source Matlab toolbox for EEG analysis [176].

*EEGLAB* is an interactive MATLAB toolbox widely used for EEG signal processing. It supports a broad range of functionalities, including processing continuous and event-related brain signals, Independent Component Analysis (ICA), time-frequency analysis, artifacts rejection, channel selection, data visualization, event-related statistics [54,147], etc.

*Electrophysiological Connectome (eConnectome)* is an open-source MATLAB software package designed for the analysis of EEG, ECoG, and MEG data. It offers an interactive graphical interface and features for source estimation, imaging brain functional connectivity, and high-quality visualization [17,177].

*FieldTrip* is another advanced MATLAB software toolbox developed for the analysis of EEG and MEG signals. FieldTrip provides advanced analytical methods, including time–frequency analysis, source reconstruction using dipoles, and distributed source non-parametric statistical testing for robust group-level inference [119].

## 8. Feature Extraction

Feature extraction is the most important step in the EEG signal analysis, as it transforms EEG signals into discriminative attributes in the biometric user authentication pipeline. Given the inherently complex, non-stationary, and noise-prone nature of EEG signals, robust feature extraction is essential to improve the accuracy and reliability of classifier algorithms in biometrics [178]. This stage of biometric authentication involves selecting relevant characteristics from the EEG signals that serve as inputs to classifier models for identity recognition.

**Figure 15:**
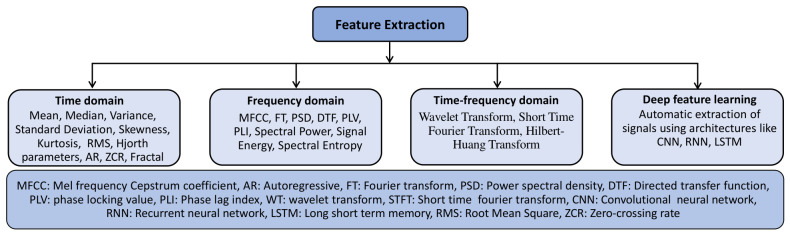
A pictorial illustration of different types of feature extraction techniques giving the readers an overview.

In this section, we present some feature extraction techniques that have been employed in the earlier works of EEG biometrics [4,54,98,99,149,162]. In this survey paper, we categorize the feature extraction techniques [179] into four main groups: Time domain, frequency domain, time-frequency domain, and deep feature learning, which explores the effectiveness in EEG-based biometrics by offering distinct advantages in capturing the underlying patterns and discriminative characteristics of EEG signals. Once the discriminative features are extracted from the preprocessed signals, they are supplied to classifier algorithms to perform subject authentication. The classifier learns decision boundaries that distinguish between individuals based on their unique neural signatures. Figure 14b shows the overall percentage distribution of different feature extraction methods in earlier work commonly used in our EEG biometrics literature. Figure 15 illustrates the types of feature extraction techniques under four main categories.

### 8.1. Time-Domain Features

The EEG signals are time series signals represented as a function of amplitude and time that vary according to the mind state of a person. The time-domain features can be easily extracted from the EEG signals with lower computational complexity compared to the frequency and time-frequency domain feature extraction techniques. These time-domain features can be extracted from raw or preprocessed [180] EEG data. Time-domain feature extraction methods in EEG-based biometric systems focus on analyzing time-varying EEG signals, capturing their amplitude distribution and temporal variability [176]. In the following, some of the widely used time-domain feature descriptors are briefly introduced.

*Statistical Descriptor* methods are among the most fundamental feature extraction approaches used in EEG-based biometric systems, that extract numerical information from time series data that describes the EEG signal’s behavior, distribution, variability, and shape to leverage the distributional and variability characteristics of EEG signals. The most commonly used statistical descriptors are mean, median, variance, standard deviation, skewness, kurtosis, and Root Mean Square (RMS) [4]. Similarly, the higher-order statistical features based on *Hjorth parameters*, such as Hjorth Activity (HA), Hjorth Mobility (HM), and Hjorth Complexity (HC), are also widely adopted techniques in the analysis of EEG signals [17,38,42]. Other statistical descriptors include entropy-based measures that have been used to differentiate states of brain activity over segmented EEG signals and have shown to be effective features for biometric applications [181]. Table 8 summarizes the definitions and mathematical formulations of these features.

*Autoregressive (AR)* modeling [46,49,110,182] is another prominent time-domain method that represents EEG segments as linear combinations of past samples, using the resulting coefficients as biometric features. AR modeling effectively captures temporal dependencies within the EEG signal and has been applied to both resting-state recordings [138] and event-related paradigms, to extract discriminative spectral features useful for biometric recognition [20,22].

*Zero-crossing rate (ZCR)* is a simple time-domain feature that measures how often a signal crosses the zero-amplitude axis within a given interval. From the resulting half-wave segments, features such as wave count, duration, peak amplitude, and instantaneous frequency (IF) can be extracted. In motor paradigms, these measures are useful because motor activity modulates sensorimotor rhythms, leading to characteristic oscillatory changes that ZCR-based features can effectively capture [140,183].

*Fractal and complexity* is the most powerful technique to measure hidden characteristics from EEG signals, quantifying the nonlinear and self-similar properties of brain signals. Unlike conventional statistical features, these measures characterize the intrinsic dynamical structure of EEG by estimating signal irregularity, scaling behavior, and long-range correlations [184]. As neural activity exhibits temporal variability, such features can effectively capture complexity patterns useful in biometric systems [127,185].

Time-domain features are computationally efficient, interpretable, and effective in capturing transient EEG characteristics associated with neural or cognitive activity. However, they often assume signal stationarity, which is rarely satisfied in practical EEG recordings, limiting their performance in long or highly variable sessions [4]. They are also sensitive to noise, artifacts, and inter-session variability, and are therefore frequently complemented by frequency or time–frequency features to improve robustness. Despite these limitations, time-domain features remain valuable for analyzing localized brain responses in specific paradigms. In stimulus-driven settings, time-domain features are typically extracted from Event-Related Potentials (ERPs), whose amplitude and latency variations provide discriminative temporal patterns useful for biometric authentication and cognitive state analysis [46].

### 8.2. Frequency Domain Features

Frequency-domain feature extraction techniques analyze EEG signals by transforming them from the time domain into a frequency distribution [44]. These features provide insight into the distribution of power across different frequency bands such as delta (1–4 Hz), theta (4–8 Hz), alpha (8–13 Hz), beta (13–30 Hz), and gamma (30–40 Hz). They are widely used in EEG biometrics, as they capture stable rhythmic patterns reflecting individual brain dynamics and cognitive states [65]. Within this domain, several categories of features can be derived depending on how spectral information is represented and interpreted.

The *Fourier Transform (FT)* decomposes EEG signals into their constituent frequency components, yielding complex coefficients whose magnitudes represent amplitude and whose phases indicate temporal offsets. The Fast Fourier Transform (FFT) [48,49], a computationally efficient implementation of the Discrete Fourier Transform (DFT), is commonly employed in EEG biometrics to estimate spectral features across standard EEG frequency bands [44,186]. Fourier-based spectral analysis is particularly suited to stimuli eliciting rhythmic or steady-state responses, such as steady-state Visually Evoked Potentials (SSVEPs), Auditory Evoked Potentials (AEPs), or other frequency-tagged paradigms. These features have been extracted from both resting-state EEG [120,187] and stimulus-driven protocols, including visual, auditory, and motor imagery tasks, where they reveal characteristic spectral modulations relevant for distinguishing user cognitive signals.

The *Power Spectral Density (PSD)* [188] quantifies the distribution of signal power over frequency, typically expressed in watts per hertz (W/Hz). In EEG biometrics, PSD features are extracted across a range of stimulus paradigms, including resting-state conditions [96,188], visual stimulation [100,189], auditory [190], and olfactory cues [48], as well as cognitive tasks such as mental arithmetic [191], word recall, and motor imagery. The PSD estimation can be performed using non-parametric methods [96] such as Welch’s method [47] or FFT-based techniques [54], or using parametric approaches like AR [182] and ARMA modeling [82]. Averaging PSD values within canonical frequency bands (delta, theta, alpha, beta, gamma) yields specific oscillatory signatures of an individual’s brain signals [191].

*Spectral Power* and *Signal Energy* are fundamental frequency-domain features derived from the Power Spectral Density (PSD), typically computed using Parseval’s theorem to relate time-domain energy to its spectral representation. In addition to total spectral power, other PSD-based descriptors such as maximum power and peak frequency within specific bands (e.g., the alpha band) are commonly extracted to capture dominant oscillatory characteristics in EEG biometric research [29].

*Spectral Entropy* [48,163], which was generated by applying the Shannon entropy concept to the power distribution of the Fourier transformed electroencephalograph (EEG), measures the irregularity or complexity of the power spectrum. Higher entropy values indicate more uniform (random) power distributions, while lower entropy reflects dominant, predictable rhythms. Spectral entropy features have been well utilized in biometric EEG research to effectively capture user spectral complexity [20,96,188]. This feature extraction is widely used in task-based paradigms (e.g., motor imagery or cognitive workload tasks), in which the entropy values change according to engagement levels [192,193].

*Connectivity features* capture the functional relationships and information flow between different brain regions [194]. Unlike single-channel spectral features, connectivity-based measures analyze inter-channel dependencies, reflecting the unique user neural communication patterns, making them less sensitive to EEG amplitudes. Connectivity can be quantified through statistical associations, phase relationships, or causal interactions, each providing complementary insights into coordinated neural activity. Frequency-domain approaches such as the Directed Transfer Function (DTF) [17] and Granger causality estimate directional information flow between channels, while phase-based measures such as the Phase Locking Value (PLV) [41] and Phase Lag Index (PLI) evaluate inter-channel synchronization by assessing the consistency of phase relationships [17,195].

*Mel Frequency Spectral Coefficients (MFCCs)* [46,110], although less commonly used, were originally developed for speech recognition, but now are explored for EEG-based biometric authentication. MFCCs are computed by applying a Fourier transform to the EEG signal, followed by a mel-scale filter bank that models human auditory perception [151]. These features are particularly effective when extracted from EEG signals recorded during auditory or speech-related stimuli [110]. Additionally, they may not fully capture nonlinear or non-stationary EEG properties, so they are often combined with other features such as entropy, fractal dimension, or PSD-based measures [5].

### 8.3. Time-Frequency Domain Features

Time-frequency domain features are widely used in EEG biometrics as they provide a joint representation of brain activity in both temporal and spectral dimensions. Unlike purely time or frequency domain measures, these features capture how oscillatory activity evolves over time, making them especially suitable for analyzing the dynamic and non-stationary nature of EEG signals.

The commonly used time-frequency domain techniques are *Wavelet Transform (WT)* and *Short-Time Fourier Transform (STFT)* [45,196]. WT, particularly based on the Discrete Wavelet Transform (DWT), is a transform that decomposes a signal into a number of sets (frequency bands), where each set is a time series of coefficients describing the time evolution of the signal in the corresponding frequency band and assists in the analysis of signals that have seen abrupt or dramatic shifts [45]. Extensions of WT such as Wavelet Packet Decomposition (WPD) and entropy-based measures (e.g., wavelet entropy), have also been used to quantify energy distribution and irregularity across sub-bands [197]. Similarly, STFT applies a sliding window to analyze how frequency components evolve over time, thereby dividing the signal into short overlapping windows, and the Fourier transform is applied to each segment [2].

Additionally, there are adaptive methods like the Hilbert–Huang Transform (HHT) [198], which is another method apart from WT and STFT used in EEG biometrics. HHT decomposes EEG signals into Intrinsic Mode Functions (IMFs) using Empirical Mode Decomposition (EMD), offering an adaptive time-frequency analysis that can capture nonlinear and non-stationary signal characteristics.

Time-frequency features have been applied in EEG biometrics across diverse stimulus conditions, such as resting-state, event-related paradigms, and motor imagery tasks [199,200]. These features provide a rich set of discriminatory information and have been extensively studied for improving the accuracy and robustness of EEG-based authentication systems [2].

### 8.4. Deep Feature Learning

Traditional EEG-based biometric systems primarily rely on handcrafted feature extraction techniques, where informative temporal, spectral, statistical, or time-frequency features are extracted from EEG signals and subsequently classified using conventional machine learning algorithms such as Support Vector Machines (SVM) [48], k-Nearest Neighbors (KNN) [49], Linear Discriminant Analysis (LDA) [201], and Random Forests [155] but in recent studies, deep learning models [15] are increasingly used not only as end-to-end classifiers but also as feature extractors. In this approach, intermediate representations typically obtained from one of the fully connected or convolutional layers are treated as high-level discriminative features that capture complex spatio-temporal patterns in EEG signals. The extracted deep embeddings are then fed into conventional classifier models mentioned above for final decision-making [50,202].

A common strategy employed within deep feature learning is to use connectivity-based input measures, such as Phase Locking Value (PLV) and Coherence (COH) [47], which quantify inter-channel synchronization and correlation, that is represented as matrices or graphs and provided as input to deep models. These models then automatically extract abstract embeddings that encode individual specific brain connectivity patterns, capturing spatial and temporal relationships between regions in a stable and discriminative manner [175]. These deep features derived from PLV, COH, and other connectivity-informed inputs are applicable across a wide range of stimulus conditions [175], including resting-state, sensory-evoked paradigms (visual/auditory), motor imagery tasks [203,204], and Event-Related Potentials. By combining topological information from connectivity measures with hierarchical patterns learned by deep architectures, these features provide a rich and flexible representation that complements traditional spectral, time-frequency, and connectivity-based features in EEG biometric systems. To provide a clearer picture of research trends, Figure 14b illustrates the percentage distribution of commonly used feature extraction techniques found in our EEG biometrics literature.

To summarize, time-domain features are simple and effective in capturing subject-specific EEG characteristics, but they are sensitive to noise and exhibit poor stability across different time epochs, making them more suitable for resting-state EEG analysis [4]. The frequency-domain features provide better stability and discriminative capability by analyzing spectral EEG characteristics. Time-frequency methods further improve the analysis of non-stationary EEG signals by jointly capturing temporal and spectral information, making them well suited for motor-task EEG analysis [46]. The deep learning-based methods automatically learn discriminative spatial, temporal, and nonlinear EEG representations from raw or minimally processed signals. Although they often achieve superior performance, they require large datasets, high computational resources, and generally offer lower interpretability compared to conventional handcrafted feature extraction methods. The commonly used feature extraction methods are presented in Table 7.

## 9. Classification Methods

**Figure 16:**
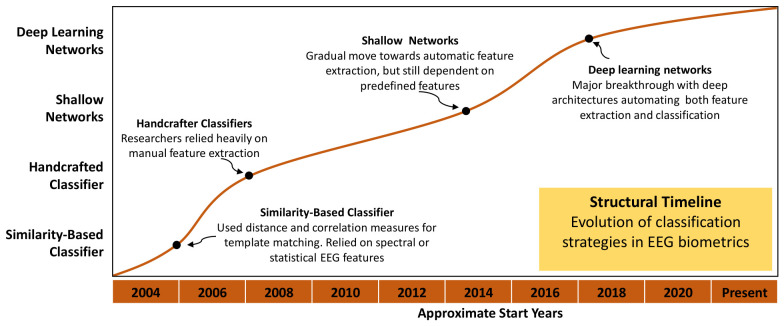
A pictorial representation that illustrates the beginning of handcrafted, shallow, and deep learning networks in EEG-based biometrics.

Once features are extracted, the classification step plays a vital role in interpreting these features to reliably identify individuals based on EEG signals. The extracted features are fed into classification algorithms that learn to recognize unique brain wave patterns corresponding to different subjects. Over the years, classification techniques in EEG biometrics have evolved significantly, beginning with conventional similarity-based approaches [205], which were later complemented by handcrafted feature-based methods [206]. More recently, the field has advanced toward shallow neural networks [197] and fully deep learning models [207,208,209], which can automatically learn hierarchical feature representations of EEG signals, leading to improved robustness and generalization performance in authentication tasks. These approaches vary widely in terms of complexity, dependency on manual feature engineering, and data requirements. The following subsections provide a detailed overview of these classification methods, highlighting their respective advantages and challenges within EEG-based biometric systems. Figure 16 shows the structured timeline reflecting when each category began gaining attenton in research.

### 9.1. Similarity-Based Classifier

One of the early and widely adopted approaches in EEG biometrics is the similarity-based classification framework, primarily valued for its conceptual simplicity and low computational complexity [210]. In this paradigm, a biometric template is first constructed during the enrollment phase, where representative EEG features are extracted from each subject under controlled recording conditions. These features may consist of time-domain statistical descriptors, frequency-band power features, or more discriminative representations such as spectral or connectivity-based measures, depending on the system design and application requirements.

During the authentication phase, an incoming probe EEG sample is processed through the same feature extraction pipeline and subsequently compared with the stored template. This comparison is typically performed using a range of similarity or distance metrics, including Euclidean distance, cosine similarity, correlation coefficients, Manhattan distance, or Dynamic Time Warping (DTW), depending on whether the feature representations are vector-based or sequential in nature.

Different similarity measures have been extensively investigated in the literature for EEG-based authentication. For example, *Euclidean distance* has been used to quantify dissimilarities between spectral features derived from brain signals [29,94], while *cross-correlation* has been applied to capture temporal similarity in resting-state EEG patterns [211]. The *cosine similarity* has been adopted to evaluate angular relationships between normalized feature vectors, particularly those derived from power spectral density representations [37]. The *Manhattan distance* has also been explored to measure absolute component-wise deviations in identity verification tasks [151]. In addition, *Dynamic Time Warping (DTW)* has been introduced to align nonlinearly shifted EEG sequences prior to similarity computation, thereby addressing temporal variations in brain activity [96]. These techniques have been applied either directly on raw EEG templates or on features extracted from time, frequency, or time-frequency domains. Although these approaches are computationally efficient and highly interpretable, they are generally more suitable for one-to-one authentication scenarios. A decision is then made by comparing the resulting similarity score against a predefined threshold, which is typically determined empirically using validation data to achieve an optimal trade-off between false acceptance and false rejection rates. If the similarity score satisfies the acceptance criterion, the claimed identity is verified; otherwise, the system rejects the input as an impostor attempt.

Despite their efficiency and ease of implementation, similarity-based methods are inherently sensitive to intra-subject variability in EEG signals, which may arise due to factors such as fatigue, cognitive state changes, electrode placement inconsistencies, or varying recording conditions. Consequently, these methods are often employed as baseline approaches or in scenarios where real-time performance and computational efficiency are prioritized over highly adaptive or learned representations.

### 9.2. Handcrafted Classifier

The handcrafted methods focus on manual feature engineering using domain expertise, ensuring interpretability but limiting adaptability. They work well with limited data and depend on feature selection quality. Some commonly used handcrafted methods are Linear Discriminant Analysis (LDA), K-Nearest Neighbor (K-NN), Support Vector Machine (SVM), Markov Model, and Random Forest (RF).

*Linear Discriminant Analysis (LDA)* [201] assumes that EEG features for each class follow a Gaussian distribution. It projects high-dimensional EEG feature vectors onto a lower-dimensional space where the distance between classes (i.e., different individuals) is maximized, making it useful for both classification [45] and dimensionality reduction. In the EEG biometrics literature, LDA [212] is often applied to features such as power spectral densities, band power ratios (alpha, beta, gamma, theta, delta), or Event-Related Potential (ERP) amplitudes and latencies. The augmentation of these features helps distinguish individuals based on the characteristic patterns of their brain activity in response to specific stimuli, such as P300 responses [213] from visual tasks or alpha/beta rhythms during motor activity.

*K-Nearest Neighbor (K-NN)* [48,49] is a non-parametric supervised learning algorithm frequently used in EEG biometrics for its simplicity, interpretability, and robustness with limited training data. The training set typically comprises EEG feature vectors extracted from spectral characteristics (e.g., band power in alpha, beta, gamma, theta, delta), time-domain features, or Event-Related Potentials (ERPs) and latencies [214]. During classification, an unlabeled EEG feature vector is assigned to the class that is most frequently represented among its k-nearest neighbors in the feature space which is determined using distance metrics such as the Euclidean distance. The parameter *k* critically influences performance, as it balances noise sensitivity and generalization. Despite its simplicity, K-NN serves as an effective baseline classifier in EEG biometrics, capable of distinguishing individuals based on the inherent similarity of their neural patterns.

*Support Vector Machine (SVM)* [48,155,201] is a supervised learning algorithm widely used in EEG biometrics for classification tasks. Its core principle is to find an optimal hyperplane in an *N*-dimensional feature space that maximizes the margin between classes. Using kernel functions, SVMs can effectively handle both linear and nonlinear separations in EEG data. As biometric authentication involves multiple subjects, SVMs are typically extended using one-vs-all or one-vs-one schemes for multi-class discrimination. For user authentication in EEG biometrics, Support Vector Machines are commonly used when the number of features is large compared to the number of subjects or training samples, as they effectively handle high-dimensional data, reduce the risk of overfitting, and provide robustness in capturing discriminative neural patterns. Commonly used feature vectors alongside SVM include spectral power [89], time-frequency features, and event-related potentials. Several studies have demonstrated high classification performance [17,89,197], and advanced methods incorporating brain connectivity and graph embeddings with SVMs have achieved near-perfect accuracies [98,99,202]. SVM remains a benchmark classifier in EEG biometrics due to its strong generalization on noisy neural data, capacity to model complex feature spaces, and consistent state-of-the-art performance across diverse experimental paradigms.

*Extreme Gradient Boosting (XGBoost)* [215] is an ensemble learning algorithm based on gradient-boosted decision trees, designed to maximize classification accuracy while maintaining computational efficiency. In EEG biometrics, XGBoost is typically employed after extracting handcrafted features such as Power Spectral Density (PSD), wavelet coefficients, entropy measures, or Event-Related Potentials (ERPs). Rather than learning features directly from raw EEG, it constructs a series of decision trees where each subsequent tree corrects the errors of the previous ones, progressively refining the model [216]. This makes XGBoost particularly effective for small-to-medium-sized EEG datasets, where deep learning methods often risk overfitting. It is also capable of modeling nonlinear feature relationships and provides feature-importance scores, offering interpretability by highlighting which EEG channels or features contribute most to classification. Due to its scalability, robustness, and competitive accuracy, XGBoost has become a strong alternative to traditional classifiers [217].

*Markov Model* [89,151] is a probabilistic framework well suited for modeling sequential dependencies in EEG signals, where the probability of the next state depends only on the current state. The Hidden Markov Model (HMM) extends this by assuming that brain states are latent (hidden) and can only be inferred from observed EEG features such as spectral band powers or Event-Related Potentials [89]. For user authentication in biometric systems, this is a valuable attribute because neural activity exhibits temporal dynamics and non-stationarity. By modeling transitions between cognitive or rhythmic states, HMMs can exploit the temporal evolution of EEG patterns to improve subject authentication accuracy. They rely on manually extracted EEG features and statistical state-transition modeling. However, their integration with neural networks (e.g., HMM–RNN hybrids [218]) has also been explored for more advanced spatio-temporal feature learning in EEG biometrics [89].

*Random Forest (RF)* [48,91,155,219] is another supervised ensemble learning algorithm that combines multiple decision trees, each trained on bootstrapped subsets of the data with random feature selection. In EEG biometrics, RF is applied primarily on handcrafted features [220] such as band power in delta, theta, alpha, beta, and gamma ranges, statistical descriptors from time-domain signals, or ERP features evoked by visual or motor imagery stimuli. These features capture distinctive neural signatures, while RF provides robust classification by aggregating the predictions of diverse trees through majority voting. Since EEG data are often noisy and non-stationary, the ensemble nature of RF reduces overfitting and improves generalization across sessions.

### 9.3. Shallow Networks

Shallow networks have been widely used in EEG-based biometric systems, particularly before the dominance of deep learning models [197]. Unlike deep architectures with multiple hidden layers, shallow neural networks typically consist of one hidden layer (or very few layers) and are trained using handcrafted features extracted from EEG signals [33,221]. Shallow networks have proven to be effectively applied in EEG biometrics, especially useful in scenarios where datasets are relatively small and computational resources are limited [175,222].

*Artificial Neural Networks (ANNs)* are the foundational class of neural models inspired by biological neurons [215,223]. For EEG biometrics, ANNs are applied to classify handcrafted features such as band power, entropy, and Event-Related Potentials (ERPs) [214]. Their ability to model nonlinear relationships with a shallow architecture makes them suitable for small datasets and limited computational resources [224]. ANNs have proven effective across visual, auditory, and motor stimuli, enabling authentication based on consistent cognitive or rhythmic patterns elicited by these tasks [225]. Additionally, *Multilayer Perceptrons (MLPs)* with shallow architecture have also been used as classifiers in cascaded frameworks, often in combination with convolutional or recurrent components to enhance classification accuracy [154,155].

### 9.4. Deep Learning Methods

Deep learning methods have become central to EEG biometrics because they can directly learn discriminative spatial–temporal representations from raw EEG signals, reducing dependence on handcrafted feature extraction [207,208,209]. Compared with traditional machine learning approaches, deep architectures are more effective at modeling the nonlinear, non-stationary, and subject-specific characteristics of EEG signals [32,42,51,160,174,226]. Recent research has therefore shifted toward architectures capable of jointly capturing temporal dynamics, inter-channel relationships, and cross-session variability.

*Convolutional Neural Networks (CNNs)* remain the dominant architecture in EEG biometrics due to their ability to hierarchically extract local and global spatial–temporal patterns [174,226]. Both lightweight shallow CNNs and deeper CNN architectures have been widely explored in EEG biometric systems. Lightweight CNNs are computationally efficient and suitable for wearable or real-time authentication systems, while deeper CNN models provide stronger representation learning capability for modeling complex EEG variability [33,110,134,175]. CNN-based approaches can operate directly on raw or minimally processed EEG signals, reducing dependence on handcrafted feature engineering [227,228]. Architectures such as One-Dimensional Spatio-Temporal Convolution (1D-STC) [229], Two-Dimensional Local Spatio-Temporal Convolution (2D-LSTC) [230], Two-Dimensional Spatial–Temporal Convolution (2D-SPTC) [231], and Three-Dimensional Spatial–Temporal Convolution (3D-SPTC) [209] have demonstrated that performance improves when both temporal evolution and electrode topology are jointly modeled [209,229,230,231,232].

*Recurrent Neural Networks (RNNs)* were introduced to explicitly model temporal dependencies in EEG sequences [231]. Standard RNNs maintain hidden states that evolve across time, enabling short-term temporal context learning. However, gradient instability limits their ability to retain long-range dependencies, reducing effectiveness for long EEG sequences. Consequently, pure RNN architectures are less common in recent EEG biometric systems, but they remain important as the conceptual basis for more advanced recurrent models and hybrid CNN–RNN frameworks [226].

*Long Short-Term Memory (LSTM)* networks address the limitations of conventional RNNs through gated memory mechanisms that regulate information flow across long temporal intervals [51]. In EEG biometrics, LSTMs are particularly effective for modeling evolving neural dynamics in paradigms such as motor imagery and Visual Evoked Potentials, where discriminative information is distributed over time [158,174]. Their ability to preserve temporal context makes them more robust to session variability and temporal fluctuations compared with shallow sequential models.

*Gated Recurrent Units (GRUs)* [32,51] simplify the LSTM design by merging the input and forget gates into a single update gate, reducing the parameter count while preserving the ability to capture both short-term and long-term dependencies [233]. This efficiency makes GRUs attractive for resting-state EEG and resource-constrained scenarios such as wearable biometric systems. Despite their lighter architecture, GRUs have shown comparable accuracy to LSTMs in authentication tasks, especially when modeling transient brain responses where memory requirements are shorter [234,235].

*Self-Organized Operational Neural Networks (Self-ONNs)* extend conventional CNNs by replacing fixed linear convolutional operators with adaptive nonlinear operators learned directly from data [42]. This design improves the representation of nonlinear EEG characteristics and enhances generalization across recording sessions and subjects. Compared with standard CNNs, Self-ONNs demonstrate improved robustness to noise and intra-subject variability while reducing dependence on extensive preprocessing and manual feature engineering [42,236]. Their adaptive operator learning mechanism makes them particularly suitable for highly heterogeneous EEG datasets.

*Graph Convolutional Neural Networks (GCNNs)* model EEG recordings as graph-structured data, where electrodes are represented as nodes and functional connectivity relationships define graph edges [160]. Unlike Euclidean convolutions used in CNNs, graph convolutions operate on irregular spatial structures and therefore better capture inter-electrode dependencies [50]. This formulation is especially relevant for EEG biometrics because subject-specific connectivity patterns contain discriminative information beyond local spectral features. Recent studies demonstrate that GCNNs outperform conventional CNNs in scenarios where spatial brain-network interactions play a dominant role [237,238,239].

As illustrated in Figure 16, classifier development in EEG biometrics has evolved from handcrafted machine learning approaches toward representation-driven deep learning frameworks. Earlier studies primarily relied on classifiers such as SVM, kNN, and LDA operating on manually engineered spectral or statistical features [48,155,201,240,241,242]. Although these methods were computationally efficient and effective on smaller datasets, their performance was strongly dependent on feature quality and domain expertise.

Between 2010 and 2016, the use of shallow neural architectures such as ANNs and MLPs were on the rise, and introduced limited representation learning capability but still lacked sufficient depth to model complex EEG variability [154,222,224,243]. Since 2016, deep learning approaches have dominated EEG biometric research due to advances in GPU computing, larger EEG datasets, and end-to-end optimization frameworks [32,42,51,160,174,226]. Current trends indicate a transition from feature-centric pipelines toward architectures that jointly learn temporal, spatial, and connectivity-aware representations directly from raw EEG. Hybrid and graph-based models further reflect a broader movement toward integrating multiple representational paradigms to improve cross-session robustness and subject generalization.

**Figure 17:**
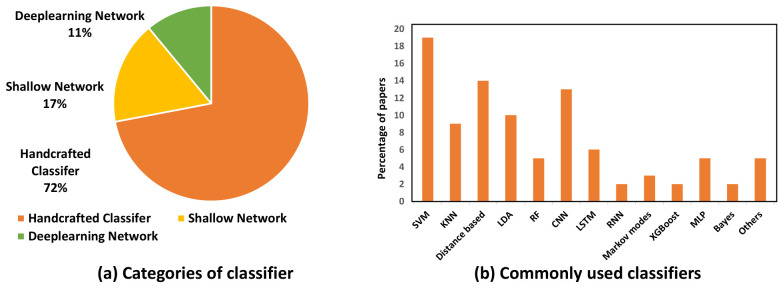
An illustration of the frequency of the classifiers used in our surveyed research papers: (**a**) Percentage usage of different categories of classifiers used in EEG-based biometric studies; (**b**) Percentage usage of some of the most commonly used classifiers.

Figure 17a illustrates the percentage of usage of individual classifiers in the EEG-based biometric studies. It shows the proportion of papers employing the handcrafted, shallow networks, and deep learning-based network classifier. Figure 17b, depicts the percentage of the most commonly used classifiers in the literature for EEG biometric applications. It can be observed that SVM dominates due to its robustness and simplicity, while other classifiers are less frequently used. These statistics provide a clear visual representation of the prevalence and distribution of classifier types.

Similarity-based classifiers are commonly employed in non-transformed domains where EEG features are represented as real-valued vectors. As observed from Table 7, early EEG biometric studies predominantly relied on handcrafted spectral and statistical features such as PSD, AR coefficients, FFT, MFCC, coherence measures, and functional connectivity descriptors, which were typically combined with conventional classifiers including SVM, HMM, LDA, LVQ, Random Forest, and distance-based matching methods. Among these, SVM appears as one of the most frequently adopted classifiers due to its effectiveness in handling high-dimensional EEG feature spaces and limited training samples. Similarity-based approaches such as Euclidean distance, cosine distance, Mahalanobis distance, template matching, and matching-score frameworks have also demonstrated competitive performance, particularly in verification-oriented EEG biometric systems.

The table further shows a gradual transition from handcrafted feature engineering toward neural-network-based approaches. Shallow neural networks, including MLP, ANN, and feed-forward neural networks, were introduced to model nonlinear relationships in EEG features; however, their reported performance and generalization capability were often dependent on the quality of manually extracted features. More recently, deep learning architectures such as CNNs, LSTMs, GRUs, autoencoders, and hybrid deep networks have become increasingly prevalent, particularly for resting-state, VEP, SSVEP, and motor imagery paradigms. These methods automatically learn hierarchical spatial–temporal representations directly from raw or minimally processed EEG signals and generally report the highest authentication performance in the surveyed literature.

Despite these advances, Table 7 also reveals that high reported accuracies are frequently obtained under controlled laboratory conditions and specific acquisition protocols. Handcrafted and shallow-learning approaches remain attractive due to their lower computational requirements, greater interpretability, and suitability for limited datasets, whereas deep learning models require substantially larger datasets, increased computational resources, and often exhibit reduced interpretability. Furthermore, the non-stationary nature of EEG signals, session-to-session variability, and changes in cognitive or emotional states continue to affect the long-term robustness of all classifier categories, highlighting the need for improved cross-session generalization and real-world validation.

## 10. Evaluation Protocols

Evaluating an EEG-based biometric system requires carefully designed experimental protocols that reflect how the system would operate in real-world conditions. Unlike conventional classification problems, biometric authentication must be robust to multiple sources of variability, such as time, task, and hardware differences [16]. A well-defined evaluation protocol ensures that reported performance is not overly optimistic and that comparisons across studies remain meaningful and reproducible. In general, evaluation protocols can be categorized according to the type of variability they introduce between the training and testing sets, as described below [139].

*Intra-stimulation or Intra-session* evaluation protocols train and test the model using EEG data collected within the same recording session, often under identical cognitive tasks or stimuli. This is the simplest and most optimistic evaluation setting, typically yielding the highest accuracy [93]. It primarily assesses the short-term repeatability of EEG biometric features, as electrode placement, mental state, and environmental conditions remain stable. However, strong performance under this condition does not guarantee real-world usability due to the inherent non-stationarity of EEG signals over time [37,244,245].

*Inter-stimulation (or Inter-task)* evaluation protocols train and test on EEG data collected from the same subjects but under different cognitive or sensory tasks. This evaluation examines whether identity-specific neural signatures remain stable across task variations. Systems that generalize well across tasks indicate that the extracted features capture person-specific characteristics rather than stimulus-driven patterns, making such protocols critical for developing task-independent and naturalistic biometric systems [245,246].

In the *cross-subject* evaluation protocol, models are trained on data from a subset of participants and tested on entirely unseen individuals. This evaluation assesses the generalization ability of the model and ensures that it does not overfit to users present in the training dataset [247]. Although cross-subject generalization is not directly required for biometric authentication, it is widely used in deep learning research to validate robustness and to identify potential dataset-specific biases [248].

*Cross-session* evaluation protocols are among the most stringent in EEG biometrics. Here, training and testing are performed on recordings from the same subjects but collected across different sessions, days, or time intervals. This protocol captures temporal variability arising from changes in electrode positioning, user fatigue, emotional state, and environmental factors. Cross-session performance is considered a key indicator of system reliability and long-term template stability [99,115].

*Cross-device* evaluation investigates whether a model trained on EEG data from one acquisition device can generalize to data recorded using different hardware. The rise of low-cost wearable EEG headsets makes this evaluation increasingly important [249]. Device differences in sensor type (for instance, wet *v*/*s* dry electrode devices), spatial montage, amplifier characteristics, impedance levels, and sampling rates can significantly alter signal properties. Systems that demonstrate robustness across devices are better suited for practical and portable biometric authentication deployments [250].

Among these protocols, intra-session evaluation provides only an optimistic upper-bound estimate of performance and does not reflect real-world deployment conditions. In contrast, cross-session evaluation should be considered the primary benchmark for EEG biometric systems, as it captures temporal non-stationarity and session variability. Furthermore, cross-day, cross-device, and template-aging evaluations are essential for assessing long-term stability and practical usability in real-world authentication scenarios. Future EEG biometric studies should prioritize these evaluation protocols to ensure meaningful and deployment-relevant performance reporting.

## 11. Performance Metrics

Performance metrics serve as an essential foundation for evaluating the effectiveness and reliability of EEG-based biometric systems. As these systems authenticate individuals using brain activity, a rigorous evaluation framework is necessary to properly assess their performance. Due to the inherently noisy, low Signal-to-Noise Ratio (SNR), and non-stationary characteristics of EEG signals, traditional biometric evaluation metrics must often be interpreted carefully or adapted to the application context [7]. This section presents the most widely used performance metrics in EEG biometrics, with a clear distinction between identification and verification scenarios.

EEG-based biometric systems are generally evaluated under two distinct paradigms: identification and verification. In the identification scenario (1:N matching), an EEG sample is matched against the templates of all the enrolled users and assigned to the identity. Its performance is commonly reported using classification accuracy or rank-based measures. In contrast, the verification scenario (1:1 matching) involves comparing a claimed identity against a stored template to decide whether the claim is genuine or an impostor. This is a threshold-based decision problem and is more relevant for real-world authentication systems, where security considerations are critical.

In classification-based evaluation, all performance measures are derived from the *Confusion Matrix*, which summarizes the outcomes of predictions in terms of True Positives (TP), True Negatives (TN), False Positives (FP), and False Negatives (FN). This matrix provides the basis for most biometric performance metrics [38].

Among identification-oriented metrics, *accuracy* measures the proportion of correctly classified samples out of all predictions [4,17,38,120,160], and is defined as:

(1) $$\mathrm{Accuracy}=\frac{TP+TN}{TP+TN+FP+FN}$$

While accuracy provides a general measure of performance, it may not fully reflect system reliability under class imbalance or varying data distributions. Therefore, it is often complemented by reporting the standard deviation of accuracy across cross-validation folds or repeated trials to assess stability and generalization capability [251]. A low standard deviation indicates consistent performance, whereas a high value suggests sensitivity to noise or overfitting [41].

However, accuracy alone is insufficient for biometric verification systems, as it does not account for decision thresholds or security–usability trade-offs. Therefore, verification-based evaluation relies on error-centric and threshold-dependent metrics.

The *False Acceptance Rate (FAR)* measures the likelihood that an impostor is incorrectly accepted as a genuine user [174,252]:

(2) $$\mathrm{FAR}=\frac{\mathrm{Number}\;\mathrm{of}\;\mathrm{False}\;\mathrm{Acceptances}}{\mathrm{Total}\;\mathrm{Number}\;\mathrm{of}\;\mathrm{Impostor}\;\mathrm{Attempts}}$$

The *False Rejection Rate (FRR)* measures the likelihood that a genuine user is incorrectly rejected [174,252]:

(3) $$\mathrm{FRR}=\frac{\mathrm{Number}\;\mathrm{of}\;\mathrm{False}\;\mathrm{Rejections}}{\mathrm{Total}\;\mathrm{Number}\;\mathrm{of}\;\mathrm{Genuine}\;\mathrm{Attempts}}$$

Since FAR and FRR depend on the decision threshold, biometric systems exhibit a trade-off between security and usability. In EEG-based verification systems, matching or similarity scores are computed between a test sample and a stored template. These scores form two distributions: the *genuine score distribution*, obtained from comparisons of samples belonging to the same individual, and the *impostor score distribution*, obtained from comparisons between different individuals. The degree of separation between these distributions reflects the discriminative capability of the biometric system. Greater separation generally leads to lower error rates, whereas significant overlap increases the likelihood of false acceptances and false rejections.

A decision threshold is then selected to determine whether a claimed identity should be accepted or rejected. If the matching score exceeds the threshold, the claim is accepted; otherwise, it is rejected. The choice of threshold directly influences the trade-off between FAR and FRR. A stringent threshold reduces the probability of accepting impostors but may increase the rejection of genuine users, whereas a relaxed threshold has the opposite effect. Consequently, performance is often analyzed over a range of thresholds using ROC and DET curves, while the operating threshold may be selected according to application-specific security requirements.

Most EEG biometric studies evaluate performance under a *closed-set* protocol, where every test sample belongs to an enrolled subject and the system assigns it to one of the known identities. In contrast, *open-set* evaluation allows previously unseen individuals to appear during testing. In such scenarios, the system must not only recognize enrolled users but also correctly reject unknown subjects. Open-set evaluation therefore provides a more realistic assessment of operational biometric systems and highlights the importance of robust threshold selection and impostor rejection capabilities.

The *Receiver Operating Characteristic (ROC)* curve illustrates the relationship between the False Acceptance Rate (FAR) and the True Positive Rate (TPR) as the decision threshold is varied. It provides a global view of the discriminative capability of the system. The Area Under the Curve (AUC) is often used as a summary metric, where higher values indicate better separation between genuine and impostor score distributions.

Complementary to ROC analysis, the *Detection Error Trade-off (DET)* curve plots the False Rejection Rate (FRR) against the False Acceptance Rate (FAR) on a normal deviate scale. This representation emphasizes performance in low-error operating regions, which are particularly important in high-security biometric applications. Unlike ROC curves, DET curves allow finer comparison of systems at very low error rates, making them suitable for strict authentication scenarios.

A widely used summary metric for verification performance is the *Equal Error Rate (EER)* [252], defined as the point where FAR equals FRR. It provides a single scalar value representing system performance and is commonly used for comparing biometric systems. The EER is typically obtained numerically by sweeping the decision threshold and identifying the point of intersection between FAR and FRR curves [186].

To provide an additional consolidated error measure, the *Half Total Error Rate (HTER)* [20] is defined as:

(4) $$\mathrm{HTER}=\frac{\mathrm{FAR}+\mathrm{FRR}}{2}$$

HTER provides a simple and interpretable summary of system performance, although it assumes equal importance of FAR and FRR.

In addition to verification-specific metrics, standard classification measures such as *True Positive Rate (TPR)*, *Specificity (True Negative Rate)*, *Precision*, and *F1-score* are also commonly reported. TPR, also known as Sensitivity or Recall, measures the proportion of genuine users correctly accepted, while Specificity measures the proportion of impostors correctly rejected. Precision evaluates the reliability of accepted identities, and F1-score provides a harmonic balance between Precision and Recall, especially in imbalanced datasets.

While identification-based metrics such as accuracy are useful for closed-set classification problems, EEG-based biometric authentication systems should primarily rely on verification-oriented metrics such as FAR, FRR, ROC, DET analysis, EER, and HTER to ensure meaningful evaluation of real-world security performance.

## 12. Challenges and Future Work

Despite significant progress in EEG-based biometric authentication, several critical challenges continue to limit its large-scale adoption and real-world deployment. A careful analysis of the literature reveals that most existing studies are conducted under controlled laboratory environments using relatively small and demographically homogeneous datasets, with limited attention given to long-term robustness, cross-session variability, subject adaptation, and practical deployment constraints. Although recent advances in deep learning have improved recognition accuracy, many systems still struggle to generalize across sessions, devices, cognitive states, and diverse user populations. In addition, issues related to explainability, privacy preservation, ethical governance, and computational efficiency remain insufficiently addressed. Consequently, future EEG biometric research must move beyond offline classification accuracy toward adaptive, interpretable, secure, and deployable authentication frameworks capable of operating reliably in realistic environments.

Among the various unresolved challenges, physiological and demographic variability remain key factors affecting the stability, permanence, and reliability of EEG biometric systems. Human brain activity is inherently influenced by age, neurological health, cognitive condition, fatigue, medication, emotional state, and demographic diversity, all of which can introduce significant variations in EEG signatures over time. Such variability complicates the development of generalized biometric templates and poses substantial challenges for long-term authentication in real-world deployment scenarios.

A synthesis of the existing literature indicates that the challenges in EEG-based biometric authentication are deeply interdependent and cannot be effectively addressed in isolation. Across the data, model, and deployment levels, a consistent pattern emerges: limited dataset diversity and demographic bias constrain representational robustness, while poor cross-session and cross-device generalization highlights the absence of stable and transferable biometric templates. Furthermore, the lack of effective subject-adaptive and lifelong learning mechanisms prevents sustained performance in realistic environments, particularly under changing physiological and contextual conditions. These coupled limitations indicate that the primary bottleneck is not individual algorithmic performance, but the absence of unified frameworks that jointly integrate data diversity, adaptive learning, and deployment-aware design. Therefore, future research should prioritize holistic, continuously adaptive biometric systems rather than incremental improvements within isolated methodological components.

The following subsections systematically present these challenges and emerging research directions in EEG-based biometric authentication.

### 12.1. Physiological and Demographic Variability

*Age* plays a significant role in the performance and reliability of EEG-based biometric authentication systems. Aging introduces several challenges due to physiological, cognitive, and neurofunctional changes that affect the stability of EEG signatures [253]. Structural and functional alterations in the aging brain, such as reduced neural synchrony, slower oscillatory rhythms, and modifications in cortical connectivity, can directly impact commonly used EEG biometric features [23]. Empirical studies show that normal aging disrupts spectral power distributions, alters oscillatory dynamics, and increases background neural noise, ultimately reducing the consistency of EEG-based biometric patterns over time [254]. Moreover, age-related variability increases in spatial and temporal EEG have been reported, including changes in alpha band correlations between brain regions, highlighting notable drift in neural signatures between young and older adults [255].

A major limitation in current EEG biometric research is that most studies rely on young, healthy participants, typically below the age of 30 years, despite evidence that EEG signal complexity evolves nonlinearly across the lifespan [23,255,256]. Older adults often exhibit greater intra-subject EEG variability due to fatigue, medication effects, and neurological conditions, reducing template permanence and increasing false rejection rates during long-term deployment. These findings highlight the need for age-invariant feature representations, adaptive learning frameworks, and periodic template recalibration strategies capable of maintaining stable authentication performance across different age groups [22,257].

*Neurological disorders* such as epilepsy, Parkinson’s disease, and Alzheimer’s disease can substantially alter neural activity patterns and disrupt the stability of biometric EEG features [258,259]. These conditions affect oscillatory rhythms, neural synchrony, and functional connectivity, introducing persistent deviations in EEG signatures used for authentication. Consequently, systems trained on healthy populations often perform poorly when applied to individuals with atypical neural dynamics. Developing clinically inclusive EEG biometric systems will therefore require disorder-aware models, adaptive feature extraction strategies, and personalized templates capable of compensating for pathological variability. Such developments are especially important because EEG biometrics may provide accessible authentication alternatives for individuals with physical or motor impairments [260].

Similarly, *mental health* conditions including depression, anxiety, and schizophrenia can influence EEG characteristics in more transient and state-dependent ways [261]. Emotional stress, fatigue, cognitive load, and mood fluctuations can alter frontal asymmetry, alpha-band activity, and event-related responses, thereby affecting authentication consistency. Since mental health conditions are highly prevalent worldwide, future EEG biometric systems must incorporate adaptive and mental-state-aware modeling approaches to ensure robustness and fairness across diverse psychological conditions. These effects may also vary across sex and gender-diverse populations, further emphasizing the importance of inclusive and demographically balanced datasets [262].

Expanding EEG biometric research across diverse *ethnic*, cultural, and demographic populations is equally essential for developing fair and generalizable systems. Variations in skull morphology, scalp thickness, hair density, sleep habits, stress exposure, diet, educational background, and cultural practices can all influence EEG acquisition quality and neural activity patterns [263,264,265]. Genetic ancestry has also been associated with differences in oscillatory dynamics and cortical connectivity [266,267]. However, many publicly available EEG datasets remain geographically and demographically limited, increasing the risk of algorithmic bias and reduced generalization [268]. Future studies should therefore prioritize large-scale, multi-ethnic, and longitudinal datasets to improve fairness, reduce demographic bias, and support globally deployable EEG biometric systems [269,270].

There is also limited consideration of how diverse *demographic* and *lifestyle* factors influence EEG signals used for biometric authentication. Variations across gender, educational background, cultural practices, dietary habits, sleep quality, and linguistic or communication disabilities can all introduce measurable differences in brain activity that may affect biometric stability and fairness [271]. For instance, gender-specific differences in cortical activation, hormonal cycles, or stress responsiveness can alter spectral power distributions. Educational and cognitive background may influence baseline neural efficiency and task-related EEG responses, potentially biasing systems that rely on cognitive stimulation paradigms. Linguistic diversity further contributes to differences in neural processing patterns, particularly in tasks involving semantic, auditory, or emotional stimuli. Lifestyle factors such as diet, caffeine consumption, fatigue, or irregular sleep, which are known to modulate EEG rhythms, may also degrade intra-subject consistency. Despite these well-established influences, most EEG biometric datasets remain limited to young, educated, and culturally homogeneous populations, which restricts generalization and may introduce demographic bias into real-world applications. Therefore, future EEG biometric research must incorporate diverse participant groups and systematically analyze demographic and lifestyle effects to develop equitable and population-wide authentication systems.

### 12.2. Practical Deployment and User Acceptance Challenges

Beyond physiological variability, several practical deployment and usability challenges must be addressed before EEG biometrics can become viable for everyday authentication applications.

Although EEG offers unique advantages as a biometric trait, it is highly variable and susceptible to noise. Integrating EEG with other physiological or behavioral modalities such as ECG, EMG, gait, or face recognition can improve authentication accuracy, robustness, and resistance to spoofing attacks. Such *multimodal systems* exploit complementary information from different modalities to reduce false acceptance and rejection rates under varying conditions. However, challenges related to sensor integration, synchronization, computational complexity, and privacy remain important research concerns [20].

Despite promising performance results, EEG biometric studies are still typically conducted on relatively small subject populations. Limited public acceptance and awareness of EEG technologies also restrict broader adoption. Many users perceive EEG devices as uncomfortable or associate them with medical procedures, while others express concerns regarding the privacy of neural data that may reveal cognitive or health-related information.

Most EEG biometric systems focus primarily on offline classification, whereas *real-time authentication* and *continuous authentication* remain comparatively underexplored. Real-time deployment introduces challenges including low-latency preprocessing, efficient feature extraction, lightweight classification, online artifact removal, and handling intra-session and inter-session variability [32,197]. Recent studies have demonstrated promising compact deep-learning models, such as EEGNet variants and CNN-LSTM architectures, for streaming EEG analysis and edge-device implementation [2]. However, benchmarking inconsistencies, limited privacy-preserving learning strategies, and insufficient security evaluation continue to hinder practical deployment [2,272].

Although EEG biometrics are generally more resistant to presentation attacks than traditional biometric modalities, they remain vulnerable [20] to replay attacks, template leakage, adversarial manipulation, and stimulus-dependent attacks. In addition, EEG variability across sessions and cognitive states further complicates secure real-world deployment. Therefore, future EEG biometric systems should incorporate robust threat modeling, secure template protection, and privacy-preserving authentication strategies for practical applications.

Thus, enabling practical EEG biometric deployment will require advances in adaptive modeling, efficient real-time processing, wearable hardware design, usability optimization, and secure authentication frameworks for reliable operation in real-world environments [273,274]. 

### 12.3. Ethical and Legal Challenges

In addition to technical and deployment limitations, EEG biometrics also introduce important ethical, legal, and societal concerns due to the highly sensitive nature of neural data.

EEG biometrics raise important *ethical* and *legal* challenges because brain signals contain deeply sensitive information that extends beyond personal identity, including cognitive states, emotional states, and potential neurological or psychological disorders [275,276]. This makes EEG data uniquely sensitive compared to other biometric identifiers. Issues related to privacy, data ownership, long-term storage, secondary use of data, and informed consent must be addressed rigorously to prevent misuse or unauthorized inference of mental or medical conditions [277]. In particular, participants must be clearly informed prior to data collection about the nature of EEG signals, the type of information that may be inferred, storage duration, data sharing policies, and possible future reuse, ensuring truly informed consent beyond a one-time agreement. There is also a risk of discrimination or bias if neural data are misinterpreted or mishandled. Therefore, robust regulatory frameworks, transparent data governance policies, and strict access control mechanisms are essential to safeguard users. Ethical guidelines must ensure that advancements in brain-based authentication respect individual autonomy, uphold societal values, and support the responsible development and deployment of EEG biometric technologies, in compliance with evolving long-term regulatory and neurorights frameworks [278].

### 12.4. Algorithmic and Modeling Challenges

Alongside physiological and ethical considerations, significant algorithmic and computational challenges continue to affect the reliability and scalability of EEG biometric systems.

Developing *robust algorithms* for EEG-based biometric authentication remains challenging due to issues related to feature extraction, classification, adaptability, and real-time deployment. A key difficulty lies in extracting stable and discriminative features from EEG signals, which are inherently non-stationary and vary across time, tasks, sessions, and physiological states [22,241]. Identifying features that remain invariant under such variability is non-trivial. High-dimensional EEG recordings also demand dimensionality reduction or representation learning methods, such as Principal Component Analysis (PCA) or deep autoencoders, yet reducing dimensionality without discarding relevant information remains an open challenge. Moreover, EEG signals are extremely sensitive to noise and artifacts arising from muscle activity, eye blinks, electrode impedance fluctuations, and environmental interference, requiring sophisticated preprocessing techniques. Methods such as Independent Component Analysis (ICA), adaptive filtering, and artifact subspace reconstruction are commonly used to suppress distortions while preserving biometric-relevant patterns [279,280].

Another major challenge is the high *inter-session* and *inter-subject* variability inherent in EEG signals. Brain wave patterns can shift significantly across recording sessions due to subtle differences in electrode placement, impedance changes, cognitive or emotional state fluctuations, and environmental conditions, all of which reduce the reproducibility of biometric features [127]. Traditional classifier models such as Support Vector Machines (SVMs) and k-Nearest Neighbors (k-NN) often struggle with the high dimensionality, nonlinearity, and low Signal-to-Noise Ratio of EEG data, leading to reduced generalization across sessions and subjects [22]. Deep learning models, including Convolutional Neural Networks (CNNs), Recurrent Neural Networks (RNNs), and transformer-based architectures, can better capture complex spatiotemporal EEG representations but require large and diverse datasets for training and are computationally demanding [33]. Ensuring a balance between algorithmic complexity and real-time performance is essential, especially for mobile, wearable, or embedded EEG authentication systems where computational resources, memory, and power consumption are constrained [281].

Most EEG biometric systems rely on black-box deep learning models that provide little insight into the decision-making process. Incorporating *explainable AI* techniques can enhance transparency, trust, and accountability by highlighting the features, frequency bands, or brain regions most relevant to authentication [282,283]. This is particularly important for regulatory compliance, debugging model errors, and ensuring fairness across diverse populations [284]. Future research should explore lightweight and interpretable models, or hybrid approaches that combine accuracy with explainability, to increase the acceptance of EEG biometrics in real-world applications [285].

The scarcity of large, diverse EEG biometric datasets remains a major obstacle to developing robust and generalizable machine learning models. Generative models, including Generative Adversarial Networks (GANs) and Variational Autoencoders (VAEs), offer promising solutions by synthesizing realistic EEG signals for data augmentation, balancing class distributions, and enhancing privacy [286,287]. *Synthetic EEG data* can significantly reduce reliance on time-consuming and resource-intensive data collection, while also supporting reproducible research and enabling benchmarking across studies [288]. Nevertheless, ensuring the authenticity, temporal dynamics, and inter-subject variability of generated EEG signals remains a key challenge. Poorly designed generative models may introduce artifacts or biases that compromise classifier robustness and degrade real-world performance [289]. Continued work is required to establish validation standards and quality metrics for synthetic EEG generation.

Further, the *lack of standardized EEG biometric datasets* and benchmarking frameworks remains a major limitation in the field, as most studies rely on small, institution-specific datasets recorded using different protocols, electrode montages, and hardware systems. This heterogeneity reduces reproducibility and makes it difficult to compare feature extraction methods, classification pipelines, and system performance across studies [290]. The absence of large, diverse, and publicly available EEG biometric datasets also restricts the development of deep learning models, which require substantial training data to generalize well across populations. Addressing these challenges requires the creation of standardized datasets, unified evaluation protocols, and benchmarking platforms. Future progress will depend on hybrid machine-learning approaches, adaptive biometric models, and lightweight real-time processing strategies supported by consistent datasets and evaluation standards [22,197].

### 12.5. Future Integration Directions

Looking ahead, future EEG biometric systems are expected to evolve beyond standalone authentication frameworks towards more integrated and adaptive neurotechnology ecosystems.

Future EEG biometric systems hold strong potential for integration with *Brain–Computer Interface (BCI)* applications, enabling seamless and continuous authentication in real-world scenarios such as assistive communication technologies, virtual and augmented reality environments, gaming systems, and neuroprosthetic control [291,292]. Embedding biometrics within BCIs can support personalized and secure user experiences by verifying identity during ongoing interaction rather than through discrete authentication events [293,294]. However, this integration introduces several challenges, including stringent low-latency requirements, ensuring user comfort during prolonged use, maintaining hardware interoperability across diverse BCI platforms, and defending against emerging security threats such as cognitive hacking or adversarial neural inputs [295]. Advancing EEG biometrics alongside BCIs opens promising avenues for adaptive, user-aware, and secure brain–computer interaction frameworks [296].

The reviewed literature suggests that the challenges in EEG-based biometric authentication exist across multiple interconnected priority levels. Immediate technical bottlenecks, particularly cross-session variability, feature instability, noise sensitivity, and limited dataset generalization, remain the most critical barriers affecting system reliability and robustness. Beyond these, medium-term deployment challenges such as real-time implementation, computational efficiency, wearable usability, and multimodal integration continue to limit practical large-scale adoption. In the longer term, ethical, legal, and societal concerns related to privacy preservation, informed consent, demographic fairness, and neural data governance will play an increasingly important role in shaping the responsible deployment of EEG biometric technologies. Addressing these challenges collectively will be essential for developing secure, adaptive, scalable, and trustworthy EEG-based authentication systems for real-world applications.

## 13. Conclusions

EEG biometrics has evolved from conventional handcrafted feature-based systems towards data-driven deep learning frameworks capable of learning complex spatial–temporal neural representations directly from EEG signals. Advances in spectral analysis, connectivity modeling, and deep architectures such as CNNs, recurrent networks, and graph-based models have significantly improved biometric recognition performance across a wide range of experimental paradigms.

Despite these developments, several challenges continue to limit the practical deployment of EEG biometric systems. A major issue is the inherent variability of EEG signals across sessions, mental states, acquisition devices, and electrode placements, which affects long-term stability and cross-session generalization. Furthermore, many existing studies rely on relatively small datasets collected under controlled laboratory conditions, making reproducibility and fair comparison between methods difficult. The absence of standardized acquisition protocols, evaluation metrics, and benchmarking frameworks further complicates large-scale validation.

Another important challenge concerns the balance between recognition performance and practical usability. Although deep learning approaches achieve state-of-the-art accuracy, they often require large training datasets, high computational resources, and lengthy calibration procedures, limiting their suitability for wearable and real-time authentication systems. In addition, issues related to explainability, privacy preservation, and robustness against adversarial manipulation remain insufficiently explored in current EEG biometric research.

Future work should therefore focus on developing lightweight and generalizable models capable of robust cross-session and cross-device adaptation. Greater emphasis is also needed on large-scale public datasets, standardized evaluation methodologies, explainable artificial intelligence techniques, and practical wearable EEG acquisition systems suitable for continuous authentication. Addressing these challenges will be essential for translating EEG biometrics from controlled research environments into scalable, secure, and real-world authentication applications. 

## Figures and Tables

**Table 1:** Shows the coverage of earlier research content covered by different EEG-based biometric survey papers.

Description	Q. Gui et al.	A. Bidgoly et al.	S. Zhang et al.	T. Shams et al.	C. Fidas et al.	Our Article
	2019 [29]	2020 [15]	2021 [32]	2022 [35]	2023 [16]	
Survey coverage	2007–2017	2015–2020	2012–2021	2000–2022	1998–2022	1990–2026
History of EEG biometrics	×	×	×	×	×	√
Elaborate EEG devices	×	×	×	×	×	√
EEG electrodes description	×	×	×	×	×	√
Electrode standards	×	×	×	×	×	√
Extensive focus on EEG database	Public	Public	×	×	×	Public & proprietary
Detailed data acquisition protocols	×	√	√	×	√	√
Full coverage preprocessing techniques	√	×	√	×	×	√
Exhaustive feature extraction methods	√	√	√	×	×	√
Extensive classification approaches	×	√	√	√	×	√
Extensive study on evaluation matrix	×	×	×	×	×	√
Recent challenges	×	×	×	×	×	√
Future works and considerations	×	×	√	√	√	√

**Table 2:** Functions of different parts and subparts of the brain along with their EEG channels (10–20 system).

Brain Parts	Subpart	Functions	Channels
**Cerebrum**	Frontal lobe	Movement, behavior, problem solving, speech	Fp1, Fp2, F7, F3, Fz, F4, F8
	Temporal lobe	Hearing, emotions, memory, language	T3, T4, T5, T6, A1, A2
	Parietal lobe	Intelligence, language, sensation, reasoning	P3, Pz, P4
	Occipital lobe	Sight, visual reception	O1, O2
	Central region	Voluntary movement and coordination	C3, Cz, C4
**Cerebellum**	–	Balance and muscle control	Not commonly measured in EEG
**Brainstem**	Midbrain	Controls eye movement, motor control, visual and auditory processes	Not commonly measured in EEG
	Pons	Controls facial movements, chewing and balance, regulates breathing	Not commonly measured in EEG
	Medulla Oblongata	Controls heart rate, blood pressure, and breathing, regulates reflexes like coughing, swallowing, and sneezing	Not commonly measured in EEG

**Table 3:** Brain wave characteristics, associated mental states, active brain regions, and observation in different age groups.

Brain Wave	Frequency Range	Amplitude (µV)	Mind State	Active Brain Regions	Observed in
**Delta (*δ*)**	0.5–4 Hz	20–200	Deep sleep, unconscious state, and deep restorative processes	Neocortex	Infants, sleeping adults
**Theta (*θ*)**	4–8 Hz	20–100	Dreams, creativity, meditation, reduced consciousness	Temporal lobes	Children, drowsy adults
**Alpha (*α*)**	8–12 Hz	20–60	Relaxed mental and physical state, eyes-closed resting condition	Occipital lobe	Awake adults
**Beta (*β*)**	12–30 Hz	5–20	Alertness, active thinking, strong engagement	Frontal lobe	Older children, adults
**Gamma (*γ*)**	30–100 Hz	<10	Problem-solving, cognitive processing, heightened perception	Temporoparietal region	Adults

**Table 4:** A summary of different types of EEG electrodes available based on hydration, placement, attachment, material, and functionality. It also provides information on their types, lifespan, signal quality, impedance levels, and skin preparation.

Category	Electrode Types	Signal Quality	Impedance	Skin Preparation	Typical Lifespan
**Based on Hydration**	Wet Electrodes	Excellent	Low (∼5–10 kΩ)	Requires cleaning & gel	6 months–2 years
	Dry Electrodes	Moderate	Medium (∼30–100 kΩ)	Minimal (may need slight pressure)	2–5 years
	Semi-Dry Electrodes	Good	Low (∼10–30 kΩ)	Minimal (saline or water-based)	1–3 years
**Based on Material**	Silver/Silver Chloride (Ag/AgCl)	Excellent	Low (∼5–10 kΩ)	Requires mild abrasion for best signal	6 months–2 years
	Gold Electrodes	High	Low (∼10–20 kΩ)	Minimal cleaning required	5–10 years
	Platinum Electrodes	Best	Very Low (∼1–5 kΩ)	None (implanted)	10+ years
	Tin Electrodes	Moderate	High (∼20–50 kΩ)	Requires abrasion & gel	1–2 years
	Conductive Polymer Electrodes	Moderate	Medium (∼30–100 kΩ)	No abrasion needed	3–5 years
	Carbon-Based (Graphene, CNT)	Excellent	Very Low (∼1–10 kΩ)	No abrasion needed	5+ years
**Based on Functionality**	Passive Electrodes	High	Low (∼5–10 kΩ)	Requires gel & cleaning	1–3 years
	Active Electrodes	Best	Very High (∼20–100 kΩ)	Minimal	3–5 years
	Hybrid Electrodes	High	Low (∼5–10 kΩ)	Minimal	3–5 years

**Table 5:** Summary of EEG devices under two categories (research grade and consumer grade) that provides description of devices, channels, sampling rate, standard electrode system, advantages, and disadvantages.

Category	Device Name	Description	Advantages	Disadvantages
Research-Grade	BCI2000 Instrumentation [24,36,47]	64 channels, 160 Hz sampling rate, international 10–10 system	Flexible and customizable, compatible with many EEG amplifiers	Steep learning curve, requires advanced setup
	BrainAmp DC Amplifier (BrainProducts, Gilching, Germany) [17,46]	32 channels, 500 Hz sampling rate, international 10–10 system	High-resolution data acquisition, supports up to 256 channels, excellent noise filtering	Expensive, requires conductive gel
	Cognionics HD72 Dry EEG System [50]	46 channels, 250 Hz sampling rate, international 10–10 system	Dry electrodes (no gel needed), high-density system, portable	More susceptible to motion artifacts than wet electrodes
	Galileo BE Light Amplifier [37]	19 channels, 256 Hz sampling rate, 10–20 system	High signal resolution, supports real-time processing, ideal for research labs	Expensive, requires professional setup
	g.USBamp Amplifier (g.tec) [3,41,71]	2/16 channels, 250/256 Hz sampling rate, international 10–20 system	High signal resolution, real-time data processing	High cost, requires trained users
	Neuroscan NuAmps [38]	40 channels, 1000 Hz sampling rate, 10–20 international system	High-resolution EEG signals, supports multiple electrode types, good for biometrics	Requires gel-based electrodes, expensive
	Neuroscan SynAmps [38,54]	64 channels, 1000 Hz sampling rate, 10–20 international system	Supports high-density EEG recording, low-noise system, ideal for biometric authentication	Expensive, requires technical expertise
Consumer-Grade	Emotiv Epoc+ EEG Headset [5]	14 channels, 128 Hz sampling rate, 10–20 international system	High signal quality, affordable, wireless, easy to use	Lower signal quality, uncomfortable for long sessions
	Emotiv INSIGHT Headset [91]	5 channels, 256 Hz sampling rate, 10–20 international system	Moderate signal quality, wireless, fixed headbands	Lower accuracy and resolution due to fewer electrodes, fit issues
	MindWave Mobile (NeuroSky, San Jose, CA, USA) [4]	Single channel, 512 Hz sampling rate, 10–20 international system	Low cost, wireless, easy to use, good for basic EEG analysis	Low accuracy, not suitable for research applications
	MUSE 2 Headband [92]	4 channels, 256 Hz sampling rate, 10–20 international system	Low cost, Bluetooth 4.0 connectivity, simple setup, good for self-help applications	Prone to artifacts, not suitable for longer experimental research

**Table 6:** Summary of public and proprietary EEG databases in terms of number of subjects, channels, EEG device, data acquisition protocols, number of sessions, and sampling rate, available for EEG biometric research.

Database	Subjects	Channels	EEG Device	Data Acquisition Protocols	Sessions	Sampling Rate
**Public Databases**						
PhysioNet MMI	109	64	BCI2000 system	Motor movement & imagery	Single session	160 Hz
SCCN EEG Database	14	32	SynAmps from Neuroscan	Categorization (Go/No-Go)	Single session	1000 Hz
BCI Competition III (IIIa)	3	60	Not specified (recorded by Graz BCI group)	Motor imagery (left hand, right hand, foot, tongue)	Single session	250 Hz
BCI Competition III (V)	3	32	Biosemi EEG system	Mental imagery (left hand, right hand, word generation)	Four sessions	512 Hz
BCI Competition IV (2a)	9	22 EEG + 3 EOG	g.USBamp biosignal amplifier	Motor imagery (left hand, right hand, foot, tongue movements)	Two sessions	250 Hz
BCI Competition IV (2b)	9	22 EEG + 3 EOG	g.USBamp biosignal amplifier	Motor imagery (left hand, right hand)	Five sessions	250 Hz
UCI EEG Database	122	64	NeuroScan (SynAmps amplifier)	Viewing picture objects	Single session	256 Hz
DEAP Dataset	32	32	BioSemi ActiveTwo system	Emotion (music videos)	Single session	512 Hz
ZuCo Database	12–18	64	Brain Products actiCHamp	Reading & cognitive tasks	Single session	500 Hz
PEERS dataset	345	129, 128	Geodesic Sensor Net, HydroCel Geodesic Sensor Net, BioSemi headcap	Word memorization tasks	6007 session	500 Hz
**Proprietary Databases**						
SEED-IV	15	64	Neuroscan (SynAmps amplifier)	Emotion recognition (movie clips)	Three sessions	1000 Hz
M3CV	106	64	Brain Products actiCHamp	Resting state, Evoked Potential, P300, motor movement	Two sessions	250 Hz
BED	25	4	Muse 2 headband (InteraXon Inc., Toronto, ON, Canada)	Affective stimuli, resting state, VEPs, math tasks	Three sessions	256 Hz
BIOMEX-DB	51	14	Emotiv Epoc+ headset	Participants pronounced single digits in Spanish	Single session	128 Hz

**Table 7:** Summary of EEG-based biometric authentication methods.

Authors	Stimuli	Preprocessing	Feature Extraction	Classifier	Performance
**Based on Similarity-Based Classifier**					
R.L. Rocha et al. [47], 2014	REC and REO	Rereferencing, Downsampling, Segmentation	PSD, COH	Mahalanobis distance-based classifier	CRR: 97.5% (REC), 96.26% (REO)
M. Fraschini et al. [94], 2015	REC and REO	BPF, Functional connectivity, eegfilt	PLI, Eigenvector centrality	Euclidean distance	EER: 0.044
Das R. et al. [37], 2016	VEP	CAR, Signal averaging	Frequency subband	Cosine distance-based classifier	95% confidence level
D. Kim and K. Kim [36], 2019	Resting state, Motor imagery	Segmentation	Functional networks using PLI and Eigenvector centrality	Matching scores	Accuracy: 95%
B. Chen et al. [149], 2020	VEP	Statistical feature processing	AR, DWT, FFT	Mallows distance-based classifier	Accuracy: 96.18%, HTER: 2.223%, FAR: 0.446%, FRR: 4%
A.J. Bidgoly [2], 2022	Resting state	Min-max scaling, Segmentation	CNN	Distance-based similarity (Euclidean, cosine, Manhattan)	Accuracy: 98.04%, EER: 1.96%, Precision: 97.45%, Recall: 98.66%
H. Qu et al. [150], 2024	VEP	Notch filter, Downsampling, Chebyshev IIR filter	CCA, ASP	Template matching	CRR: 99.55%
**Based on Handcrafted Methods**					
M. Poulos et al. [26], 1999	REC	BPF	FFT	LVQ	Accuracy: 100%
S. Marcel and J. del R. Millán [20], 2007	Motor imagery	Surface Laplacian filter	PSD	Gaussian Mixture Model, MAP	FAR: 4.8%, FRR: 0.02%, HTER: 6.6%
K. Brigham and B.V.K.V. Kumar [30], 2010	VEP, Imagined speech	Eye-blink artifact removal, BPF, Downsampling	AR	SVM, KNN	Accuracy: 98.96% (VEP), 99.76% (Imagined speech)
B.K. Min et al. [17], 2017	SSVEP	Segmentation, ICA	DTF	SVM	Accuracy: 98.60%
E. Maiorana et al. [151], 2018	REC, REO, Speech imagery, Mathematical computation	BPF, CAR, Segmentation, Downsampling	AR, MFCC, Bump modeling	HMM	EER: 2%
S. Keshishzadeh et al. [120], 2018	Motor movement and imagery, Resting state	DC offset removal, Butterworth LPF, Segmentation	Fractional Fourier Transform	SVM	Accuracy: 98.86%, EER: 1.04%, Specificity: 98.82%
Y. Di et al. [54], 2019	REO, REC	EEGLAB toolbox, LPF, Downsampling	PSD	SVM, LDA	Accuracy: 99%
A. Rahman et al. [91], 2021	REO and typing	Baseline Correction, BPF, Segmentation, Resampling	Synthetic Minority Oversampling Technique (SMOTE), Adaptive Synthetic Sampling (ADASYN)	Random Forest	Accuracy: 93.64%
P. A. Gonzalez et al. [5], 2021	Resting State, VEP, Mathematical computation	PREP pipeline	MFCC, ARRC, SPEC	HMM	EER: 0.3296, AUC: 0.6930
H. Yap [99], 2021	Eye closed and visual stimulation	FIR, Automatic artifact removal, Segmentation	Cross-correlation	SVM	Accuracy: 99.05% ± 0.85 Precision: 99.58% ± 0.55 Sensitivity: 99.34% ± 0.85 Specificity: 96.83% ± 4.35 F1-score: 99.4% ± 0.48 (for VEP)
G. Huang et al. [46], 2022	Resting state, SSVEP, SSAEP, SSSEP, Motor movement, Cognitive oddball, AEP, VEP, SEP	BPF, Notch filter, Rereferencing, Bad channel interpolation	MFCC, AR, PSD	SVM	Accuracy: 0.531, Precision: 0.544, Sensitivity: 0.570, Specificity: 0.941, F1-Score: 0.557
M. Usman et al. [141], 2026	VEP	PREP pipeline	Spectral, MFCC	RF, SVM, Decision, XGBoost	Accuracy: 98%
**Based on Shallow Networks**					
Haukipuro et al. [152], 2018	Image/Motor	–	PSD, MFCC	MLP	Accuracy: 83%
Bhateja et al. [153], 2019	Blinking	ICA, CCA	Wavelet, ICA	ANN	Accuracy: 80%
K.A. Sidek [154], 2019	REC, REO	Butterworth filter	wavelets, PSD	MLPNN	Accuracy: 75.8% (for REO) and 71.5% (for REC)
W. Kong et al. [41], 2019	VEP, driving, Motor imagery	Butterworth band-pass filter	PLV, PCA, LDA	Neural network	Accuracy: 97%
A.K.E. Khalil [155], 2024	VEP	BPF, ICA, FIR	PSD	MLP FFNN	Accuracy: 97% (MLP FFNN)
M. Rodriguez [142], 2021	REC, REO	Segmentation	PSD	ANN	Accuracy: 92.8 (for ANN)
**Based on Deep Learning Models**					
Schons et al. [156], 2018	REC, REO	Baseline correction, BPF	–	CNN	Accuracy: 99%
M.-H. Tsai [157], 2020	REC	Segmentation	Frequency domain	CNN	Accuracy: 96.80%
T. Wilaiprasitporn et al. [51], 2020	VEP	Downsampling, BPF, ICA, Referencing, Segmentation	PSD	LSTM, CNN, GRU	CRR: 100%
F. Balci [158], 2023	Motor movement and imagery, Resting state	Spectral bands, Z-score normalization	Correlation coefficient, RF	LSTM-MLP	Accuracy: 99.96%, Precision: 99.94%, Recall: 99.94%
B. Das [159], 2024	Resting state	Segmentation	Convolutional autoencoder	CNN	Accuracy: 99.89%
P. Lakhan et al. [160], 2025	Motor imagery, SSVEP	Downsampling, Butterworth BPF, Segmentation	Euclidean distance, PLV, Pearson correlation, RHO Index	EEG-BBNet, CNN	CRR: 99.26%

Acronyms: ICA = Independent Component Analysis, BPF = Band-Pass Filter, LPF = Low-Pass Filter, FIR = Finite Impulse Response, CAR = Common Average Reference, PREP = Preprocessing Pipeline, IIR = Infinite Impulse Response, PSD = Power Spectral Density, AR = Auto-Regressive, COH = Coherence, PLI = Phase Lag Index, MFCC = Mel Frequency Cepstral Coefficients, HMM = Hidden Markov Model, CNN = Convolutional Neural Network, LSTM = Long Short-Term Memory, GRU = Gated Recurrent Unit, CAR = Common Average Reference, FIR = Finite Impulse Response, RF = Random Forest, LDA = Linear Discriminant Analysis, EER = Equal Error Rate, FRR = False Rejection Rate, FAR = False Acceptance Rate.

**Table 8:** This table shows a summary of different time-domain features available for use in EEG-based biometrics.

Time-Domain Feature	Definition	Mathematical Expression
Mean	It is the average value of all data points in the time-domain EEG signal	$\mu =\frac{1}{n}\sum_{i=1}^{n}x_{i}$
Median	It is the midpoint of all ordered data points	$\mathrm{Median}=\left\{\begin{array}{cc} x_{\frac{n+1}{2}}, & \mathrm{if}\;n\;\mathrm{is}\;\mathrm{odd}\\ \frac{x_{\frac{n}{2}}+x_{\frac{n}{2}+1}}{2}, & \mathrm{if}\;n\;\mathrm{is}\;\mathrm{even} \end{array}\right.$
Variance	Measures the dispersion of EEG signal values around the mean	$\sigma^{2}=\frac{1}{n}\sum_{i=1}^{n}{\left(x_{i}-\mu \right)}^{2}$
Standard Deviation	Measures how EEG data spread around the mean value	$\sigma =\sqrt{\frac{1}{n}\sum_{i=1}^{n}{\left(x_{i}-\mu \right)}^{2}}$
Skewness	Measures the degree of asymmetry in the EEG distribution	$\frac{\sum_{i=1}^{n}{\left(x_{i}-\bar{x}\right)}^{3}}{\left(n-1\right)\;sd^{3}}$
Kurtosis	Describes the peakedness or flatness of the EEG distribution	$\frac{\sum_{i=1}^{n}{\left(x_{i}-\bar{x}\right)}^{4}}{\left(n-1\right)\;sd^{4}}$
Energy	Total squared magnitude of EEG signal samples	$\sum_{i=1}^{n}{\left|x_{i}\right|}^{2}$
Root Mean Square (RMS)	Measures signal magnitude and estimates power content	$RMS=\sqrt{\frac{1}{n}\sum_{i=1}^{n}x_{i}^{2}}$
Peak Amplitude	Maximum positive or negative deviation from zero reference	$\mathrm{Peak}=\max|x_{i}|$
Zero Crossing Rate	Number of times the signal crosses the zero axis	$ZCR=\frac{Z_{num}}{N}$
Hjorth Activity (HA)	Variance of the signal amplitude	$HA=\sigma^{2}$
Hjorth Mobility (HM)	Ratio of standard deviation of signal slope to signal amplitude	$HM=\sqrt{\frac{\sigma_{1}^{2}}{\sigma^{2}}}$
Hjorth Complexity (HC)	Represents the change in frequency of the signal	$HC=\sqrt{\frac{\sigma_{2}^{2}}{\sigma_{1}^{2}}-\frac{\sigma_{1}^{2}}{\sigma^{2}}}$

## Data Availability

No new data were created or analyzed in this study.

## References

[1] Puengdang S., Tuarob S., Sattabongkot T., Sakboonyarat B. (2019). EEG-Based Person Authentication Method Using Deep Learning with Visual Stimulation. Proceedings of the 2019 11th International Conference on Knowledge and Smart Technology (KST).

[2] Bidgoly A.J., Bidgoly H., Arezoumandothers Z. (2022). Towards a universal and privacy-preserving EEG-based biometric. Sci. Rep..

[3] Zeng Y., Wu Q., Yang K., Tong L., Yan B., Shu J., Yao D. (2018). EEG-Based Identity Authentication Framework Using Face Rapid Serial Visual Presentation with Optimized Channels. Sensors.

[4] Zhang R., Yan B., Tong L., Shu J., Song X., Zeng Y. (2019). Identity Authentication Using Portable Electroencephalography Signals in Resting States. IEEE Access.

[5] Arnau-González P., Katsigiannis S., Arevalillo-Herráez M., Ramzan N. (2021). BED: A New Data Set for EEG-Based Biometrics. IEEE Internet Things J..

[6] Daswani N., Elbayadi M. (2021). The Yahoo Breaches of 2013 and 2014. Big Breaches: Cybersecurity Lessons for Everyone.

[7] Jain A.K., Flynn P., Ross A.A. (2007). Handbook of Biometrics.

[8] Kaur P., Krishan K., Sharma S., Kanchan T. (2018). ATM Card Cloning and Ethical Considerations. Sci. Eng. Ethics.

[9] Jain A., Ross A., Prabhakar S. (2004). An introduction to biometric recognition. IEEE Trans. Circuits Syst. Video Technol..

[10] Ramachandra R., Busch C. (2017). Presentation Attack Detection Methods for Face Recognition Systems: A Comprehensive Survey. ACM Comput. Surv..

[11] Karampidis K., Rousouliotis M., Linardos E., Kavallieratou E. (2021). A comprehensive survey of fingerprint presentation attack detection. J. Surveill. Secur. Saf..

[12] Sharma D., Selwal A. (2023). A survey on face presentation attack detection mechanisms: Hitherto and future perspectives. Multimed. Syst..

[13] Balabanova I., Sidorova K., Georgiev G. (2024). Voice Profile Authentication Using Machine Learning. Eng. Proc..

[14] Pooshideh M., Beheshti A., Qi Y., Farhood H., Simpson M., Gatland N., Soltany M. (2024). Presentation Attack Detection: A Systematic Literature Review. ACM Comput. Surv..

[15] Jalaly Bidgoly A., Jalaly Bidgoly H., Arezoumand Z. (2020). A survey on methods and challenges in EEG based authentication. Comput. Secur..

[16] Fidas C.A., Lyras D. (2023). A Review of EEG-Based User Authentication: Trends and Future Research Directions. IEEE Access.

[17] Min B.K., Suk H.I., Ahn M.H., Lee M.H., Lee S.W. (2017). Individual Identification Using Cognitive Electroencephalographic Neurodynamics. IEEE Trans. Inf. Forensics Secur..

[18] Fallahi M., Arias-Cabarcos P., Strufe T. (2026). Advancing Brainwave-Based Biometrics: A Large-Scale, Multi-Session Evaluation. IEEE Transactions on Biometrics, Behavior, and Identity Science.

[19] Kamrud A., Borghetti B., Schubert Kabban C. (2021). The Effects of Individual Differences, Non-Stationarity, and the Importance of Data Partitioning Decisions for Training and Testing of EEG Cross-Participant Models. Sensors.

[20] Marcel S., del R. Millán J. (2007). Person authentication using brainwaves (EEG) and maximum a posteriori model adaptation. IEEE Trans. Pattern Anal. Mach. Intell..

[21] Sadeghi K., Sohankar J., Banerjee A., Gupta S.K.S. (2017). A novel spoofing attack against electroencephalogram-based security systems. Proceedings of the 2017 IEEE SmartWorld, Ubiquitous Intelligence & Computing, Advanced & Trusted Computed, Scalable Computing & Communications, Cloud & Big Data Computing, Internet of People and Smart City Innovation (SmartWorld/SCALCOM/UIC/ATC/CBDCom/IOP/SCI).

[22] Campisi P., La Rocca D. (2014). Biometric recognition using brain electroencephalogram signals. IEEE Trans. Inf. Forensics Secur..

[23] Zappasodi F., Marzetti L., Olejarczyk E., Tecchio F., Pizzella V. (2015). Age-related changes in electroencephalographic signal complexity. PLoS ONE.

[24] Yang S., Hoque S., Deravi F. (2019). Improved Time-Frequency Features and Electrode Placement for EEG-Based Biometric Person Recognition. IEEE Access.

[25] Berger H. (1929). Über das Elektrenkephalogramm des Menschen. Arch. Für Psychiatr. Und Nervenkrankh..

[26] Poulos M., Rangoussi M., Alexandris N. (1999). Neural network based person identification using EEG features. Proceedings of the IEEE International Conference on Electronics, Circuits and Systems.

[27] Anokhin A.P., Steinlein O., Fischer C., Mao Y., Vogt P., Schalt E., Vogel F. (1992). A genetic study of the human low-voltage electroencephalogram. Hum. Genet..

[28] van Beijsterveldt C., Molenaar P., de Geus E., Boomsma D. (1996). Heritability of human brain functioning as assessed by electroencephalography. Am. J. Hum. Genet..

[29] Gui Q., Blondet M., Laszlo S., Jin Z. (2019). A Survey on Brain Biometrics. ACM Comput. Surv..

[30] Brigham K., Kumar B.V.K.V. (2010). Subject identification from electroencephalogram (EEG) signa ls during imagined speech. Proceedings of the 2010 Fourth IEEE International Conference on Biometrics: Theory Applications and Systems (BTAS).

[31] De Gennaro L., Ferrara M., Vecchio F., Curcio G., Bertini M. (2005). An electroencephalographic fingerprint of human sleep. NeuroImage.

[32] Zhang S., Sun L., Mao X., Hu C., Liu P. (2021). Review on EEG-based authentication technology. Comput. Intell. Neurosci..

[33] Albaiati A.E., Akbar M.F., Hssayeni M.D., Khalil A., Wahab M.N.A., Weli S.S., Raheem E.A. (2025). Deep Learning Approaches for EEG-Based Biometrics: A Systematic Review. IEEE Access.

[34] Hu Y., Sun L., Mao X., Zhang S. (2024). EEG Data Augmentation Method for Identity Recognition Based on Spatial–Temporal Generating Adversarial Network. Electronics.

[35] Shams T., Hossain M.S., Mahmud M., Tehjib M., Hossain Z., Pramanik I. (2022). EEG-based Biometric Authentication Using Machine Learning: A Comprehensive Survey. ECTI Trans. Electr. Eng. Electron. Commun..

[36] Kim D., Kim K. (2019). Resting State EEG-Based Biometric System Using Concatenation of Quadrantal Functional Networks. IEEE Access.

[37] Das R., Maiorana E., Campisi P. (2016). EEG Biometrics Using Visual Stimuli: A Longitudinal Study. IEEE Signal Process. Lett..

[38] Gao Z., Dang W., Liu M., Guo W., Ma K., Chen G. (2021). Classification of EEG Signals on VEP-Based BCI Systems with Broad Learning. IEEE Trans. Syst. Man Cybern. Syst..

[39] Ron-Angevin R., Medina-Juliá M.T., Fernández-Rodríguez Á., Velasco-Álvarez F., Andre J.M., Lespinet-Najib V., Garcia L. (2021). Performance analysis with different types of visual stimuli in a BCI-based speller under an RSVP paradigm. Front. Comput. Neurosci..

[40] Hasan S.M.S., Siddiquee M.R., Marquez J.S., Bai O. (2022). Enhancement of Movement Intention Detection Using EEG Signals Responsive to Emotional Music Stimulus. IEEE Trans. Affect. Comput..

[41] Kong W., Wang L., Xu S., Babiloni F., Chen H. (2019). EEG Fingerprints: Phase Synchronization of EEG Signals as Biomarker for Subject Identification. IEEE Access.

[42] Rahman A., Chowdhury M.E., Khandakar A., Tahir A.M., Ibtehaz N., Hossain M.S., Kiranyaz S., Malik J., Monawwar H., Kadir M.A. (2022). Robust biometric system using session invariant multimodal EEG and keystroke dynamics by the ensemble of self-ONNs. Comput. Biol. Med..

[43] Taherisadr M., Joneidi M., Rahnavard N. (2019). EEG Signal Dimensionality Reduction and Classification using Tensor Decomposition and Deep Convolutional Neural Networks. Proceedings of the 2019 IEEE 29th International Workshop on Machine Learning for Signal Processing (MLSP).

[44] Sanei S., Chambers J. (2007). EEG Signal Processing.

[45] Abbas Seha S.N., Hatzinakos D. (2019). A New Approach for EEG-Based Biometric Authentication Using Auditory Stimulation. Proceedings of the 2019 International Conference on Biometrics (ICB).

[46] Huang G., Hu Z., Chen W., Zhang S., Liang Z., Li L., Zhang L., Zhang Z. (2022). M3CV: A multi-subject, multi-session, and multi-task database for EEG-based biometrics challenge. NeuroImage.

[47] Rocca D.L., Campisi P., Vegso B., Cserti P., Kozmann G., Babiloni F., Fallani F.D.V. (2014). Human Brain Distinctiveness Based on EEG Spectral Coherence Connectivity. IEEE Trans. Biomed. Eng..

[48] Pandharipande M., Chakraborty R., Kopparapu S.K. (2023). Modeling of Olfactory Brainwaves for Odour Independent Biometric Identification. Proceedings of the 2023 31st European Signal Processing Conference (EUSIPCO).

[49] Aydemir O. (2020). Odor and Subject Identification Using Electroencephalography Reaction to Olfactory. Trait. Du Signal.

[50] Wang M., El-Fiqi H., Hu J., Abbass H.A. (2019). Convolutional Neural Networks Using Dynamic Functional Connectivity for EEG-Based Person Identification in Diverse Human States. IEEE Trans. Inf. Forensics Secur..

[51] Wilaiprasitporn T., Ditthapron A., Matchaparn K., Tongbuasirilai T., Banluesombatkul N., Chuangsuwanich E. (2020). Affective EEG-Based Person Identification Using the Deep Learning Approach. IEEE Trans. Cogn. Dev. Syst..

[52] Cao J., Zhao Y., Shan X., Wei H.L., Guo Y., Chen L., Erkoyuncu J.A., Sarrigiannis P.G. (2022). Brain functional and effective connectivity based on electroencephalography recordings: A review. Hum. Brain Mapp..

[53] Kumar J., Bhuvaneswari P.T.V. (2012). Analysis of Electroencephalography (EEG) Signals and Its Categorization—A Study. Procedia Eng..

[54] Di Y., An X., He F., Liu S., Ke Y., Ming D. (2019). Robustness Analysis of Identification Using Resting-State EEG Signals. IEEE Access.

[55] Maldonado K.A., Alsayouri K. (2025). Physiology, brain. StatPearls.

[56] Moini J., Piran P., Moini J., Piran P. (2020). Chapter 6—Cerebral cortex. Functional and Clinical Neuroanatomy.

[57] Kandel E.R., Schwartz J.H., Jessell T.M. (2021). Principles of Neural Science.

[58] Blumenfeld H. (2010). Neuroanatomy Through Clinical Cases.

[59] Nolte J. (2008). The Human Brain: An Introduction to Its Functional Anatomy.

[60] Purves D., Augustine G.J., Fitzpatrick D., Hall W.C., LaMantia A.S., White L.E., Mooney R., Platt M. (2018). Neuroscience.

[61] Buzsáki G., Logothetis N., Singer W. (2013). Scaling Brain Size, Keeping Timing: Evolutionary Preservation of Brain Rhythms. Neuron.

[62] Katmah R., Al-Shargie F., Tariq U., Babiloni F., Al-Mughairbi F., Al-Nashash H. (2021). A review on mental stress assessment methods using EEG signals. Sensors.

[63] Abhang P., Gawali B.W., Mehrotra S.C. (2016). Technological Basics of EEG Recording and Operation of Apparatus.

[64] Seeck M., Koessler L., Bast T., Leijten F., Michel C.M., Baumgartner C., Heuser K., Beniczky S. (2017). The standardized EEG electrode array of the IFCN. Clin. Neurophysiol..

[65] Teplan M. (2002). Fundamental of EEG Measurement. Meas. Sci. Rev..

[66] Chi Y.M., Jung T.P., Cauwenberghs G. (2010). Dry-Contact and Noncontact Biopotential Electrodes: Methodological Review. IEEE Rev. Biomed. Eng..

[67] Wang F., Chen D., Fu R., Zhang X. (2024). Study on a Novel Flow-Controllable Semidry Electrode for EEG Signal Acquisition. IEEE Sens. J..

[68] Afif N., Pratama S., Haryanto F., Khotimah S., Suprijadi S. (2020). Comparison of Wet and Dry EEG Electrodes Based On Brain Signals Characterization in Temporal and Anterior Frontal Areas Using Audio Stimulation. J. Phys. Conf. Ser..

[69] Xiong F., Fan M., Feng Y., Li Y., Yang C., Zheng J., Wang C., Zhou J. (2025). Advancements in dry and semi-dry EEG electrodes: Design, interface characteristics, and performance evaluation. AIP Adv..

[70] Metting van Rijn A.C., Peper A., Grimbergen C.A. (1990). High-quality recording of bioelectric events. Med. Biol. Eng. Comput..

[71] Nakamura T., Goverdovsky V., Mandic D.P. (2018). In-Ear EEG Biometrics for Feasible and Readily Collectable Real-World Person Authentication. IEEE Trans. Inf. Forensics Secur..

[72] Harati A., Jahanshahi A. (2021). A reliable stretchable dry electrode for monitoring of EEG signals. Sens. Actuators A Phys..

[73] Lopez-Gordo M.A., Sanchez-Morillo D., Pelayo F. (2014). Dry EEG electrodes. Sensors.

[74] Moridera T., Rashed E.A., Mizutani S., Hirata A. (2021). High-Resolution EEG Source Localization in Segmentation-Free Head Models Based on Finite-Difference Method and Matching Pursuit Algorithm. Front. Neurosci..

[75] Wolpaw J., Birbaumer N., Heetderks W., McFarland D., Peckham P., Schalk G., Donchin E., Quatrano L., Robinson C., Vaughan T. (2000). Brain-computer interface technology: A review of the first international meeting. IEEE Trans. Rehabil. Eng..

[76] Wang F., Li G., Chen J., Duan Y., Zhang D. (2016). Novel semi-dry electrodes for brain–computer interface applications. J. Neural Eng..

[77] Bayat M., Safaie J., Beidokhti S.M., Wallois F. (2026). A comprehensive review of EEG electrode technologies: Advancements, applications, and future directions. Sens. Actuators A Phys..

[78] Stauffer F., Thielen M., Sauter C., Chardonnens S., Bachmann S., Tybrandt K., Peters C., Hierold C., Vörös J. (2018). Skin Conformal Polymer Electrodes for Clinical ECG and EEG Recordings. Adv. Healthc. Mater..

[79] Caton R. (1970). The Electric Currents of the Brain. Am. J. EEG Technol..

[80] Klem G. (1999). The ten-twenty electrode system of the International Federation. The International Federation of Clinical Neurophysiology. Electroencephalogr. Clin. Neurophysiol. Suppl..

[81] de Melo G.C., Forner-Cordero A., Castellano G. (2025). The role of the reference electrode in EEG recordings: Looking from an inverted perspective. Biomed. Phys. Eng. Express.

[82] Poulos M., Rangoussi M., Alexandris N., Evangelou A. (1999). Person identification based on parametric processing of the EEG. Proceedings of the IEEE International Conference on Electronics, Circuits and Systems (ICECS).

[83] Jurcak V., Tsuzuki D., Dan I. (2007). 10/20, 10/10, and 10/5 systems revisited: Their validity as relative head-surface-based positioning systems. NeuroImage.

[84] Fabregat-Sanjuan A., Pàmies-Vilà R., Rigo-Vidal A., Pascual Rubio V. (2023). Comparison of electrode position marking procedures on the cranial surface. Brain Behav..

[85] Michel C.M., Murray M.M. (2012). Towards the utilization of EEG as a brain imaging tool. NeuroImage.

[86] Oostenveld R., Praamstra P. (2001). The five percent electrode system for high-resolution EEG and ERP measurements. Clin. Neurophysiol..

[87] Shrara H., Ammar H., Nasseredine M., Charara J., Sbeity F. (2023). An EEG-Based Emotion Recognition Study Using Machine Learning and Deep Learning. Proceedings of the 2023 Seventh International Conference on Advances in Biomedical Engineering (ICABME).

[88] Schmidt A. (2016). Biosignals in human-computer interaction. Interactions.

[89] Kumar P., Singhal A., Saini R., Roy P.P., Dogra D.P. (2018). A pervasive electroencephalography-based person authentication system for cloud environment. Displays.

[90] La Rocca D., Campisi P., Scarano G. (2012). EEG biometrics for individual recognition in resting state with closed eyes. Proceedings of the 2012 BIOSIG—Proceedings of the International Conference of Biometrics Special Interest Group (BIOSIG).

[91] Rahman A., Chowdhury M.E.H., Khandakar A., Kiranyaz S., Zaman K.S., Reaz M.B.I., Islam M.T., Ezeddin M., Kadir M.A. (2021). Multimodal EEG and Keystroke Dynamics Based Biometric System Using Machine Learning Algorithms. IEEE Access.

[92] Mansi S.A., Pigliautile I., Porcaro C., Pisello A.L., Arnesano M. (2021). Application of wearable EEG sensors for indoor thermal comfort measurements. Acta IMEKO.

[93] Ma L., Minett J.W., Blu T., Wang W.S.Y. (2015). Resting State EEG-based biometrics for individual identification using convolutional neural networks. Proceedings of the 2015 37th Annual International Conference of the IEEE Engineering in Medicine and Biology Society (EMBC).

[94] Fraschini M., Hillebrand A., Demuru M., Didaci L., Marcialis G. (2015). An EEG-Based Biometric System Using Eigenvector Centrality in Resting State Brain Networks. IEEE Signal Process. Lett..

[95] Thomas K.P., Vinod A.P. (2018). EEG-Based Biometric Authentication Using Gamma Band Power During Rest State. Circuits Syst. Signal Process..

[96] Maiorana E., La Rocca D., Campisi P. (2016). On the Permanence of EEG Signals for Biometric Recognition. IEEE Trans. Inf. Forensics Secur..

[97] Zhang X., Yang C., Li F., Li Y., Fu B., Wang S., Zhang L., Wang H., Shi G. (2026). Multi-Scale Feature Extraction and Aggregation Network for Electroencephalography Classification in Face Photo-Sketch Recognition Task. IEEE Trans. Biomed. Eng..

[98] Soltani H., Einalou Z., Dadgostar M., Maghooli K. (2021). Classification of SSVEP-based BCIs using Genetic Algorithm. J. Big Data.

[99] Yap H.Y., Choo Y.H., Mohd Yusoh Z.I., Khoh W.H. (2021). Person authentication based on eye-closed and visual stimulation using EEG signals. Brain Inform..

[100] Chen Y., Shi X., De Silva V., Dogan S. (2024). Steady-State Visual Evoked Potential-Based Brain–Computer Interface System for Enhanced Human Activity Monitoring and Assessment. Sensors.

[101] El-Fiqi H., Wang M., Salimi N., Kasmarik K., Barlow M., Abbass H. (2018). Convolution Neural Networks for Person Identification and Verification Using Steady State Visual Evoked Potential. Proceedings of the 2018 IEEE International Conference on Systems, Man, and Cybernetics (SMC).

[102] Qin K., Wang R. (2021). SSVEP signal classification and recognition based on individual signal mixing template multivariate synchronization index algorithm. Biomed. Signal Process. Control.

[103] Cong F., Alluri V., Nandi A.K., Toiviainen P., Fa R., Abu-Jamous B., Gong L., Craenen B.G.W., Poikonen H., Huotilainen M. (2013). Linking Brain Responses to Naturalistic Music Through Analysis of Ongoing EEG and Stimulus Features. IEEE Trans. Multimed..

[104] de Albuquerque V.H.C., Damaševičius R., Tavares J.M.R.S., Pinheiro P.R. (2018). EEG-Based Biometrics: Challenges and Applications. Comput. Intell. Neurosci..

[105] Calma A.D., Triplett J., Vucic S., Yiannikas C. (2025). Somatosensory evoked potentials: Technique, interpretation and clinical applications. Pract. Neurol..

[106] Pfurtscheller G., Lopes da Silva F.H. (1999). Event-related EEG/MEG synchronization and desynchronization: Basic principles. Clin. Neurophysiol..

[107] Yang B., Li D., Ma B., Gu X., Kong D. (2021). Motor Imagery EEG Classification Method Based on Adaptive Decision Surface of LDA Classifier. ICBBB ’21: Proceedings of the 2021 11th International Conference on Bioscience, Biochemistry and Bioinformatics.

[108] Yu Z., Chen W., Zhang T. (2022). Motor imagery EEG classification algorithm based on improved lightweight feature fusion network. Biomed. Signal Process. Control.

[109] Ramadan R.A., Vasilakos A.V. (2017). Brain computer interface: Control signals review. Neurocomputing.

[110] Maiorana E. (2020). Deep learning for EEG-based biometric recognition. Neurocomputing.

[111] Zyma I., Tukaev S., Seleznov I., Kiyono K., Popov A., Chernykh M., Shpenkov O. (2019). Electroencephalograms during Mental Arithmetic Task Performance. Data.

[112] Palaniappan R. (2005). Identifying Individuality Using Mental Task Based Brain Computer Interface. Proceedings of the 2005 3rd International Conference on Intelligent Sensing and Information Processing.

[113] Sharma N., Sharma M., Singhal A., Vyas R., Malik H., Afthanorhan A., Hossaini M.A. (2023). Recent Trends in EEG-Based Motor Imagery Signal Analysis and Recognition: A Comprehensive Review. IEEE Access.

[114] Mulder T. (2007). Motor imagery and action observation: Cognitive tools for rehabilitation. J. Neural Transm..

[115] Das R., Maiorana E., Campisi P. (2018). Motor Imagery for Eeg Biometrics Using Convolutional Neural Network. Proceedings of the 2018 IEEE International Conference on Acoustics, Speech and Signal Processing (ICASSP).

[116] Liu H., Lou T., Zhang Y., Wu Y., Xiao Y., Jensen C.S., Zhang D. (2024). EEG-Based Multimodal Emotion Recognition: A Machine Learning Perspective. IEEE Trans. Instrum. Meas..

[117] Hatipoglu Yilmaz B., Yilmaz C.M., Kose C. (2026). EEG-based fusion approaches in multimodal emotion recognition: An in-depth review. Neurocomputing.

[118] Schalk G., Mcfarland D., Hinterberger T., Birbaumer N., Wolpaw J. (2004). BCI2000: A general-purpose Brain-Computer Interface (BCI) system. IEEE Trans. Biomed. Eng..

[119] Ortega-Rodríguez J., Gómez-González J.F., de Pablo E.P. (2025). Biometric Identification Based on EEG Using Fuzzy Logic: A Novel Approach with Metaheuristic Optimization. IEEE Trans. Dependable Secur. Comput..

[120] Keshishzadeh S., Fallah A., Rashidi S. (2018). Electroencephalogram Based Biometrics: A Fractional Fourier Transform Approach. ICBEA ’18: Proceedings of the 2018 2nd International Conference on Biometric Engineering and Applications.

[121] Delorme A., Rousselet G.A., Macé M.J.M., Fabre-Thorpe M. (2004). Interaction of top-down and bottom-up processing in the fast visual analysis of natural scenes. Cogn. Brain Res..

[122] Poulos M., Rangoussi M., Alexandris N., Evangelou A. (2002). Person Identification from the EEG using Nonlinear Signal Classification. Methods Inf. Med..

[123] Benomar M., Cao S., Vishwanath M., Vo K., Cao H. (2022). Investigation of EEG-Based Biometric Identification Using State-of-the-Art Neural Architectures on a Real-Time Raspberry Pi-Based System. Sensors.

[124] Kumar P., Dutta U., Sharma K., Ganesan R.A. (2022). Functional Connectivity Methods for EEG-based Biometrics on a Large, Heterogeneous Dataset. arXiv.

[125] Blankertz B., Muller K.R., Krusienski D., Schalk G., Wolpaw J., Schlogl A., Pfurtscheller G., Millan J., Schroder M., Birbaumer N. (2006). The BCI competition III: Validating alternative approaches to actual BCI problems. IEEE Trans. Neural Syst. Rehabil. Eng..

[126] Yang S., Deravi F., Hoque S. (2018). Task sensitivity in EEG biometric recognition. Pattern Anal. Appl..

[127] Campisi P., La Rocca D., Scarano G. (2012). EEG for Automatic Person Recognition. Computer.

[128] (2008). Brain–Computer Interface (BCI) Competition Organizers. BCI Competition IV. https://www.bbci.de/competition/iv/.

[129] Atyabi A., Shic F., Naples A. (2016). Mixture of autoregressive modeling orders and its implication on single trial EEG classification. Expert Syst. Appl..

[130] Tiwari A., Chaturvedi A. (2021). A Novel Channel Selection Method for BCI Classification Using Dynamic Channel Relevance. IEEE Access.

[131] Koelstra S., Mühl C., Soleymani M., Lee J.S., Yazdani A., Ebrahimi T., Pun T., Nijholt A., Patras I. (2012). DEAP: A Database for Emotion Analysis Using Physiological Signals. IEEE Trans. Affect. Comput..

[132] Gómez-Tapia C., Bozic B., Longo L. (2022). On the Minimal Amount of EEG Data Required for Learning Distinctive Human Features for Task-Dependent Biometric Applications. Front. Neuroinform..

[133] Carrión-Ojeda D., Martínez-Arias P., Fonseca-Delgado R., Pineda I., Mejía-Vallejo H. (2024). Evaluation of Features and Channels of Electroencephalographic Signals for Biometric Systems. EURASIP J. Adv. Signal Process..

[134] Mao Z., Yao W.X., Huang Y. (2017). EEG-based biometric identification with deep learning. Proceedings of the 2017 8th International IEEE/EMBS Conference on Neural Engineering (NER).

[135] Hollenstein N., Rotsztejn J., Troendle M., Pedroni A., Zhang C., Langer N. (2018). ZuCo, a simultaneous EEG and eye-tracking resource for natural sentence reading. Sci. Data.

[136] Kahana M., Lohnas L., Healey K., Aka A., Broitman A., Crutchley P., Crutchley E., Alm K., Katerman B., Miller N. (2024). The Penn Electrophysiology of Encoding and Retrieval Study. J. Exp. Psychol. Learn. Mem. Cogn..

[137] Yazici A., Kucukyilmaz T., Dokeroglu T., Sharipbay A., Lee M.H., Tyler B. (2026). State-of-the-art Multimodal Emotion Recognition: A comprehensive survey and taxonomy. Intell. Syst. Appl..

[138] Wang M., Wang S., Hu J. (2022). Cancellable Template Design for Privacy-Preserving EEG Biometric Authentication Systems. IEEE Trans. Inf. Forensics Secur..

[139] Liu H., Jin X., Liu D., Kong W., Tang J., Peng Y. (2024). Affective EEG-based Cross-Session Person Identification Using Hierarchical Graph Embedding. Cogn. Neurodyn..

[140] Becerra M.A., Duque-Mejía C., Castro-Ospina A., Serna-Guarín L., Duque-Grisales E. (2025). EEG-Based Biometric Identification and Emotion Recognition: An Overview. Computers.

[141] Usman M., Sultan N., Nasim A., Ayaz B., Fatima J., Nosheen F. (2026). Balancing Noise Reduction and Neural Signature Preservation in EEG Biometrics. Sci. Rep..

[142] Moreno-Rodríguez J.C., Atenco-Vázquez J.C., Ramírez-Cortés J.M., Arechiga-Martínez R., Gómez-Gil P., Fonseca-Delgado R. (2021). BIOMEX-DB: A Cognitive Audiovisual Dataset for Unimodal and Multimodal Biometric Systems. IEEE Access.

[143] Merit K., Beladgham M. (2024). Enhancing Biometric Security with Bimodal Deep Learning and Feature-level Fusion of Facial and Voice Data. J. Telecommun. Inf. Technol..

[144] Goudiaby B., Othmani A., Nait-ali A., Nait-ali A. (2020). EEG Biometrics for Person Verification. Hidden Biometrics: When Biometric Security Meets Biomedical Engineering.

[145] Fatourechi M., Bashashati A., Ward R.K., Birch G.E. (2007). EMG and EOG artifacts in brain computer interface systems: A survey. Clin. Neurophysiol..

[146] Khatun S., Mahajan R., Morshed B.I. (2016). Comparative Study of Wavelet-Based Unsupervised Ocular Artifact Removal Techniques for Single-Channel EEG Data. IEEE J. Transl. Eng. Health Med..

[147] Delorme A., Makeig S. (2004). EEGLAB: An open source toolbox for analysis of single-trial EEG dynamics including independent component analysis. J. Neurosci. Methods.

[148] Peng W., Hu L., Zhang Z. (2019). EEG Preprocessing and Denoising. EEG Signal Processing and Feature Extraction.

[149] Chen B., Cho K.W., Xu C., Li Z., Lin F., Jin Z., Xu W. (2020). A stimulus-response based EEG biometric using mallows distance. CCF Trans. Netw..

[150] Qu H., Zhao H., Wei Q., Pei W., Gao X., Wang Y. (2024). Combing Multiple Visual Stimuli to Enhance the Performance of VEP-Based Biometrics. IEEE Trans. Inf. Forensics Secur..

[151] Maiorana E., Campisi P. (2018). Longitudinal Evaluation of EEG-Based Biometric Recognition. IEEE Trans. Inf. Forensics Secur..

[152] Haukipuro E., Kolehmainen V., Myllarinen J., Remander S., Salo J., Takko T., Nguyen L., Sigg S., Findling R.D. (2019). Mobile Brainwaves: On the Interchangeability of Simple Authentication Tasks with Low-Cost, Single-Electrode EEG Devices. IEICE Trans. Commun..

[153] Bhateja V., Gupta A., Mishra A., Mishra A., Satapathy S.C., Bhateja V., Somanah R., Yang X.S., Senkerik R. (2019). Artificial Neural Networks Based Fusion and Classification of EEG/EOG Signals. Proceedings of the Information Systems Design and Intelligent Applications.

[154] Waili T., Johar M.G.M., Sidek K.A., Nor N.S.H.M., Yaacob H., Othman M. (2019). EEG Based Biometric Identification Using Correlation and MLPNN Models. Int. J. Online Biomed. Eng. (IJOE).

[155] Khalil A.E.K., Perez-Diaz J.A., Cantoral-Ceballos J.A., Antelis J.M. (2024). Unlocking security for comprehensive electroencephalogram-based user authentication systems. Sensors.

[156] Schons T., Moreira G., Silva P., Coelho V., Luz E. (2018). Convolutional Network for EEG-Based Biometric.

[157] Tsai M.H., Hsia C.Y., Wu S.K., Chen T.L. (2020). An Individual Specific Electroencephalography Signal Pattern Verification Model Based on Machine Learning and Convolutional Neural Network. J. Adv. Comput. Netw..

[158] Balcı F. (2023). DM-EEGID: EEG-based biometric authentication system using hybrid attention-based LSTM and MLP algorithm. Trait. Du Signal.

[159] Bandana Das B., Kumar Ram S., Sathya Babu K., Mohapatra R.K., Mohanty S.P. (2024). Person identification using autoencoder-CNN approach with multitask-based EEG biometric. Multimed. Tools Appl..

[160] Lakhan P., Banluesombatkul N., Sricom N., Sawangjai P., Sangnark S., Yagi T., Wilaiprasitporn T., Saengmolee W., Limpiti T. (2025). EEG-BBNet: A Hybrid Framework for Brain Biometric Using Graph Connectivity. IEEE Sens. Lett..

[161] Widmann A., Schröger E., Maess B. (2015). Digital filter design for electrophysiological data—A practical approach. J. Neurosci. Methods.

[162] Dash D.P., Kolekar M.H., Jha K. (2020). Multi-channel EEG based automatic epileptic seizure detection using iterative filtering decomposition and Hidden Markov Model. Comput. Biol. Med..

[163] Mu Z., Hu J., Min J., Yin J. (2017). Comparison of different entropies as features for person authentication based on EEG signals. IET Biom..

[164] Gorur K. (2023). Fourier Synchrosqueezing Transform-ICA-EMD Framework Based EOG-Biometric Sustainable and Continuous Authentication via Voluntary Eye Blinking Activities. Biomimetics.

[165] Gabard-Durnam L.J., Mendez Leal A.S., Wilkinson C.L., Levin A.R. (2018). The Harvard Automated Processing Pipeline for Electroencephalography (HAPPE): Standardized Processing Software for Developmental and High-Artifact Data. Front. Neurosci..

[166] He P., Kahle M., Wilson G., Russell C. (2005). Removal of Ocular Artifacts from EEG: A Comparison of Adaptive Filtering Method and Regression Method Using Simulated Data. Proceedings of the 2005 IEEE Engineering in Medicine and Biology 27th Annual Conference.

[167] Zhang G., Garrett D.R., Simmons A.M., Kiat J.E., Luck S.J. (2024). Evaluating the Effectiveness of Artifact Correction and Rejection in Event-Related Potential Research. Psychophysiology.

[168] Yao D., Qin Y., Dong L., Vega M., Sosa P. (2019). Which Reference Should We Use for EEG and ERP practice?. Brain Topogr..

[169] Yao D. (2001). A method to standardize a reference of scalp EEG recordings to a point at infinity. Physiol. Meas..

[170] Das R., Maiorana E., Campisi P. (2017). Visually evoked potential for EEG biometrics using convolutional neural network. Proceedings of the 2017 25th European Signal Processing Conference (EUSIPCO).

[171] Hu S., Yao D., Valdes-Sosa P.A. (2018). Unified Bayesian Estimator of EEG Reference at Infinity: RREST (Regularized Reference Electrode Standardization Technique). Front. Neurosci..

[172] Fan Y., Shi X., Li Q. (2021). CNN-Based Personal Identification System Using Resting State Electroencephalography. Comput. Intell. Neurosci..

[173] Soleymani M., Lichtenauer J., Pun T., Pantic M. (2012). A Multimodal Database for Affect Recognition and Implicit Tagging. IEEE Trans. Affect. Comput..

[174] Sun Y., Lo F.P.W., Lo B. (2019). EEG-based user identification system using 1D-convolutional long short-term memory neural networks. Expert Syst. Appl..

[175] Alsumari W., Hussain M., Alshehri L., Aboalsamh H.A. (2023). EEG-Based Person Identification and Authentication Using Deep Convolutional Neural Network. Axioms.

[176] Bigdely-Shamlo N., Mullen T., Kothe C., Su K.M., Robbins K.A. (2015). The PREP pipeline: Standardized preprocessing for large-scale EEG analysis. Front. Neuroinform..

[177] He B., Dai Y., Astolfi L., Babiloni F., Yuan H., Yang L. (2011). eConnectome: A MATLAB toolbox for mapping and imaging of brain functional connectivity. J. Neurosci. Methods.

[178] Singh A.K., Krishnan S. (2022). Trends in EEG signal feature extraction applications. Front. Artif. Intell..

[179] Pooja, Pahuja S., Veer K. (2022). Recent Approaches on Classification and Feature Extraction of EEG Signal: A Review. Robotica.

[180] Rajalakshmi A., Sridhar D. (2023). A Study of Time Domain Features of EEG Signal Analysis.

[181] Yang R., Zhang L., Peng Y., Zhong B., Hou L., Peng J., Xu B., Yang R. (2025). Evaluation of entropy features and classifier performance in person authentication using resting-state EEG. Front. Neurosci..

[182] Palaniappan R. (2008). Two-stage biometric authentication method using thought activity brain waves. Int. J. Neural Syst..

[183] Stancin I., Cifrek M., Jovic A. (2021). A Review of EEG Signal Features and Their Application in Driver Drowsiness Detection Systems. Sensors.

[184] Moctezuma L.A., Molinas M. (2018). EEG-based Subjects Identification based on Biometrics of Imagined Speech using EMD. arXiv.

[185] Garima, Goel N., Rathee N. (2024). Novel fractal pattern based features for EEG-based emotion identification. Biomed. Signal Process. Control.

[186] Monsy J.C., Vinod A.P. (2020). EEG-based biometric identification using frequency-weighted power feature. IET Biom..

[187] Gorur K., Olmez E., Ozer Z., Cetin O. (2023). EEG-Driven Biometric Authentication for Investigation of Fourier Synchrosqueezed Transform-ICA Robust Framework. Arab. J. Sci. Eng..

[188] del Pozo-Banos M., Travieso C.M., Weidemann C.T., Alonso J.B. (2015). EEG biometric identification: A thorough exploration of the time–frequency domain. J. Neural Eng..

[189] Palaniappan R., Mandic D.P. (2007). Biometrics from Brain Electrical Activity: A Machine Learning Approach. IEEE Trans. Pattern Anal. Mach. Intell..

[190] Harshit, Smitha K.G., Thomas K.P., Vinod A.P. (2016). Online Electroencephalogram (EEG) based biometric authentication using visual and audio stimuli. Proceedings of the 2016 IEEE EMBS Conference on Biomedical Engineering and Sciences (IECBES).

[191] Palaniappan R. (2006). Utilizing Gamma Band to Improve Mental Task Based Brain-Computer Interface Design. IEEE Trans. Neural Syst. Rehabil. Eng..

[192] Wu C., Yao B., Zhang X., Li T., Wang J., Pu J. (2025). The Application of Entropy in Motor Imagery Paradigms of Brain–Computer Interfaces. Brain Sci..

[193] Tian Y., Zhang H., Xu W., Zhang H., Yang L., Zheng S., Shi Y. (2017). Spectral Entropy Can Predict Changes of Working Memory Performance Reduced by Short-Time Training in the Delayed-Match-to-Sample Task. Front. Hum. Neurosci..

[194] Chiarion G., Sparacino L., Antonacci Y., Faes L., Mesin L. (2023). Connectivity analysis in EEG data: A tutorial review of the state of the art and emerging trends. Bioengineering.

[195] Li M., Zhang N. (2022). A Dynamic Directed Transfer Function for Brain Functional Network-Based Feature Extraction. Brain Inform..

[196] Ridouh A., Boutana D., Bourennane S. (2017). EEG Signals Classification Based on Time Frequency Analysis. J. Circuits Syst. Comput..

[197] Zeynali M., Seyedarabi H. (2019). EEG-based single-channel authentication systems with optimum electrode placement for different mental activities. Biomed. J..

[198] Svetlakov M., Hodashinsky I., Sarin K. (2022). Representation Learning for Electroencephalogram-Based Biometrics Using Holo-Hilbert Spectral Analysis. Pattern Recognit. Image Anal..

[199] Cohen M.X. (2014). Analyzing Neural Time Series Data: Theory and Practice.

[200] Boashash B. (2016). Time–Frequency Signal Analysis and Processing: A Comprehensive Reference.

[201] Koike-Akino T., Wang Y., Utiyama M.O., Ishwar P., Erdogmus D. (2016). High-Accuracy User Identification Using EEG Biometrics. Proceedings of the Annual International Conference of the IEEE Engineering in Medicine and Biology Society (EMBC).

[202] Behrouzi T., Hatzinakos D. (2022). Graph variational auto-encoder for deriving EEG-based graph embedding. Pattern Recognit..

[203] Leeuwis N., Yoon S., Alimardani M. (2021). Functional Connectivity Analysis in Motor-Imagery Brain Computer Interfaces. Front. Hum. Neurosci..

[204] Li H., Liu M., Yu X., Zhu J., Wang C., Chen X., Feng C., Leng J., Zhang Y., Xu F. (2023). Coherence based graph convolution network for motor imagery-induced EEG after spinal cord injury. Front. Neurosci..

[205] Gui Q., Jin Z., Xu W., Ruiz-Blondet M.V., Laszlo S. (2015). Multichannel EEG-based biometric using improved RBF neural networks. Proceedings of the 2015 IEEE Signal Processing in Medicine and Biology Symposium (SPMB).

[206] Yassine F.A., Abdelkader G. (2024). EEG-based Biometric Authentication Using Machine and Deep Learning Approachs: A Review. Proceedings of the 2024 8th International Conference on Image and Signal Processing and their Applications (ISPA).

[207] LeCun Y., Bengio Y., Hinton G. (2015). Deep learning. Nature.

[208] Rakhmatulin I., Dao M.S., Nassibi A., Mandic D. (2024). Exploring Convolutional Neural Network Architectures for EEG Feature Extraction. Sensors.

[209] Lawhern V.J., Solon A.J., Waytowich N.R., Gordon S.M., Hung C.P., Lance B.J. (2018). EEGNet: A compact convolutional neural network for EEG-based brain–computer interfaces. J. Neural Eng..

[210] Thomas K.P., Vinod A.P., Robinson N. (2017). Online Biometric Authentication Using Subject-Specific Band Power features of EEG. Proceedings of the 2017 International Conference on Cryptography, Security and Privacy.

[211] Ruiz-Blondet M.V., Jin Z., Laszlo S. (2016). CEREBRE: A novel method for very high accuracy event-related potential biometric identification. IEEE Trans. Inf. Forensics Secur..

[212] Wang M., Yin X., Zhu Y., Hu J. (2022). Representation Learning and Pattern Recognition in Cognitive Biometrics: A Survey. Sensors.

[213] Ergenoglu T., Demiralp T., Bayraktaroglu Z., Ergen M., Beydagi H., Uresin Y. (2004). Alpha rhythm of the EEG modulates visual detection performance in humans. Brain Res. Cogn. Brain Res..

[214] Narayan Y. (2021). Motor-Imagery based EEG Signals Classification using MLP and KNNClassifiers. Turk. J. Comput. Math. Educ. (TURCOMAT).

[215] Alzahab N.A., Iorio A.D., Baldi M., Scalise L. (2022). Effect of Auditory Stimuli on Electroencephalography-based Authentication. Proceedings of the 2022 IEEE International Conference on Metrology for Extended Reality, Artificial Intelligence and Neural Engineering (MetroXRAINE).

[216] Arif M., ur Rehman F., Sekanina L., Malik A.S. (2024). A comprehensive survey of evolutionary algorithms and metaheuristics in brain EEG-based applications. J. Neural Eng..

[217] Abdulrahman S.A., Alhayani B. (2023). A comprehensive survey on the biometric systems based on physiological and behavioural characteristics. Mater. Today Proc..

[218] Golmohammadi M., Harati Nejad Torbati A.H., Lopez de Diego S., Obeid I., Picone J. (2019). Automatic Analysis of EEGs Using Big Data and Hybrid Deep Learning Architectures. Front. Hum. Neurosci..

[219] Kim Y., Ryu J., Kim K.K., Took C.C., Mandic D.P., Park C. (2016). Motor Imagery Classification Using Mu and Beta Rhythms of EEG with Strong Uncorrelating Transform Based Complex Common Spatial Patterns. Comput. Intell. Neurosci..

[220] Chen D., Huang H., Bao X., Pan J., Li Y. (2023). An EEG-based attention recognition method: Fusion of time domain, frequency domain, and non-linear dynamics features. Front. Neurosci..

[221] Khalid B., Hassan A., Yasin M., Salman M., Butt M.F.U., Abdul W., Niazi I.K. (2025). A triple-shallow CNN with genetic algorithm channel selection method for classifying EEG complex limb movements. Biomed. Signal Process. Control.

[222] Chen J.X., Mao Z.J., Yao W.X., Huang Y.F. (2020). EEG-based biometric identification with convolutional neural network. Multimed. Tools Appl..

[223] Cortez S., Flores C., Andreu-Perez J. (2021). A Smart Home Control Prototype Using a P300-Based Brain–Computer Interface for Post-stroke Patients. Proceedings of the 5th Brazilian Technology Symposium—Emerging Trends, Issues, and Challenges in the Brazilian Technology.

[224] Pasini A. (2015). Artificial neural networks for small dataset analysis. J. Thorac. Dis..

[225] Abdullah M.K., Subari K.S., Loong J.L.C., Ahmad N.N. (2010). Analysis of effective channel placement for an EEG-based biometric system. Proceedings of the 2010 IEEE EMBS Conference on Biomedical Engineering and Sciences (IECBES).

[226] Jijomon C., Vinod A. (2021). Person-identification using familiar-name auditory evoked potentials from frontal EEG electrodes. Biomed. Signal Process. Control.

[227] Alvarado-González M., Fuentes-Pineda G., Cervantes-Ojeda J. (2021). A few filters are enough: Convolutional neural network for P300 detection. Neurocomputing.

[228] Xu D., He H., Xie Y., Zhang S., Li X., Chen X. (2023). An Analysis of Deep Learning Models in SSVEP-Based BCI: A Survey. Brain Sci..

[229] Zhang X., Yao L., Wang X., Monaghan J., McAlpine D., Zhang Y. (2021). A Survey on Deep Learning-Based Non-Invasive Brain Signals: Recent Advances and New Frontiers. J. Neural Eng..

[230] Wang Y., Zhang L., Xia P., Wang P., Chen X., Du L., Fang Z., Du M. (2022). EEG-Based Emotion Recognition Using a 2D CNN with Different Kernels. Bioengineering.

[231] Roy Y., Banville H., Albuquerque I., Gramfort A., Falk T.H., Faubert J. (2019). Deep Learning-Based Electroencephalography Analysis: A Systematic Review. J. Neural Eng..

[232] Emsawas T., Morita T., Kimura T., Fukui K.I., Numao M. (2022). Multi-kernel temporal and spatial convolution for EEG-based emotion classification. Sensors.

[233] Cho K., van Merriënboer B., Gulcehre C., Bahdanau D., Bougares F., Schwenk H., Bengio Y., Moschitti A., Pang B., Daelemans W. (2014). Learning Phrase Representations using RNN Encoder–Decoder for Statistical Machine Translation. Proceedings of the 2014 Conference on Empirical Methods in Natural Language Processing (EMNLP).

[234] Mohamed Y., Anter A.M., Zaky A.B. (2023). Recurrent Neural Networks (RNNs) to improve EEG-based person identification. Proceedings of the 2023 Intelligent Methods, Systems, and Applications (IMSA).

[235] Wang Z., Wang Y. (2025). Multi-branch GAT-GRU-transformer for explainable EEG-based finger motor imagery classification. Front. Hum. Neurosci..

[236] Li J., Li S., Pan J., Wang F. (2021). Cross-Subject EEG Emotion Recognition with Self-Organized Graph Neural Network. Front. Neurosci..

[237] Graña M., Morais-Quilez I. (2023). A review of Graph Neural Networks for Electroencephalography data analysis. Neurocomputing.

[238] Ashenaei R., Shirazi A.A.B. (2025). Graph Neural Network Classification in EEG-Based Biometric Identification: Evaluation of Functional Connectivity Methods Using Time-Frequency Metric. IEEE Access.

[239] Kipf T.N., Welling M. Semi-Supervised Classification with Graph Convolutional Networks. Proceedings of the 5th International Conference on Learning Representations, ICLR 2017.

[240] Palaniappan R., Mandic D.P. (2007). EEG Based Biometric Framework for Automatic Identity Verification. J. VLSI Signal Process. Syst..

[241] Akbarnia Y., Daliri M.R. (2024). EEG-based identification system using deep neural networks with frequency features. Heliyon.

[242] Aloui K., Nait-Ali A., Naceur M.S. (2018). Using brain prints as new biometric feature for human recognition. Pattern Recognit. Lett..

[243] Hema C.R., Elakkiya A. (2014). Biometric Identification using Electroencephalography. Int. J. Comput. Appl..

[244] Ashenaei R., Asghar Beheshti A., Yousefi Rezaii T. (2022). Stable EEG-Based biometric system using functional connectivity based on Time-Frequency features with optimal channels. Biomed. Signal Process. Control.

[245] de Ataide M., Patel K., Vetrekar N., Gad R. (2024). Influence of Evaluating Cross-Stimulation on the EEG Biometric Verification Performance: Benchmarking Study. Proceedings of the Fifteenth Indian Conference on Computer Vision, Graphics and Image Processing (ICVGIP 2024).

[246] Patel K., de Ataide M.L., Vetrekar N., Ramachandra R., Ferreira T., Gad R. (2025). Does Distinctiveness in Brain Signals of Parkinson’s Impact Biometric Verification Performance?. Proceedings of the 2025 13th International Workshop on Biometrics and Forensics (IWBF).

[247] Lin G., Zhu L., Ren B., Hu Y., Zhang J. (2021). Multi-Branch Network for Cross-Subject EEG-based Emotion Recognition. Proceedings of the 13th Asian Conference on Machine Learning (ACML 2021).

[248] Quan J., Li Y., Wang L., He R., Yang S., Guo L. (2023). EEG-based cross-subject emotion recognition using multi-source domain transfer learning. Biomed. Signal Process. Control.

[249] Knierim M.T., Bleichner M.G., Reali P. (2023). A Systematic Comparison of High-End and Low-Cost EEG Amplifiers for Concealed, Around-the-Ear EEG Recordings. Sensors.

[250] Xu L., Xu M., Ke Y., An X., Liu S., Ming D. (2020). Cross-Dataset Variability Problem in EEG Decoding with Deep Learning. Front. Hum. Neurosci..

[251] Bishop C.M. (2006). Pattern Recognition and Machine Learning.

[252] Adil M., Mumtaz S., Farouk A., Song H., Jin Z. (2026). BrainAuth: A Neuro-Biometric Approach for Personal Authentication. IEEE J. Biomed. Health Inform..

[253] Segalowitz S.J., Davies P.L. (2004). Charting the maturation of the frontal lobe: An electrophysiological strategy. Brain Cogn..

[254] Ranasinghe K.G., Kudo K., Casaletto K., Rojas-Martinez J.C., Syed F., Vossel K., Miller B.L., Rabinovici G.D., Kramer J.H., Rankin K.P. (2025). Neurophysiological Signatures of Ageing: Compensatory and Compromised Neural Mechanisms. Brain Commun..

[255] Watanabe Y., Shibata M., Tanaka T., Kenji I., Yuko H., Yohei K., Yukio K. (2024). Age-related changes in EEG signal using triple correlation values. Front. Hum. Neurosci..

[256] Kang J.H., Bae J.H., Jeon Y.J. (2024). Age-Related Characteristics of Resting-State Electroencephalographic Signals and the Corresponding Analytic Approaches: A Review. Bioengineering.

[257] Javaid H., Nouman M., Cheaha D., Kumarnsit E., Chatpun S. (2024). Complexity measures reveal age-dependent changes in electroencephalogram during working memory task. Behav. Brain Res..

[258] Nayana B.R., Pavithra M.N., Chaitra S., Bhuvana Mohini T.N., Stephan T., Mohan V., Agarwal N. (2025). EEG-based neurodegenerative disease diagnosis: Comparative analysis of conventional methods and deep learning models. Sci. Rep..

[259] Patel K., Gad R.S., de Ataide M.L., Vetrekar N., Ferreira T., Ramachandra R. (2025). Stimulus-Evoked Brain Signals for Parkinson’s Detection: A Comprehensive Benchmark Performance Analysis on Cross-Stimulation and Channel-Wise Experiments. Bioengineering.

[260] Li J., Zhang D., Lin W., Liu W. (2025). Using EEG Signals and AI to Predict Neurodegenerative Diseases. IEEE Access.

[261] Ahmed Z., Wali A., Shahid S., Zikria S., Rasheed J., Asuroglu T. (2024). Psychiatric disorders from EEG signals through deep learning models. IBRO Neurosci. Rep..

[262] Malhotra S., Shah R. (2015). Women and mental health in India: An overview. Indian J. Psychiatry.

[263] Choy T., Baker E., Stavropoulos K. (2022). Systemic Racism in EEG Research: Considerations and Potential Solutions. Affect. Sci..

[264] Chen X.J., Ba L., Kwak Y. (2020). Neurocognitive Underpinnings of Cross-Cultural Differences in Risky Decision Making. Soc. Cogn. Affect. Neurosci..

[265] Hokett E., Duarte A. (2019). Age and Race-Related Differences in Sleep Discontinuity Linked to Associative Memory Performance and Its Neural Underpinnings. Front. Hum. Neurosci..

[266] Anokhin A.P., Müller V., Lindenberger U., Heath A.C., Myers E. (2006). Genetic Influences on Dynamic Complexity of Brain Oscillations. Neurosci. Lett..

[267] Begleiter H., Porjesz B. (2006). Genetics of Human Brain Oscillations. Int. J. Psychophysiol..

[268] Henrich J., Heine S.J., Norenzayan A. (2010). The weirdest people in the world?. Behav. Brain Sci..

[269] Penner F., Wall K.M., Guan K.W., Huang H.J., Richardson L., Dunbar A.S., Groh A.M., Rutherford H.J.V. (2023). Racial disparities in EEG research and their implications for our understanding of the maternal brain. Cogn. Affect. Behav. Neurosci..

[270] Hernandez H., Baez S., Medel V., Moguilner S., Cuadros J., Santamaria-Garcia H., Tagliazucchi E., Valdes-Sosa P.A., Lopera F., OchoaGómez J.F. (2024). Brain health in diverse settings: How age, demographics and cognition shape brain function. NeuroImage.

[271] Shatwan I.M., Alzharani M.A. (2024). Association between Perceived Stress, Emotional Eating, and Adherence to Healthy Eating Patterns among Saudi College Students: A Cross-Sectional Study. J. Health Popul. Nutr..

[272] Stergiadis C., Kostaridou V.D., Veloudis S., Kazis D., Klados M.A. (2022). A Personalized User Authentication System Based on EEG Signals. Sensors.

[273] Liu B., Cao L., Fang T. (2025). Research on real-time security authentication method based on EEG data features. Appl. Math. Nonlinear Sci..

[274] Wu Q., Yan B., Zeng Y., Zhang C., Tong L. (2018). Anti-deception: Reliable EEG-based biometrics with real-time capability from the neural response of face rapid serial visual presentation. Biomed. Eng. OnLine.

[275] Minielly N., Hrincu V., Illes J. (2020). Privacy Challenges to the Democratization of Brain Data. iScience.

[276] Mccullagh P., Lightbody G., Żygierewicz J., Kernohan W. (2014). Ethical Challenges Associated with the Development and Deployment of Brain Computer Interface Technology. Neuroethics.

[277] Yuste R., Goering S., Arcas B.A.Y., Bi G., Carmena J.M., Carter A., Fins J.J., Friesen P., Gallant J., Huggins J.E. (2017). Four ethical priorities for neurotechnologies and AI. Nature.

[278] Ruiz S., Valera L., Ramos P., Sitaram R. (2024). Neurorights in the Constitution: From Neurotechnology to Ethics and Politics. Philos. Trans. R. Soc. B Biol. Sci..

[279] Jung T.P., Makeig S., Westerfield M., Townsend J., Courchesne E., Sejnowski T.J. (2000). Removal of eye activity artifacts from visual event-related potentials in normal and clinical subjects using ICA. Electroencephalogr. Clin. Neurophysiol..

[280] Radüntz T. (2018). Signal quality evaluation of emerging EEG devices. Front. Physiol..

[281] Trigka M., Dritsas E., Fidas C. (2023). A Survey on Signal Processing Methods for EEG-based Brain Computer Interface Systems. PCI ’22: Proceedings of the 26th Pan-Hellenic Conference on Informatics.

[282] Jalilpour S., Müller-Putz G. (2025). A framework for Interpretable deep learning in cross-subject detection of event-related potentials. Eng. Appl. Artif. Intell..

[283] Samek W., Wiegand T., Müller K.R. (2017). Explainable Artificial Intelligence: Understanding, Visualizing and Interpreting Deep Learning Models. arxiv.

[284] Arrieta A.B., Díaz-Rodríguez N., Del Ser J., Bennetot A., Tabik S., Barbado A., García S., Gil-López S., Molina D., Benjamins R. (2020). Explainable Artificial Intelligence (XAI): Concepts, taxonomies, opportunities and challenges toward responsible AI. Inf. Fusion.

[285] Tjoa E., Guan C. (2020). A survey on explainable artificial intelligence (XAI): Toward medical XAI. IEEE Trans. Neural Netw. Learn. Syst..

[286] Habashi A.G., Azab A.M., Eldawlatly S., Aly G.M. (2023). Generative adversarial networks in EEG analysis: An overview. J. Neuroeng. Rehabil..

[287] Bollens L., Francart T., Hamme H.V. (2022). Learning Subject-Invariant Representations from Speech-Evoked EEG Using Variational Autoencoders. Proceedings of the ICASSP 2022—2022 IEEE International Conference on Acoustics, Speech and Signal Processing (ICASSP).

[288] Nia A.F., Tang V., Talou G.M., Billinghurst M. (2024). Synthesizing affective neurophysiological signals using generative models: A review paper. J. Neurosci. Methods.

[289] Lashgari E., Liang D., Maoz U. (2020). Data augmentation for deep-learning-based electroencephalography. J. Neurosci. Methods.

[290] Acharya S., Khosravi A., Creighton D., Alizadehsani R., Acharya U.R. (2025). Neurostressology: A systematic review of EEG-based automated mental stress perspectives. Inf. Fusion.

[291] Lotte F., Bougrain L., Cichocki A., Clerc M., Congedo M., Rakotomamonjy A., Yger F. (2018). A review of classification algorithms for EEG-based brain–computer interfaces: A 10 year update. J. Neural Eng..

[292] Aldayel M., Alsedairy N., Al-Nafjan A., Alsenan S. (2024). Systematic Review of Brain-Computer Interface-Based User Authentication System: Trends, Challenges, and Directions. IEEE Access.

[293] Zhu H., Forenzo D., He B. (2022). On the Deep Learning Models for EEG-Based Brain-Computer Interface Using Motor Imagery. IEEE Trans. Neural Syst. Rehabil. Eng..

[294] Luo Y., Liu X., Yang M. (2026). Current status and future prospects of brain–computer interfaces in the field of neurological disease rehabilitation. Front. Rehabil. Sci..

[295] Alahaideb L., Alnafjan A., Aljumah H., Aldayel M. (2025). Brain–Computer Interface for EEG-Based Authentication: Advancements and Practical Implications. Sensors.

[296] Lotte F. (2015). Signal Processing Approaches to Minimize or Suppress Calibration Time in Oscillatory Activity-Based Brain–Computer Interfaces. Proc. IEEE.

